# A novel host-adapted strain of *Salmonella* Typhimurium causes renal disease in olive ridley turtles (*Lepidochelys olivacea*) in the Pacific

**DOI:** 10.1038/s41598-019-45752-5

**Published:** 2019-06-27

**Authors:** Thierry M. Work, Julie Dagenais, Brian A. Stacy, Jason T. Ladner, Jeffrey M. Lorch, George H. Balazs, Elías Barquero-Calvo, Brenda M. Berlowski-Zier, Renee Breeden, Natalia Corrales-Gómez, Rocio Gonzalez-Barrientos, Heather S. Harris, Gabriela Hernández-Mora, Ángel Herrera-Ulloa, Shoreh Hesami, T. Todd Jones, Juan Alberto Morales, Terry M. Norton, Robert A. Rameyer, Daniel R. Taylor, Thomas B. Waltzek

**Affiliations:** 1 US Geological Survey, National Wildlife Health Center, Honolulu Field Station, Honolulu, Hawaii 96850 United States of America; 2 NOAA Fisheries, Office of Protected Resources, University of Florida, Gainesville, Florida 32603 United States of America; 3 The Pathogen and Microbiome Institute, Northern Arizona University, Flagstaff, Arizona 86011 United States of America; 4 US Geological Survey, National Wildlife Health Center, Madison, Wisconsin 53711 United States of America; 5 Golden Honu Services of Oceania, Honolulu, Hawaii 96825 United States of America; 6 Escuela de Medicina Veterinaria (EMV), Universidad Nacional Costa Rica, Heredia, 3000 Costa Rica; 7 Parque Marino del Pacífico-Universidad Nacional Costa Rica, Puntarenas, Costa Rica; 8 Pathology Area National Service of Animal Health (SENASA), Ministry of Agriculture and Livestock, Heredia, 3000 Costa Rica; 9 NOAA Fisheries West Coast Region, Morro Bay, California United States of America; 10 Bacteriology Area, National Service of Animal Health (SENASA), Ministry of Agriculture and Livestock, Heredia, 3000 Costa Rica; 11 Department of Infectious Diseases and Immunology, College of Veterinary Medicine, University of Florida, Gainesville, Florida 32603 United States of America; 12 NOAA Fisheries, Pacific Islands Fisheries Science Center, Honolulu, Hawaii 96818 United States of America; 13 Georgia Sea Turtle Center/Jekyll Island Authority, Jekyll Island, Georgia, 31527 United States of America

**Keywords:** Pathogens, Microbial genetics

## Abstract

*Salmonella* spp. are frequently shed by wildlife including turtles, but *S. enterica* subsp. *enterica* serovar Typhimurium or lesions associated with *Salmonella* are rare in turtles. Between 1996 and 2016, we necropsied 127 apparently healthy pelagic olive ridley turtles (Lepidochelys olivacea) that died from drowning bycatch in fisheries and 44 live or freshly dead stranded turtles from the west coast of North and Central America and Hawaii. Seven percent (9/127) of pelagic and 47% (21/44) of stranded turtles had renal granulomas associated with *S*. Typhimurium. Stranded animals were 12 times more likely than pelagic animals to have *Salmonella*-induced nephritis suggesting that *Salmonella* may have been a contributing cause of stranding. *S*. Typhimurium was the only *Salmonella* serovar detected in *L. olivacea*, and phylogenetic analysis from whole genome sequencing showed that the isolates from *L. olivacea* formed a single clade distinct from other *S*. Typhimurium. Molecular clock analysis revealed that this novel clade may have originated as recently as a few decades ago. The phylogenetic lineage leading to this group is enriched for non-synonymous changes within the genomic area of Salmonella pathogenicity island 1 suggesting that these genes are important for host adaptation.

## Introduction

The olive ridley turtle (*Lepidochelys olivacea*) is one of the most widespread species of sea turtles, occurring in the Atlantic, Pacific, and Indian Oceans. Olive ridleys (*L. olivacea*) are primarily pelagic except when adults and juveniles occur in nearshore areas during breeding season^1^. In the Pacific, major nesting grounds are along the coasts of Mexico, Northern Costa Rica, and to a lesser extent in other Central American countries where nesting females aggregate. Olive ridleys are listed as “Vulnerable” by the International Union for the Conservation of Nature (IUCN). Breeding populations on the Pacific Coast of Mexico are listed as endangered, and all other populations are listed as threatened under the Endangered Species Act due to uncertain population trends and overexploitation of some subpopulations^2^.

Relatively little is known about infectious disease in *L. olivacea*^3^, in part because they spend most of their lives in the open ocean. Moreover, disease in *L. olivacea* is under-reported even in many coastal regions due to sparse resources, lack of organized stranding programs, or remoteness of coastlines^4^. However, since 1996, *L. olivacea* caught by the Hawaii based pelagic longline fishery have been routinely necropsied to gain a better understanding of health of pelagic sea turtles^5,6^. Additionally, since 2002 some stranded *L. olivacea* found on the Pacific coasts of the U.S and Central America, including during investigation of mass stranding events in Costa Rica, El Salvador, and isolated strandings in the Pacific Islands, have also been necropsied in attempts to understand potential causes of strandings. Multiple observations of granulomatous nephritis, including an unpublished case in 2012 from which *Salmonella enterica* subsp. *enterica* serovar Typhimurium was isolated, prompted our study of renal infections in *L. olivacea*.

Here, we show that renal pathology associated with *S*. Typhimurium is common in *L. olivacea* sampled from both coastal and oceanic environments in the Pacific. Moreover, the isolates of *S*. Typhimurium from *L. olivacea* are distinct from other known strains of the species. Our findings are striking for several reasons. First, *S*. Typhimurium is rarely recovered from turtles^7^. Second, host-adapted strains of *S*. Typhimurium are rare, so understanding the origin and virulence of this pathogen in *L. olivacea* might yield insights into other host-adapted salmonellae, some of which have relevance to human health. Third, salmonellosis appears to be a relatively frequent cause of disease in free-ranging Pacific *L. olivacea*. There is thus the potential that a host-adapted strain of *Salmonella* could contribute to future mortality events leading to conservation implications for certain vulnerable populations of *L. olivacea* such as those in the Pacific coast of Mexico that are listed as endangered.

## Results

### Salmonella Typhimurium causes significant pathology in pelagic and stranded turtles

**Figure 1:**
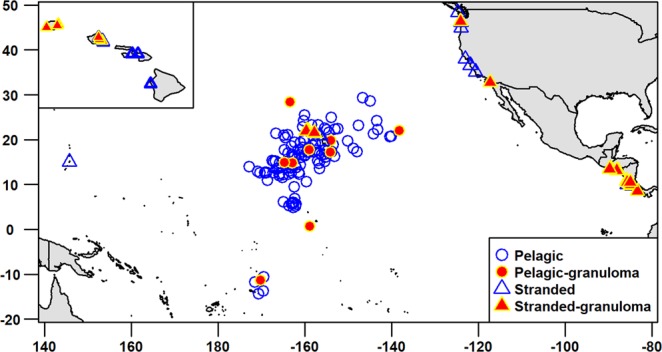
Distribution of pelagic and stranded olive ridley turtles in the Pacific. Stranded turtles in the north central Pacific are centered on the main Hawaiian Islands. Inset is map of Hawaiian islands with stranded animals with and without renal granulomas.

In total, 171 *L. olivacea* were necropsied between 1996 and 2016 of which 127 (74%) were pelagic turtles that died from forced submergence secondary to incidental catch^5,6^ in the north and south Pacific longline fishery. Of 44 stranded turtles, 16 were from Costa Rica spanning 2010 through 2014, six from El Salvador collected in 2014, and the remainder comprised 12 *L. olivacea* from Hawaii, four from California, three from Washington, two from Oregon, and one from Tinian Island (CNMI) spanning 2000 to 2016 (Fig. 1). Mean ± SD;N body condition index (BCI) of stranded turtles (0.000117 ± 1.985587e-05;N = 22) was significantly (W = 510, p = 3e-06) lower than that of bycaught pelagic turtles (0.00014 ± 3.0420e-05;N = 124). Mean straight carapace length (SCL) of stranded (51.8 ± 11.5; N = 22) and pelagic (55.4 ± 6.9; N = 127) turtles did not differ significantly (W = 990, p = 0.06). The age range of stranded vs. bycaught turtles was probably similar (based on SCL), but as expected, body condition of stranded animals was worse based on BCI.

**Figure 2:**
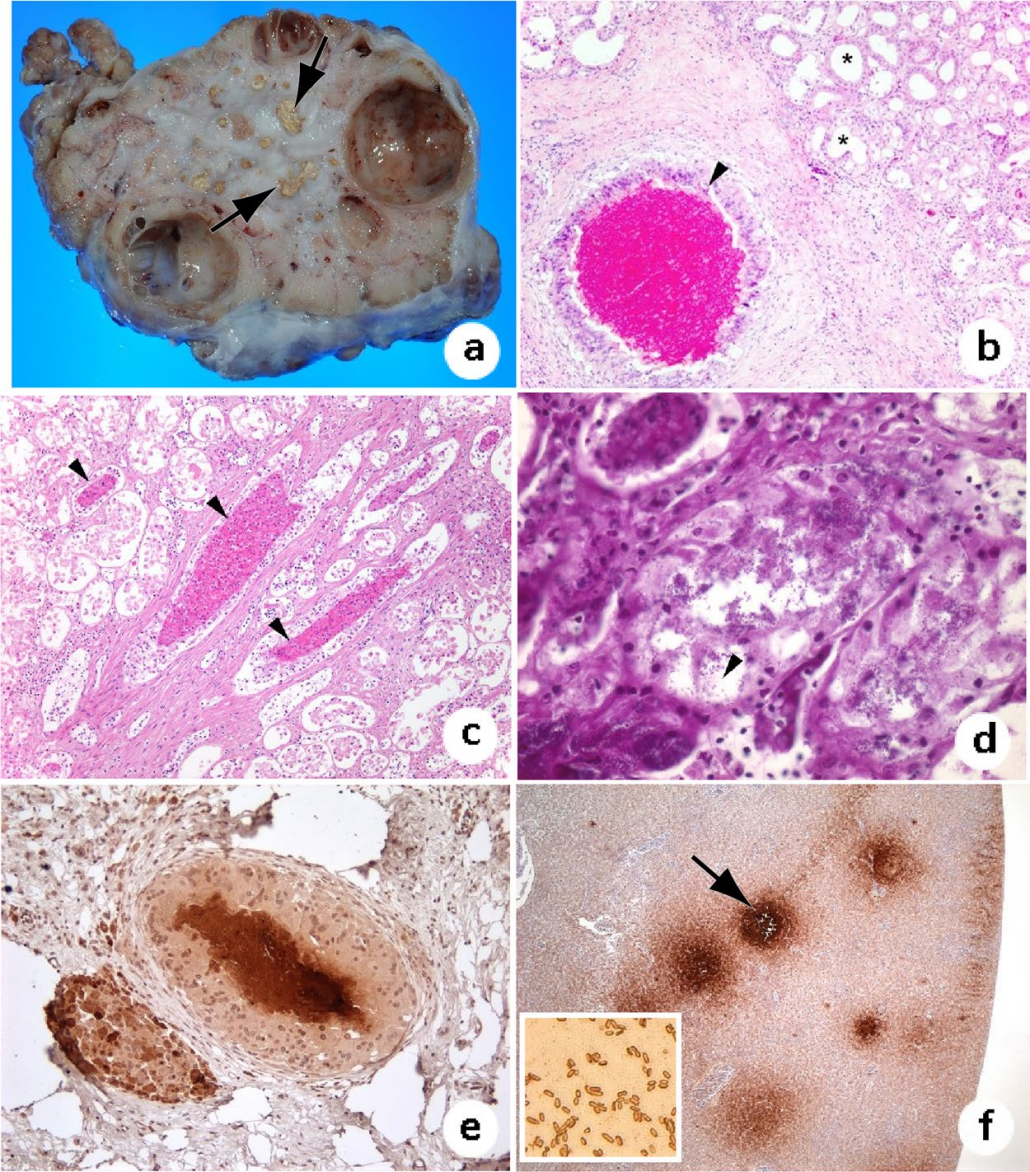
Granulomatous nephritis in olive ridleys. (**a**) Extensive fibrosis in the kidney of stranded turtle; note variably sized caseous nodules (granulomas) on cut surface (arrow). (**b**) Renal granuloma (arrowhead); note core of necrotic material surrounded by macrophages, including multinucleated giant cells, and a broad zone of fibrosis. There is secondary dilation of the surrounding renal tubules (asterisks). (**c**) Heterophils infiltrate multiple renal tubules (arrowheads). There is detachment of the renal epithelium and other artifacts resulting from autolysis. (**d**) Numerous gram-negative bacilli (arrowhead) within areas of nephritis from which *S*. Typhimurium was isolated. (**e**) Immunohistochemistry staining of renal granuloma with anti-*Salmonella* antibodies; note brown reactivity at the center of granulomas. (**f**) Positive control liver from a cattle egret with multifocal necrosis from which *S*. Typhimurium was cultured; note *S*. Typhimurium-positive foci (arrow). Inset: Pure culture of *S*. Typhimurium bacteria staining positive with anti-*S*. Typhimurium antibody.

The extent of renal lesions varied among *L. olivacea* with nephritis, and stranded animals had more severe lesions. Most of the renal granulomas were chronic as evidenced by extensive fibrosis. In severely affected turtles, the kidneys were bilaterally enlarged by chronic granulomas characterized as central areas of caseous material surrounded by fibrous connective tissue sometimes with associated fluid-filled cysts (Fig. 2a). Milder cases consisted of single or few isolated lesions where much of the kidneys were unaffected. By histology, the renal granulomas exhibited central areas of necrotic cellular material surrounded by macrophages with formation of multinucleated giant cells and fibroblasts (Fig. 2b). Some granulomas were accompanied by a more diffuse infiltrate of lymphocytes, macrophages, and heterophils. Associated tubular changes included dilatation, cyst formation, degenerate cellular casts, and mineralization (Fig. 2c). Heterophilic infiltrates were also observed within glomeruli in some cases, and in one case from Washington (USA), the granulomas were characterized by predominantly heterophilic inflammation that followed collected ducts (interpreted as ascending infection) and formed around numerous gram-negative bacilli (Fig. 2d). Chronic granulomas in another *L. olivacea* from this study exhibited a similar pattern. Significantly (p = 1.569e-08) more stranded *L. olivacea* (21/44, 47%) had renal granulomas compared to bycaught turtles (9/127, 7%) with stranded turtles being 12 times (odds ratio 95%CI = 4–33) more likely to have renal granulomas. We saw no significant difference (X^2^ = 1.6; p = 0.17) in prevalence of renal granulomas for males (4/47 or 8%) versus females (19/113 or 17%).

All eleven turtles with renal granulomas tested by immunohistochemistry (IHC) with anti-*Salmonella* antibodies were positive (Fig. 2e). Anti-*Salmonella* antibodies also decorated a *S*. Typhimurium-positive egret positive control liver with multifocal necrosis (Fig. 2f) and a pure culture of *S*. Typhimurium. All Salmonella located by IHC were extracellular. The same antibodies failed to decorate *E. coli*, *L. olivacea* kidneys without lesions or granulomatous lesions of a sea turtle attributed to other agents (fungi) based on culture or histology thereby confirming specificity of reaction.

### Salmonella Typhimurium isolated from olive ridley turtles represents a novel and potentially host-adapted strain

All five kidney tissues with granulomas tested by PCR were positive for the invA and SdiA genes of *Salmonella* whereas all four non-lesioned kidneys tested negative. Representative sequences for these genes were 100% identical with those of the type strain of *S*. Typhimurium (strain LT2). The same five tissues along with four additional lesioned tissues from stranded turtles were culture-positive for *Salmonella* whereas all four non-lesioned kidneys tested negative by PCR and were culture-negative. All isolates were confirmed as *S*. Typhimurium based on serotyping analyses.

**Figure 3:**
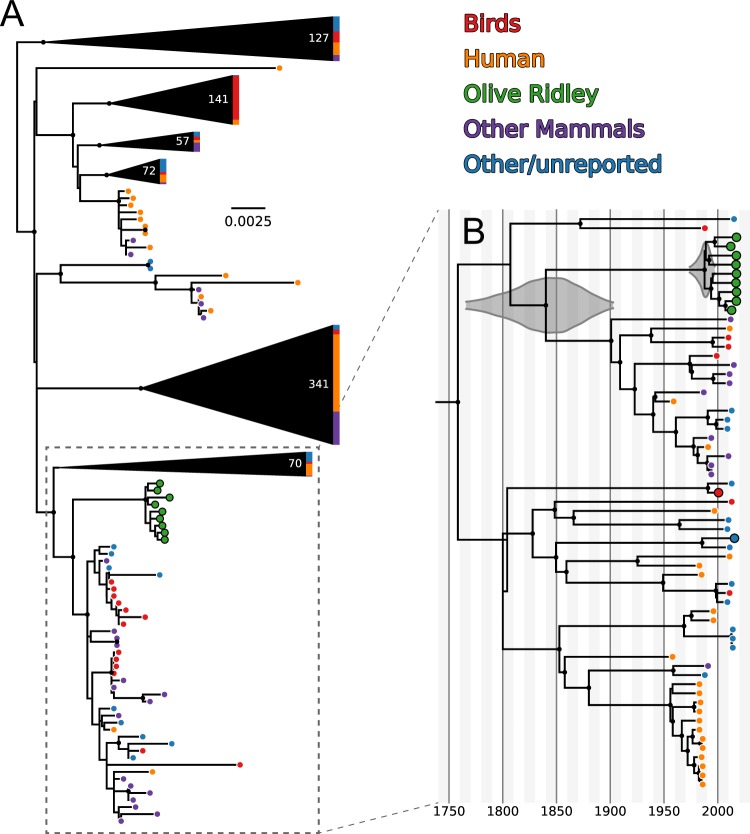
*Salmonella* Typhimurium isolates from olive ridley sea turtles form a novel phylogenetic clade. (**A**) Maximum-likelihood, whole genome SNP phylogeny including 877 strains of *S*. Typhimurium. The tree is midpoint rooted, and large clades have been collapsed for ease of visualization (number of collapsed tips annotated in white). Tip colors indicate the host type from which these strains were isolated and genomes generated in this study are indicated with black outlines. Black circles indicate nodes with ≥99% bootstrap support. (**B**) Time-structured Bayesian phylogeny of a subset of the strains included in (**A**). Gray distributions represent the 95% highest posterior probability density for the times of most recent common ancestor for the olive ridley clade as well as the olive ridley clade with its closest sister clade. Black circles indicate nodes with posterior probability ≥0.95.

Our serovar-level whole genome alignment included 5473 core SNPs and 877*S. enterica* genomes, including 13 generated in this study (Tables S1 and S2). Maximum-likelihood phylogenetic analysis demonstrated that all nine of the *L. olivacea*-derived *Salmonella* isolates belong to a well-supported monophyletic clade nested within, but distinct from, the *S*. Typhimurium diversity characterized from other mammalian, avian and reptilian host species (Fig. 3A). Our time-structured Bayesian phylogenetic analysis, which included 4952 SNPs and 61 isolates, confirmed the monophyletic nature of the *L. olivacea S*. Typhimurium clade. By fitting the genomic data to a molecular clock, we were able to estimate that the most recent common ancestor of this clade likely existed ~30 years ago (median: May 1987, 95% highest posterior density: June 1974 – December 1996). Similarly, we estimate that this *L. olivacea S*. Typhimurium clade split from its sister clade approximately 170 years ago (median: 1850, 95% highest posterior density: 1778–1900) (Fig. 3B). This sister clade included isolates from humans as well as several species of domesticated birds (chickens, turkeys) and mammals (pigs, cows, horses).

### The novel S. Typhimurium strain possesses unique genetic traits that may be associated with virulence

The phylogenetic branch leading (i.e. containing genetic changes specific) to the *S*. Typhimurium isolates from *L. olivacea* included 135 non-synonymous and 107 synonymous substitutions (Table S6). Consistent with positive selection, we observed a significantly higher than expected number of non-synonymous changes within both the *Salmonella* pathogenicity island 1 (SPI-1) and the Type 1 fimbrial gene cluster (Table 1). No such enrichment was observed within SPI-2 along the same branch or within any of the tested regions throughout the rest of the *S*. Typhimurium phylogeny (Table 1, Fig. 3B). Along the branch leading to the *L. olivacea S*. Typhimurium clade, we observed five non-synonymous changes in genes encoded within and/or effectors of the SPI-1 (one each in five different genes). These genes encode for three effector proteins (PipB2, AvrA and SipB: *AX05_34900*, *AX05_35810* and *AX05_36010*, respectively in GenBank:CP007523.1) and two transcriptional regulators (SirC and HilD: *AX05_35830* and *AX05_35910*, respectively). Similarly, we observed three non-synonymous changes within the type 1 fimbrial gene cluster: two in the FimH gene (*AX05_13030*) and one in the FimA gene (*AX05_12990*). The type III secretion system encoded by SPI-1 appears to be functional based on a comparison to the injectisome genes from S. Typhimurium strain LT2^8^. The LT2 proteins (obtained from UniProt) were compared to the de novo assembled genomes for two of our isolates, LOL16001 and LOL16002; all LT2 injectisome proteins had full matches in both of our isolates with a maximum of 1 AA mismatch between LT2 and our genomes.

**Table 1:** Tests for enrichment of synonymous and non-synonymous substitutions within Salmonella pathogenicity island (SPI) genes.

Functional Group^	Coding Bases*	Portion of Tree^^	Non-synonymous			Synonymous		
			Prop**	Odds Ratio	p-value	Prop**	Odds Ratio	p-value
SPI-1	34458/4048319	OR Branch	4/135	3.48	0.031	1/107	1.1	0.6
		Other Internal	6/1063	0.66	0.89	10/696	1.69	0.08
SPI-1 plus effectors	43381/4048319	OR Branch	5/135	3.46	0.017	2/107	1.74	0.32
		Other Internal	9/1063	0.79	0.8	11/696	1.47	0.14
SPI-2	35751/4048319	OR Branch	1/135	0.84	0.7	1/107	1.06	0.61
		Other Internal	13/1063	1.38	0.16	8/696	1.3	0.28
Type 1 fimbrial cluster	7899/4048319	OR Branch	3/135	11.39	0.0026	0/107	0	1
		Other Internal	2/1063	0.96	0.61	0/696	0	1

Odds ratios and p-values are from Fisher’s exact tests run in R v3.4.3.

Proportion of *total coding bases in the genome and **total substitutions contained within the functional group.

^See Table S5 for list of genes included in each functional group.

^^OR Branch = the single phylogenetic branch leading to the phylogenetic clade of isolates from olive ridley turtles (*L. olivacea*); Other Internal = the combination of all other internal (i.e., not leading to a tip) branches in the phylogeny, excluding any branches within the *L. olivacea* clade.

The *L. olivacea S*. Typhimurium isolates did not contain a virulence plasmid encoding the spv operon, which is strongly associated with non-typhoid, extra-intestinal disease caused by *Salmonella* in humans^9^. However, they did contain several unique gene regions that may contribute toward this strain’s distinct host preference and tropism. In total, we identified 373 genes putatively unique to the *L. olivacea* S. Typhimurium clade (Table S4). Despite the highly fragmented nature of our *de novo* genome assemblies, the vast majority of these genes occurred in clusters, consistent with the acquisition of large blocks through horizontal gene transfer. Based on PGAP annotations and top BLAST hits, two of the large clusters we identified appear to be associated with prophage (GenBank:QLZW00000000, DP141_01595-DP141_01825 and DP141_24510-DP141_24780), one resembles an integrative conjugative element (DP141_21750-DP141_22175) and one appears to encode a plasmid-associated type IV secretion system (DP141_17110-DP141_17715). Most of the genes contained in these regions have top BLAST hits to other Salmonella species, subspecies, serovars or strains (Table S4).

### The novel S. Typhimurium strain is a recent phenomenon in turtles with unknown origin

Within the *L. olivacea S*. Typhimurium clade we observed no genetic clustering based on collection date or location (Fig. S1). In fact, each of the isolates obtained from a stranded *L. olivacea* in the Eastern Pacific (all collected in 2012 or 2016) were most closely related to isolates obtained from *L. olivacea* sampled from the central North Pacific pelagic (all collected in 2017). Furthermore, none of the sampled isolates are genetically similar enough to constitute multiple cases from a single outbreak event. In fact, based on our molecular clock analysis, we estimated that even the most closely related of the sequenced *S*. Typhimurium isolates from *L. olivacea* diverged approximately five years prior to the oldest isolates (median: March 2007, 95% highest posterior density: May 2001 – April 2011).

**Figure 4:**
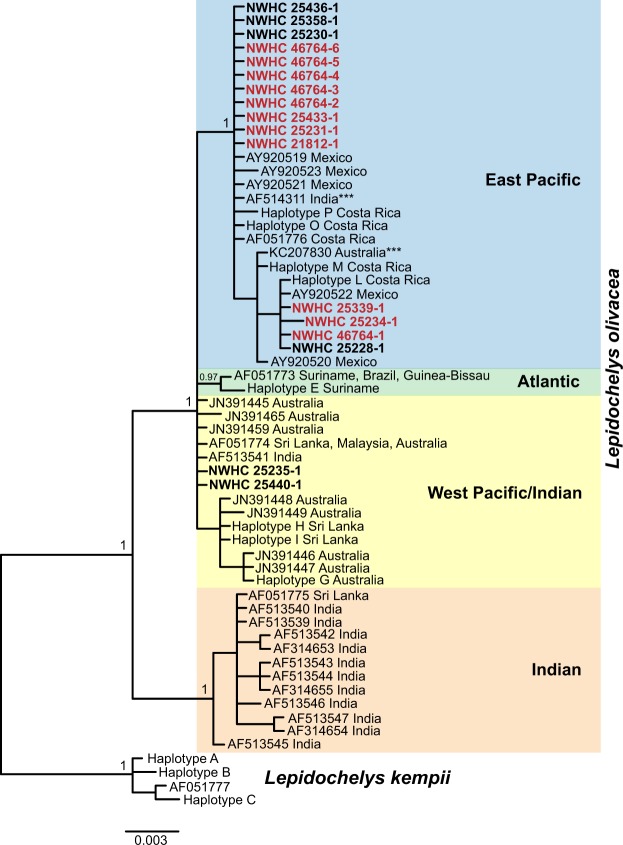
Bayesian phylogeny of mitochondrial control region sequences of stranded and pelagic olive ridleys (*Lepidochelys olivacea*). The turtles from nesting beaches are labeled with a GenBank accession number or haplotype name from^13^ and beach location. Strongly-supported clades representing populations that nest in disparate locations are shown, with apparent outliers marked with three asterisks. Turtles sampled for our study appear in bold, and those with salmonellosis are shown in red (N = 11). All turtles with *S*. Typhimurium infections resided in the clade that contains olive ridleys that nest primarily in the East Pacific (i.e., western coast of North America and Central America).

All 11 *L. olivacea* with salmonellosis that we genotyped grouped with nesting populations in the East Pacific (Fig. 4). Six of these 11 *L. olivacea* represented stranded turtles collected from the west coast of Central and North America (East Pacific), while the remaining five were taken from pelagic environments in the Pacific. Infected turtles represented four different haplotypes and could not be traced to a single nesting beach. However, a point source of exposure cannot be ruled out, because nesting beaches frequently contain turtles with multiple haplotypes^10–13^. Haplotypes more typical of *L. olivacea* populations that nest in Central America have occasionally been detected on nesting beaches in portions of the Indian Ocean^10,12^ further preventing any definitive conclusions about where infected turtles originated. The six turtles without salmonellosis that we genotyped were all collected from pelagic environments. Four of the individuals grouped with the East Pacific nesting populations and two with West Pacific nesting populations (Fig. 4) suggesting that *L. olivacea* from both East and West Pacific populations likely occurred in our sampling area. All newly-generated sequences were deposited in GenBank (accession numbers MK414426-MK414442).

## Discussion

Three lines of evidence support that the etiology of granulomatous nephritis in *L. olivacea* is renal salmonellosis caused by *S*. Typhimurium. First, *S*. Typhimurium was localized to lesions in all kidneys with granulomatous nephritis (including those with visible bacilli) when incubated with antibodies that were specific to *S*. Typhimurium. Second, *S*. Typhimurium was detected by PCR in DNA extracted directly from all tissues with lesions tested whereas unaffected tissues were PCR-negative. Finally, *Salmonella* isolates cultured from 9/9 tissues with lesions were confirmed by serotyping and/or genomic sequencing to be *S*. Typhimurium; the bacterium was not cultured from unaffected tissues.

Isolation of *S*. Typhimurium to the exclusion of other salmonellae was an unexpected finding in *L. olivacea*. This serotype is rarely encountered in reptiles^7^. We are only aware of a single example of disease caused by *S*. Typhimurium in a reptile; a case of granulomatous hepatitis in a captive spur-thigh tortoise (*Testudo graeca*)^14^. When *S. enterica* has been associated with disease in reptiles, either the subspecies has not been specified or other *S. enterica* subspecies (e.g., *S. enterica* subsp. Houtenae) or *S. enterica* subsp. *enterica* serovars (e.g., *S*. Derby, *S*. Arizonae), were isolated^15–17^.

Stranded *L. olivacea* were 12 times more likely to have renal salmonellosis than fisheries bycatch turtles. In fact, renal salmonellosis was the predominant finding in 21/44 stranded *L. olivacea* and we judged this as the most likely cause of stranding in these cases based on the degree of renal effacement. However, we cannot be certain whether this condition was the primary cause of the strandings or whether these may be opportunistic infections in animals with unrelated illnesses. Some turtles had concurrent abnormalities, such as fungal infections, suggesting some degree of immunosuppression, but the directionality of these relationships was unclear. Moreover, stranded turtles were in significantly worse body condition than pelagic animals. Immunosuppression as a precipitating cause of renal granulomas in pelagic turtles seems less likely as these animals were in good body condition and died from drowning from fisheries bycatch and not disease. Sorting out the role of *Salmonella*-induced nephritis as a precipitating cause of turtle strandings relative to host immune response would require either more systematic longitudinal studies of stranding events or controlled infection trials with captive healthy animals with known clinical histories. The latter is unlikely to be feasible in an imperiled, protected species.

Renal infections can be either blood-borne or ascending up the ureters^18^. In sea turtles, the ureters connect directly to the bowel lumen^19^, and in some cases inflammation was clearly centered on collecting ducts suggesting that infection was an ascending process, particularly in cases with enteritis. Whether this is true for all turtles requires future research. Culture of heart blood for *Salmonella* should help to answer whether some animals are septic leading to blood-borne dissemination to kidneys and subsequent inflammation. Renal granulomas have been documented in sea turtles, but usually associated with bacteria other than *Salmonella*^20^. Renal granulomas associated with *Salmonella* appears to be a phenomenon unique to *L. olivacea* which has not been documented in necropsy surveys of sea turtles elsewhere including green turtles from the Pacific^5,6,21^ and loggerheads from the Atlantic^17^.

All nine of the sequenced *S*. Typhimurium isolates from *L. olivacea* formed a well-supported monophyletic clade nested within, but distinct from, the known diversity of *S*. Typhimurium from terrestrial sources (primarily human-associated sources, including domesticated animals and associated agricultural products) (Fig. 3A). However, based on the level of genetic diversity among the isolates within this clade, the observed cases were not linked to a single outbreak strain. Rather, each sampled isolate appears to represent a more or less random draw from a pool of circulating diversity within the *L. olivacea S*. Typhimurium clade regardless of the location or year the animal was sampled. Combined, these results are consistent with two alternative scenarios: (1) various *S*. Typhimurium strains are endemic within the *L. olivacea* population and maintained through turtle-to-turtle transmission or (2) an alternate non-turtle reservoir serves as a source of repeated infection.

Based on molecular data, the *S*. Typhimurium clade (i.e., variant) isolated here appears to be host specific, only infecting *L. olivacea*. If true, this would be one of the few known host-adapted variants of *S*. Typhimurium. The only other example we are aware of is a variant associated with severe disease in pigeons^22^. However, while most *S*. Typhimurium variants are able to infect a wide variety of hosts^23^, many other *S*. enterica serovars do have more restricted host ranges. For instance, *S*. Typhi appears to infect only humans and higher primates^22^. One important caveat to the apparent host-adaptability of the *L. olivacea* variant is that the vast majority of sequenced isolates are from human-associated, terrestrial sources. A thorough survey of potential hosts within both neritic and pelagic environments would be needed to confirm *L. olivacea* as the sole or primary hosts of this variant.

If the *L. olivacea S*. Typhimurium variant is host specific, this variant likely adapted to the *L. olivacea* host within the last century (Fig. 3B). Known mechanisms explaining host-adaptation in bacteria are varied and include loss of gene function, diversifying selection, or gain of function through point mutation^24^. To further explore this hypothesis of adaptation to the *L. olivacea* host, we examined genetic changes that occurred along the phylogenetic branch leading to the *L. olivacea S*. Typhimurium clade. We found that this branch was significantly enriched for non-synonymous (i.e. amino acid changing) substitutions within genes that belong to the SPI-1 and those that encode type 1 fimbrial proteins. This finding is consistent with positive selection having acted on genomic regions involved in the interaction between bacteria and host.

Both type 1 fimbriae and SPI-1 are known to play important roles in bacterial invasion of host cells. Typically, the first step in *Salmonella* pathogenesis is attachment to intestinal mucosa, which, in *S*. Typhimurium, is generally mediated by type 1 fimbriae. FimA is the primary component of the type 1 fimbrial shaft, while FimH is the adhesin at the tip of the shaft. FimH is directly responsible for binding to eukaryotic cells and thus largely controls receptor specificity. Several studies have explored the impact of FimH allelic variation on the binding properties of type 1 fimbriae, and these studies have demonstrated that even single amino acid changes can result in major shifts in both cell type and host species affinity^25–27^. Therefore, the changes we observed in these two proteins in the *L. olivacea* isolates (two non-synonymous substitutions in FimH and one in FimA) may be involved in adaptation to this novel host species. SPI-1 encodes a type III secretion system (T3SS) that can deliver proteins from extracellular bacteria directly into host cells, and the SPI-1 T3SS and its effectors are known to play critical roles in host cell invasion and bacterial survival^28,29^. Therefore, SPI-1 genes are strong candidates for involvement in host adaptation. We also identified several clusters of genes that were likely acquired along this lineage through horizontal gene transfer (Table S4), though none have annotated roles in pathogenicity or host-adaptability. Follow-up experiments will be necessary to test the functional impact of these mutations and gene acquisitions.

Despite the presence of turtles from both East Pacific and West Pacific/Indian Ocean populations within bycatch from the North Pacific, host genotyping indicated that all of the *S*. Typhimurium infected animals likely nested along the coast of Central America (East Pacific). Although the number of *L. olivacea* we genotyped was relatively low (five infected pelagic turtles, six healthy pelagic turtles from fisheries, and six infected stranded turtles), host genetics suggest that there is a disparity in *S*. Typhimurium occurrence between East and West Pacific populations and that transmission likely occurs near the nesting habitat. However, it does not allow us to differentiate between turtle-to-turtle transmission, which may be associated with breeding- or nesting-specific behaviors, and other potential sources of exposure.

If the variant of *S*. Typhimurium we describe is endemic and *L. olivacea* are the reservoir, then infection likely occurs through the fecal oral route^30^. Nesting beach aggregations could provide one route of exposure when turtles come ashore in large numbers. For example, each year, thousands of turtles congregate to nest at Nancite Beach, Costa Rica^31^, and ca. 6% of these turtles have been shown to shed *Salmonella* (albeit non *S*. Typhimurium varieties) in the cloacal fluid^32^. Thus, opportunities exist for transmission of bacteria between nesting turtles. Finally, nesting densities of *L. olivacea* can be very high with many newly laid nests excavated by other nesting females leading to ruptured eggs promoting high microbial density in surrounding sand. Indeed, it is thought that this high microbial density may be partly responsible for low hatching success of this species^33^. To date, we know of no published data on *Salmonella* in beach sands.

Alternatively, if *S*. Typhimurium is not endemic to *L. olivacea*, then a potential source is seawater contaminated by other hosts. *Salmonella* are common in coastal watersheds impacted by urbanization^34^ or agriculture^35^ although such biogeographic associations are not consistent across regions^36^. Of all *Salmonella* species, *S*. Typhimurium dominates in coastal waters^36,37^. Moreover, *Salmonella* can remain viable in seawater for up to 32 weeks^38^. However, *Salmonella* obtained from coastal waters are thought to be primarily derived from human-associated terrestrial environments, and our *L. olivacea* variant is genetically distinct from any such isolates yet characterized.

Ingestion of *Salmonella* through prey is a final but less likely possibility for exposure to *S*. Typhimurium. *L. olivacea* in the coastal Pacific are carnivorous and have been reported to eat salps (gelatinous zooplankton), fish (presumably scavenged from fisheries discards), and various crustacea, bivalves, molluscs and other invertebrates^39^. The diet of *L. olivacea* in the pelagic environment, where the bycaught animals used in this study originated, is less known, although pelagic crustacea and salps have been documented in stomach contents^1,5,6^. Our understanding of *Salmonella* infecting these prey items is very limited. A survey of *Salmonella* in Brazil found large amounts of *Salmonella* near sewage outfalls but not in tissues of crabs in the immediate area^40^. *Salmonella* are also rare to uncommon in molluscs. For instance, *Salmonella* were rarely encountered in surveys of mussels, clams and worms in coastal California^41^, and surveys of 2980 clams and cockles in Spain revealed only 1.8% to be *Salmonella-*positive with *S*. Typhimurium comprising 18% of the *Salmonella* isolates^42^.

*S*. Typhimurium purportedly shed from reptiles has led to human disease outbreaks, particularly in association with small freshwater turtles^43^ however, the implications to human health from *S*. Typhimurium in wild *L. olivacea* are unclear. *Salmonella* originating from sea turtles could hypothetically pose a potential risk to human health either through consumption of meat or eggs^44,45^. However, reports of *Salmonella* outbreaks associated with consumption of sea turtle products are rare; the only ones we found were outbreaks of *S*. Chester associated with consumption of a green turtle in Papua New Guinea^46^ and *S*. Muenchen associated with consumption of green turtle in Northern Territory, Australia^47^. In Central America, nest depredation of *L. olivacea* eggs by humans for consumption is common, because turtle eggs are prized culinary items^48^, but we are not aware of reports of salmonellosis in humans due to consumption of turtle eggs. About 9% (8/90) of apparently healthy *L. olivacea* females in oceanic areas have *S*. Typhimurium-induced renal granulomas and presumably would be expected to shed *S*. Typhimurium once they reach nesting beaches. We judge this to be an underestimate, because we have no data on percent of non-granuloma females that are positive for *S*. Typhimurium. However, our calculated value is somewhat in line with the 6% shedding rate of non-Typhimurium *Salmonella* in nesting beaches in Nancite Costa Rica^32^ and the 4% *Salmonella* shedding rate detected in nesting *L. olivacea* from Las Baulas, Costa Rica^49^.

Twenty one of the renal salmonellosis cases presented here were part of mass stranding events in Central America^50^. Such wildlife mortality events are environmental signals that may have broader implications to ecosystem and possibly human health. These observations invite a need to investigate sporadic mortalities of *L. olivacea* in this region in a more systematic manner. Given the apparent chronicity of disease in these turtles, it remains unclear to what degree these mass events may have been driven by environmental conditions (i.e., wind and current) that favored synchronous beachcast stranding of floating, ill sea turtles. Understanding the mechanisms and environmental drivers of such events is critical to comprehensive understanding of the causes of sea turtle strandings.

## Methods

### Pathology & Epizootiology

All animals used in this study were recovered dead naturally or as a result of fishing bycatch activities. Because no live animals were used, IACUC does not apply. All samples were obtained under the following permits: National Oceanic and Atmospheric Administration National Marine Fisheries Permit 16865, US Fish and Wildlife Service endangered species permit BRD-VETAGENT-8, and CITES permit 17US105568/9. There was no filtering of animals, and we necropsied what was submitted to us. Freshly dead stranded *L. olivacea* were collected by sea turtle stranding networks, researchers, and government personnel on the Pacific coast of Costa Rica, El Salvador, Hawaii, Commonwealth of the Northern Marianas, and the continental United States under appropriate local and national permits. Pelagic *L. olivacea* were animals incidentally caught in fisheries originating from the National Oceanic and Atmospheric Administration, National Marine Fisheries Service observer programs of the North and South Pacific longline fishery^5,6^. Pelagic animals were considered “apparently healthy” based on lack of evident gross lesions and in good body condition. One hundred twenty-seven turtles incidentally caught in pelagic fisheries and 44 stranded turtles were necropsied and tissues were saved in 10% formalin for histologic examination. As part of the necropsy, carcasses were weighed to the nearest 0.1 kg and SCL was recorded to the nearest 0.1 cm. Body condition index (BCI, kg/SCL^3^) was calculated as described^51^. Our retrospective survey of previously examined *L. olivacea* focused on those animals in which the kidneys were evaluated by histology for evidence of renal granulomas, because these are not always visible grossly. Renal lesions were subjectively scored as mild (glomerulonephritis only, no fibrosis, no granulomas), moderate (focal to multifocal granulomas surrounded by fibroblasts with or without glomerulonephritis), or severe (diffuse effacement of renal architecture by connective tissue and fibroblasts with isolated to coalescing islands of necrosis surrounded by giant cells). We compared size and BCI of stranded and bycaught turtles using a non-parametric Wilcoxon rank-sum test after assessing lack of normality using the Shapiro-Wilks test. Fisher’s exact test was used to determine association between renal granulomas and stranding status. Alpha for all comparisons was <0.05. All analyses were done with R v. 3.4.1^52^.

For immunohistochemistry (IHC), tissues were deparaffinized in xylene and rehydrated in ethanol series (100%, 95%) followed by water. Heat retrieval was done in Tris-EDTA pH 9 in a steamer for 30 min. Tissues were then blocked with 3% H_2_O_2_ for 10 min followed by Dako Serum-free protein for 5 min. Primary antibody incubation was done for 30 min with mouse anti-*S*. Typhimurium monoclonal antibodies (Creative Diagnostics cat# DCABH-201852) diluted 1:1000 in Dako’s antibody diluent. After washing, tissues were incubated with goat anti-rabbit/mouse polymer conjugated to HRP (Vector Labs) for 30 min. After washing, color development was visualized with diaminobenzidine (Dako), and tissues were counterstained with hematoxylin (Vector Labs) prior to dehydration in reverse alcohol series and clearing with xylene. Tissues were then mounted in cytoseal (Richard-Allan Scientific) with a coverslip. To ensure the specificity of anti-*Salmonella* monoclonal antibodies, pure cultures of *E. coli* (ATCC 25922) and *S*. Typhimurium (ATCC 14028) grown overnight on blood agar at 37 °C were smeared onto glass slides, dried, fixed in methanol for 5–10 min, and reacted with anti-*Salmonella* antibodies as above. Positive control tissues were a liver from an egret (*Bubulcus ibis*) that had histologic lesions characteristic of salmonellosis and from which a pure culture of *S*. Typhimurium was isolated. Negative control tissues were *L. olivacea* kidneys with no microscopic lesions and lung tissue from a Kemp’s ridley turtle (*Lepidochelys kempii*) with fungal granulomatous pneumonia. The latter was included to control for the possibility of non-specific binding of antibodies to necrotic tissues of sea turtles.

### PCR & Culture

To detect *Salmonella* DNA, PCR was done as described previously^53^ on five and four granuloma positive and negative tissues respectively for which frozen samples were available. DNA was extracted from tissues using the Qiagen DNeasy Blood and Tissue Kit (Qiagen Inc., Valencia, California, USA) according to the manufacturer’s instructions. PCR was done using two sets of primers. One set of primers (5′ GTG AAA TTA TCG CCA CGT TCG GGC AA-3′ Forward; 5′-TCA TCG CAC CGT CAA AGG AAC C-3′ Reverse) targeted the *invA* virulence gene (Gene ID 1254419), which has been used to screen wildlife tissues for *Samonella*^54^. We also tested tissues using primers (5′-AAT ATC GCT TCG TAC CAC-3′ Forward, 5′-GTA GGT AAA CGA GGA GCA G-3′ Reverse) targeting the *Salmonella sdiA* quorum sensing gene (transcriptional regulator Gene ID 1253471) of *Salmonella* as a confirmatory PCR. Our PCR protocol included a 95 °C denaturing step, 35 cycles of 30 s at 94 °C, 30 s at 59 °C, 30 s at 72 °C and a final extension at 72 °C for 10 min. Expected products (274 bp for *sdiA* and 284 bp for *invA*) were analyzed by 1.5% agarose gel electrophoresis with appropriate molecular weight ladders. Gels were stained post-PCR with ethidium bromide and imaged using a Gel Doc EZ Imager (Bio-Rad). To verify the expected products (274 bp for *invA*, 284 bp for *sdiA*), representative reaction products were cleaned with ExoSAP-IT (Applied Biosystems) and sequenced in both directions using Sanger sequencing (Advanced Studies in Genomics, Proteomics and Bioinformatics (ASGPB), College of Natural Sciences, Univ. of Hawaii). Sequences were queried using BLAST^55^.

Two culture methods were used to isolate *Salmonella*. For four tissue samples obtained from Costa Rica and the continental U.S., swabs were collected from the centers of aseptically incised renal granulomas, applied to tetrathionate broth with iodine and Xylose Lysine Tergitol-4 (XLT4) agar, and grown at 37 °C. Five granuloma positive and four granuloma negative Pacific island cases testing PCR positive and negative for *Salmonella* spp., respectively, were selectively cultured by inoculating previously frozen tissues on XLT4 and Miller Mallinson agar plates. The plates were incubated at 37 °C for 24 hours after which isolated colonies resembling *Salmonella* spp. were transferred to fresh XLT medium. In Pacific island cases for which this method failed to produce isolates, remaining tissues were inoculated in Rappaport-Vassiliadis R10 (RV) enrichment broth and incubated at 42 °C. After 24 hours, a loopful of broth was streaked onto XLT4 and Miller Mallinson agar media and incubated for 24 hours at 37 °C. Isolates were identified by sequencing a portion of the 16S rRNA gene containing the V1–V3 regions^56^. The isolates were subsequently typed using the Kauffman-White method^57^ at the National Veterinary Services Laboratories (NVSL; Ames, IA).

### Genome sequencing and analysis

For extraction of DNA for whole genome sequencing, isolates were grown on tryptic soy agar containing 5% sheep blood at 37 °C for 24 hours. Isolated colonies were then inoculated into brain heart infusion broth and incubated overnight at 37 °C on a shaker (200 rpm). DNA was extracted from cell pellets using a phenol-chloroform extraction. Five *L. olivacea*-derived *Salmonella* spp. isolates were sequenced at the Pathogen and Microbiome Institute at Northern Arizona University and four were sequenced at the Wildlife and Aquatic Veterinary Disease Laboratory at the University of Florida, Gainesville. For comparative purposes, four non-*L. olivacea* wildlife isolates of *S*. Typhimurium were also sequenced at Northern Arizona University (Table S1). Whole genome shotgun sequencing libraries were prepared and sequenced on an Illumina MiSeq. Adapters were removed using Cutadapt v1.6^58^. Prinseq-lite v0.20.4 was used to filter out low quality reads and bases (-min_len 40 -trim_qual_left 15 -trim_qual_right 15 -trim_qual_type min -trim_qual_window 1 -min_qual_mean 20)^59^. *De novo* genome assemblies were generated for each isolate using SPAdes v3.10.1^60^ with default parameters, and these assemblies were annotated using PGAP v#5 (NCBI). Single-nucleotide polymorphisms (SNPs) were identified using NASP v1.02^61^ with Bowtie2 v2.2.8^61,62^ used for mapping Illumina reads and GATK v3.3–0 used for identifying SNPs (CoverageFilter = 3, ProportionFilter = 0.9). SNPs identified within duplicate regions of the reference genome were ignored. Identified SNPs were annotated using SnpEff v4.3t^63^.

For the serovar-level phylogeny, we used *S*. Typhimurium str. USDA-ARS-USMARC-1880 GenBank:GCA_001623705.1 as a reference genome. We downloaded all of the *S. enterica* genome assemblies present in GenBank (on 01/21/2018), which included one of the following in the organism name: “Typhimurium”, “Copenhagen” or “4_5_12_i” (Table S2). An initial run of NASP was conducted with just the GenBank assemblies in order to identify and remove duplicates. Assemblies were considered duplicates if they exhibited the same genotype across all of the “best^61^” SNPs (i.e., core SNPs not present within a duplicate region) and if they came from the same broad host type (e.g., human, porcine, bovine, avian). Several of the assemblies were also removed because they were high divergence outliers, indicative either of taxonomic misclassification or assembly errors. GenBank isolates were provided to NASP as assemblies, while isolates sequenced in this study were provided as NGS reads. An alignment was generated including all “best” variant sites identified by NASP and RAxML-NG (doi:10.5281/zenodo.593079) was used to generate a maximum likelihood phylogeny using the general time-reversible model with a discrete GAMMA model of rate heterogeneity (4 categories), 20 randomized starting trees, and 100 bootstrap replicates.

BEAST v1.8.4^64^ was used to generate a time-structured phylogeny including only a subset of the GenBank isolates from the serovar-level phylogeny (Fig. 3B). GenBank isolates were only included in this analysis if (1) they belonged to one of the lineages adjacent to the *L. olivacea* isolates (dashed box in Fig. 3A), (2) if a collection date was available and (3) if Illumina data were available in the GenBank Sequence Read Archive (SRA). All SRA data were downloaded using fastq-dump v2.8.2 (–gzip–skip-technical–readids–read-filter pass–dumpbase–split-files–clip–origfmt) and quality filtered as described above. For this subset analysis, NASP was run as described above, but with Illumina reads as the starting point for all isolates and with GenBank:GCA_000973645.1 as the reference. Sites were included in the alignment if (1) they were not located in duplicate regions within the reference, (2) genotypes were called for at least 70% of *L. olivacea* isolates and at least 90% of GenBank isolates and (3) they were not included in predicted recombinant regions identified using ClonalFrameML with default parameters^65^. The alignments included only variable positions, but the number of invariant sites, by nucleotide, were specified in the BEAST xml. Six different combinations of molecular clock and coalescent models were evaluated (Table S3) using path-sampling and stepping-stone marginal likelihood estimation approaches^66–68^. Each model combination was run with 500 million Markov chain Monte Carlo steps, sampling parameters and trees every 50,000 generations. The best fit model combination was an uncorrelated relaxed molecular clock with log normally distributed rate categories^69^, along with the nonparametric Bayesian SkyGrid tree prior with 20 parameters^70^ (BEAST XML available in Supplemental Material).

We also did a maximum-likelihood phylogenetic analysis, as described above, for the subset data set used for our BEAST analysis. TreeTime (https://github.com/neherlab/treetime) was used to map mutations onto branches of this phylogeny. We used Fisher’s exact test in R v3.4.3 to test for the enrichment of synonymous and non-synonymous substitutions within several portions of the genome known to be involved in virulence: *Salmonella* pathogenicity islands (SPI) 1 and its associated effectors, SPI2, and the type 1 fimbrial gene cluster (Table S5). We looked for enrichment of substitutions within these regions along the branch leading to the *L. olivacea*-specific clade, as well as along all other internal branches combined (excluding all *L. olivacea*-associated branches). We also examined amino acid changes in the injectisome of S. typhimurium, a complex structure encoded by multiple genes that is important in host cell attachment and invasion and thus virulence^71^.

To identify genes that were unique to the *L. olivacea*-specific clade, we used NASP to align the same subset dataset used for the BEAST analysis against our *L. olivacea* isolate LOL16001 (GenBank: QLZX00000000). *L. olivacea*-specific sites were identified as those for which (1) a genotype was called for at least 70% of *L. olivacea* isolates and (2) a genotype was not called for any non-*L. olivacea* isolates. Both variable and non-variable sites were included in this analysis. *L. olivacea*-specific genes were identified as those for which at least 40% of the contained nucleotide positions were determined to be *L. olivacea*-specific. Finally, NASP was used to identify variable sites within the *L. olivacea*-specific clade (GenBank: QLZX00000000 as reference). PopART (http://popart.otago.ac.nz/) was used to generate a median-joining haplotype network using the ‘best’ SNPs from NASP. Baltic (https://github.com/evogytis/baltic) was used for the visualization of phylogenies. Jupyter notebooks for generating the phylogenies are available at https://github.com/jtladner/Manuscripts/tree/master/2018_Work_ORSt.

### Host Genetics

Genetic analyses of *L. olivacea* sampled at nesting beaches have demonstrated population structure throughout the species’ range^13^. For example, populations nesting on the Pacific coast of Central America (i. e., East Pacific) exhibit haplotypes that are largely distinct from populations that nest in the West Pacific and Indian Oceans; however, adults may range far from their nesting beaches^10,12,13^. Many of the turtles examined in our study were taken from pelagic environments far from known nesting sites. As such, the origin of these turtles was unknown. To determine if the *Salmonella* infections were associated with particular *L. olivacea* populations, we conducted a genetic analysis on a haphazardly selected subset of turtles. We extracted DNA from kidney tissue of 11 *L. olivacea* with salmonellosis (five from pelagic environments and six that were stranded) and six apparently healthy *L. olivacea* using the Gentra®Puregene® Tissue Kit (Qiagen Inc., Valencia, California, USA) according to the manufacturer’s instructions. We amplified and sequenced an approximately 880 bp portion of the mitochondrial control region as described^12^, except that the number of PCR cycles was increased to 40.

We compared sequences generated in our study with those previously published^10–13^ to determine the likely locations that the turtles were born/nested. Sequences were aligned using ClustalW in MEGA v. 6^72^, and all gapped portions of the alignment were deleted. A Bayesian analysis from a final alignment of 389 characters was performed in MrBayes v. 3.2.6^73^ via the CIPRES Science Gateway^74^ using an HKY substitution model with a gamma distribution. The 50% majority rule consensus tree was generated with two runs, each with 5,000,000 generations and four chains. Chains were sampled every 1,000 generations with the first 25% discarded as burn-in.

## Supplementary information

Supplementary Information

## Data Availability

Data are available from USGS at 10.5066/P9O4NDD2 and additional links within this manuscript.
