# Supplementary material for: A novel host-adapted strain of Salmonella Typhimurium causes renal disease in olive ridley turtles (Lepidochelys olivacea) in the Pacific
Source: Sci Rep. 2019 Jun 27;9:9313. doi: 10.1038/s41598-019-45752-5 (PMC6597722; doi:10.1038/s41598-019-45752-5)
Supplement: Supplementary file 1 — Supplementary Information [file 41598_2019_45752_MOESM1_ESM.pdf]

SUPPLEMENTARY INFORMATION

A novel host-adapted strain of *Salmonella* Typhimurium causes renal disease in olive ridley turtles (*Lepidochelys olivacea*) in the Pacific

Thierry M. Work, Julie Dagenais, Brian A. Stacy, Jason T. Ladner, Jeffrey M. Lorch, George H. Balazs, Elías Barquero-Calvo, Brenda M. Berlowski-Zier, Renee Breeden, Natalia Corrales-Gómez, Rocio Gonzalez-Barrientos, Heather S. Harris, Gabriela Hernández-Mora, Ángel Herrera-Ulloa, Shoreh Hesami, T. Todd Jones, Juan Alberto Morales, Terry M. Norton, Robert A. Rameyer, Daniel R. Taylor,Thomas B. Waltzek.

Figure S1. *S.* typhimurium isolates do not cluster according to location or year of collection. Median-joining haplotype network constructed using PopART v1.7.2 and based on 227 variable sites among the 9 *L. olivacea S.* typhimurium whole genome sequences. Each circle represents an isolate. The inside color of each circle (light or dark blue) indicates where each infected animal was sampled and the outer color (yellow, orange, or red) indicates when each animal was sampled. Each tick along a branch represents a single variable site.

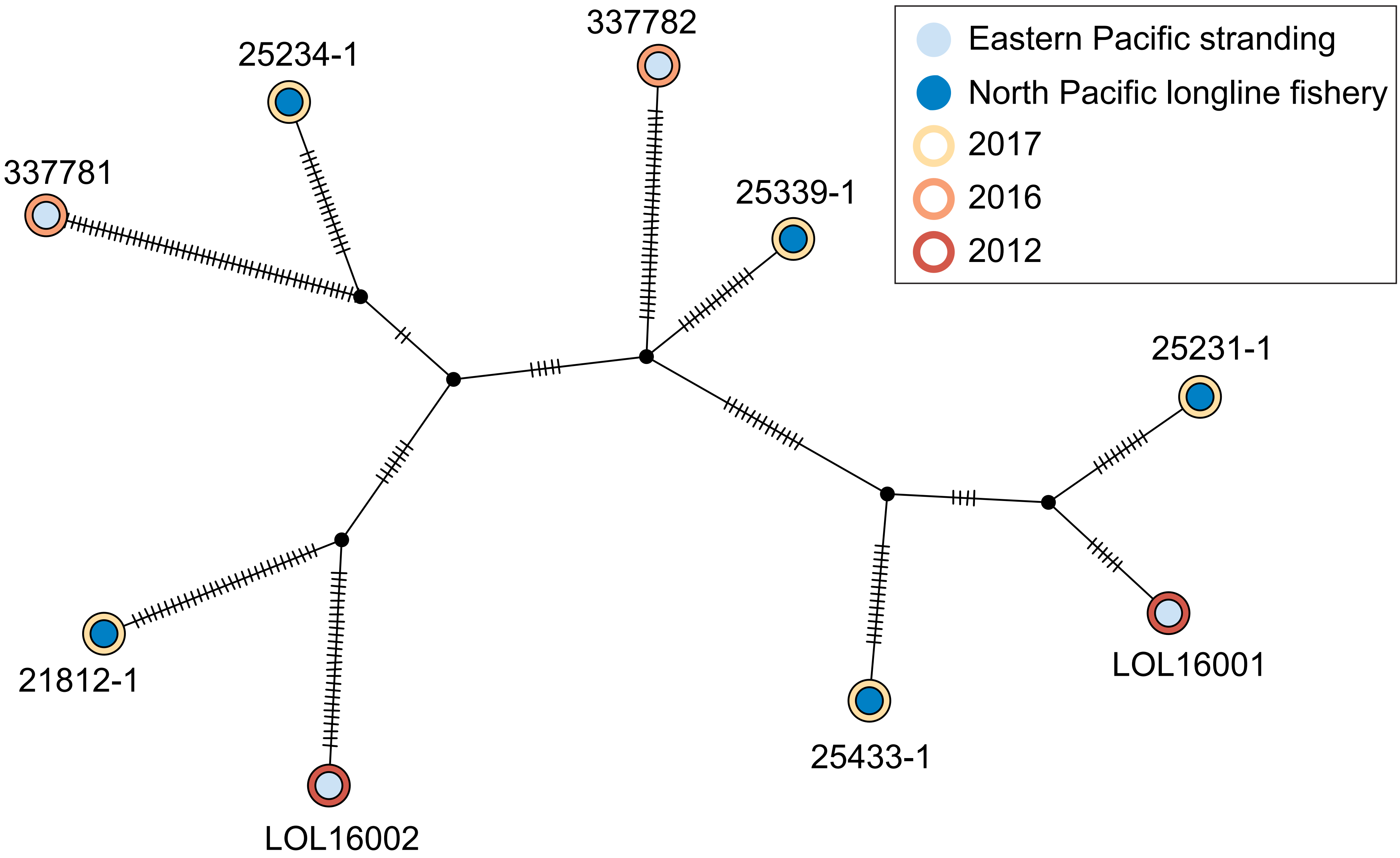
Eastern Pacific stranding
North Pacific longline fishery
2017
2016
2012
337782
25234-1
337781
25339-1
25231-1
21812-1
LOL16001
25433-1
LOL16002

Table S1. Metadata for isolates sequenced in this study.								
**Sample Name**	**Isolate**	**Collection Date**	**Location**	**Tissue**	**Host Species**	**Sequencing Location**	**Assembly Accession**	**SRR Accession**
STUF-LOL16001	LOL16001	2012-12-08	Costa Rica: Playa Puntarenas, Punta Arenas	kidney	Lepidochyelys olivacea	University of Florida	QLZX00000000	SRR7469200
STUF-LOL16002	LOL16002	2012-02-12	USA:Ocean Beach, California	kidney	Lepidochyelys olivacea	University of Florida	QLZW00000000	SRR7469199
STUF-337782	337782	2016-12-07	USA:Clallam, Washington		Lepidochyelys olivacea	University of Florida	QLZU00000000	SRR7469195
STUF-337781	337781	2016-12-27	USA:Long Beach, Washington		Lepidochyelys olivacea	University of Florida	QLZV00000000	SRR7469206
STNAU-21812-1	21812-1	2017-06-02	Pacific Ocean: Near Hawaii	kidney	Lepidochyelys olivacea	Northern Arizona University	QNQY00000000	SRR7469201
STNAU-25231-1	25231-1	2017-04-26	Pacific Ocean: Near Hawaii	kidney	Lepidochyelys olivacea	Northern Arizona University	QNQX00000000	SRR7469194
STNAU-25234-1	25234-1	2017-04-26	Pacific Ocean: Near Hawaii	kidney	Lepidochyelys olivacea	Northern Arizona University	QNQW00000000	SRR7469193
STNAU-25339-1	25339-1	2017-06-02	Pacific Ocean: Near Hawaii	kidney	Lepidochyelys olivacea	Northern Arizona University	QNQV00000000	SRR7469196
STNAU-25433-1	25433-1	2017-06-14	Pacific Ocean: Near Hawaii	kidney	Lepidochyelys olivacea	Northern Arizona University	QMAA00000000	SRR7469205
STNAU-7583-6	7583-6	1988-01-13	USA:California	liver	Pelecanus occidentalis	Northern Arizona University	QMAD00000000	SRR7469198
STNAU-15332-3	15332-3	2000-09-28	Pacific Ocean: Near Hawaii	spleen	Nycticorax nycticorax	Northern Arizona University	QMAC00000000	SRR7469197

STNAU-26172-1	26172-1	2015-02-25	USA:Maryland	colon	Malaclemys terrapin	Northern Arizona University	QLZZ00000000	SRR7469203
STNAU-26639-1	26639-1	2015-07-23	USA:Pennsylvania	intestine	Terrepene carolina	Northern Arizona University	QLZY00000000	SRR7469202

Table S2. GenBank accessions for strains included in Figure 3.			
**Genome/Assembly Accession**	**Serovar**	**Strain**	**SRA Accession (for strains in Fig 4B)**
QMAC00000000	Typhimurium	15332-3	SRR7469197
QNQY00000000	Typhimurium	21812-1	SRR7469201
QNQX00000000	Typhimurium	25231-1	SRR7469194
QNQW00000000	Typhimurium	25234-1	SRR7469193
QNQV00000000	Typhimurium	25339-1	SRR7469196
QMAA00000000	Typhimurium	25433-1	SRR7469205
QLZY00000000	Typhimurium	26639-1	SRR7469202
QLZV00000000	Typhimurium	337781	SRR7469206
QLZU00000000	Typhimurium	337782	SRR7469195
QLZX00000000	Typhimurium	LOL16001	SRR7469200
QLZW00000000	Typhimurium	LOL16002	SRR7469199
GCA_001566115.1	Typhimurium	DT104_V21	ERR026017
GCA_001216485.1	Typhimurium	DT104_V41	ERR026032
GCA_001221905.1	Typhimurium	DT104_V35	ERR026039
GCA_001217025.1	Typhimurium	DT104_H69	ERR026096
GCA_001215105.1	Typhimurium	DT104_H67	ERR026105
GCA_001219505.1	Typhimurium	DT104_V42	ERR026146
GCA_001219665.1	Typhimurium	DT104_H31	ERR028474
GCA_002106195.1	4,5,12:i	NY_FSL_S10_1237	SRR1023846
GCA_002030775.1	Typhimurium	var_5_BCW_2247	SRR1060534
GCA_002033135.1	Typhimurium	var_5_BCW_2036	SRR1060580
GCA_002106175.1	Typhimurium	NY_FSL_S10_1269	SRR1106216
GCA_002063725.1	Typhimurium	BCW_2793	SRR1106414
GCA_002031675.1	Typhimurium	var_5_BCW_2101	SRR1106601
GCA_002061625.1	Copenhagen	BCW_3397	SRR1122501
GCA_002034225.1	Typhimurium	NVSL_7095	SRR1840574

GCA_002035635.1	Typhimurium	var_5_BCW_2127	SRR1840612
GCA_002066815.1	Typhimurium	6623	SRR1840660
GCA_001272465.1	Typhimurium	var_5_CVM_N51269	SRR2567162
GCA_002091015.1	Typhimurium	R8_8132_R1	SRR3097410
GCA_001478285.1	Typhimurium	var_5_CVM_N37950	SRR3665303
GCA_002074385.1	Typhimurium	LT2_KO_STM0401	SRR5288750
GCA_002289275.1	Typhimurium	LT2_KO_STM3712	SRR5483897
GCA_002265285.1	Typhimurium	Q1	SRR5860302
GCA_002264665.1	Typhimurium	D15_043619	SRR5985922
GCA_001595305.1	Typhimurium	CFSAN033851	SRR6684719
GCA_001588545.1	Typhimurium	CFSAN033868	SRR6690141
GCA_001595105.1	Typhimurium	CFSAN033863	SRR6690143
GCA_001595355.1	Typhimurium	CFSAN033848	SRR6690144
GCA_001588125.1	Typhimurium	CFSAN033913	SRR6690152
GCA_001594935.1	Typhimurium	CFSAN033872	SRR6690157
GCA_001595065.1	Typhimurium	CFSAN033865	SRR6690662
GCA_001595155.1	Typhimurium	CFSAN033860	SRR6690671
GCA_001588105.1	Typhimurium	CFSAN033914	SRR6690673
GCA_001595265.1	Typhimurium	CFSAN033853	SRR6690684
GCA_001587845.1	Typhimurium	CFSAN033933	SRR6690685
GCA_001587925.1	Typhimurium	CFSAN033927	SRR6690687
GCA_001594995.1	Typhimurium	CFSAN033869	SRR6690688
GCA_001594535.1	Typhimurium	CFSAN033912	SRR6690699
GCA_001587755.1	Typhimurium	CFSAN033938	SRR6690702
GCA_001595035.1	Typhimurium	CFSAN033866	SRR6690705
GCA_001588495.1	Typhimurium	CFSAN033885	SRR6690707
GCA_001588045.1	Typhimurium	CFSAN033918	SRR6690720
GCA_001595315.1	Typhimurium	CFSAN033850	SRR6690726
GCA_001595235.1	Typhimurium	CFSAN033854	SRR6690730
GCA_001594525.1	Typhimurium	CFSAN033916	SRR6690732
GCA_001595345.1	Typhimurium	CFSAN033849	SRR6690744
GCA_001955315.1*	Typhimurium	R8_9832_R1	SRR3097430
GCA_002074445.1*	Typhimurium	LT2 KO STM2988	SRR5288745

GCA_001594915.1*	Typhimurium	CFSAN033873	SRR6690704
GCA_000022165.1	Typhimurium	14028S	
GCA_000027025.1	Typhimurium	D23580	
GCA_000170255.1	4,5,12:i	CVM23701	
GCA_000187785.2	Typhimurium	TN061786	
GCA_000188735.1	Typhimurium	ST4_74	
GCA_000213635.1	Typhimurium	UK_1	
GCA_000252875.1	Typhimurium	798	
GCA_000283735.1	Typhimurium	T000240	
GCA_000292755.1	Typhimurium	ST1660_06	
GCA_000312745.2	Typhimurium	STm6	
GCA_000312765.2	Typhimurium	STm11	
GCA_000312785.2	Typhimurium	STm12	
GCA_000312805.2	Typhimurium	STm5	
GCA_000312825.2	Typhimurium	STm1	
GCA_000312845.2	Typhimurium	STm8	
GCA_000312865.2	Typhimurium	STm3	
GCA_000312885.2	Typhimurium	STm4	
GCA_000312905.2	Typhimurium	STm10	
GCA_000314915.2	Typhimurium	STm2	
GCA_000319795.2	Typhimurium	STm9	
GCA_000335915.1	4,5,12:i	08_1700	
GCA_000335935.1	4,5,12:i	08_1739	
GCA_000336195.1	Typhimurium	LT2_4_delta_ramA_kan	
GCA_000336215.1	Typhimurium	LT2_4	
GCA_000380325.1	Typhimurium	U288	
GCA_000430145.3	Typhimurium	var_5_CFSAN001921_11NY04CB01_S	
GCA_000430165.1	4,5,12:i	08_1736	
GCA_000444835.2	Typhimurium	STm7	
GCA_000465195.1	Typhimurium	ST1489	
GCA_000468255.1	Typhimurium	ST4848	
GCA_000474335.1	Typhimurium	CDC_2009K1288_2009K_1288	
GCA_000474395.1	Typhimurium	35423	

GCA_000474495.1	Typhimurium	CDC_2009K1277_2009K_1277	
GCA_000474555.1	Typhimurium	34502	
GCA_000474575.1	Typhimurium	36618	
GCA_000486125.1	Typhimurium	ST4581	
GCA_000486345.2	Typhimurium	SARA13	
GCA_000493535.2	Typhimurium	DT2	
GCA_000493675.1	Typhimurium	DT104	
GCA_000495135.2	Typhimurium	L945	
GCA_000495175.2	Typhimurium	L796	
GCA_000495215.2	Typhimurium	L927	
GCA_000495235.1	Typhimurium	L847	
GCA_000505085.1	Typhimurium	var_Copenhagen_0084	
GCA_000614485.1	Typhimurium	JCM_1652	
GCA_000636135.1	Typhimurium	138736	
GCA_000648415.2	Typhimurium	95799	
GCA_000648435.2	Typhimurium	98346	
GCA_000648455.2	Typhimurium	116045	
GCA_000648475.2	Typhimurium	104772	
GCA_000648495.2	Typhimurium	108402	
GCA_000715155.2	Typhimurium	VNP20009	
GCA_000743055.1	Typhimurium	ATCC_13311	
GCA_000784215.1	Typhimurium	S7	
GCA_000784225.1	Typhimurium	S10	
GCA_000784245.1	Typhimurium	S15	
GCA_000784295.1	Typhimurium	S42	
GCA_000784315.1	Typhimurium	S46	
GCA_000786025.1	Typhimurium	86_0368	
GCA_000828595.1	Typhimurium	L_3553	
GCA_000941015.2	Typhimurium	USDA_ARS_USMARC_1899	
GCA_000973645.1	Typhimurium	CDC_2011K_0870	
GCA_000973885.1	Typhimurium	SALH_394_2	
GCA_000973915.1	Typhimurium	ABB1162_2	
GCA_000973985.1	Typhimurium	ABBSB1189_1	

GCA_000974215.1	Typhimurium	SALF_297_3	
GCA_001026705.1	Typhimurium	smonpas0031082	
GCA_001027585.1	Typhimurium	smonpas008251_3	
GCA_001027595.1	Typhimurium	smonpas008251_5	
GCA_001027615.1	Typhimurium	smonpas001102_2	
GCA_001034525.1	Typhimurium	smonpas004260_1	
GCA_001034545.1	Typhimurium	smonpas003168_4	
GCA_001034565.1	Typhimurium	smonpas001102_13	
GCA_001034585.1	Typhimurium	smonpas003168_5	
GCA_001034595.1	Typhimurium	smonpas004260_10	
GCA_001034605.1	Typhimurium	smonpas003168_1	
GCA_001034615.1	Typhimurium	smonpas004260_8	
GCA_001038085.1	Typhimurium	smonpas007416_3	
GCA_001038095.1	Typhimurium	smonpas007416_4	
GCA_001038105.1	Typhimurium	smonpas009854	
GCA_001038115.1	Typhimurium	smonpas001102_11	
GCA_001038165.1	Typhimurium	smonpas006644_5	
GCA_001042425.1	Typhimurium	smonpas0023280	
GCA_001085145.1	Typhimurium	DT104_CH33	
GCA_001085685.1	Typhimurium	DT104_CH32	
GCA_001091445.1	Typhimurium	DT104_CH1	
GCA_001093545.1	Typhimurium	DT104_CH17	
GCA_001096565.1	Typhimurium	DT104_CH5	
GCA_001097045.1	Typhimurium	DT104_CH51	
GCA_001102665.1	Typhimurium	DT104_CH6	
GCA_001110745.1	Typhimurium	DT104_CH26	
GCA_001111905.1	Typhimurium	DT104_CH25	
GCA_001116925.1	Typhimurium	DT104_CH19	
GCA_001119985.1	Typhimurium	DT104_CH46	
GCA_001120065.1	Typhimurium	DT104_CH16	
GCA_001125725.1	Typhimurium	DT104_CH14	
GCA_001127885.1	Typhimurium	DT104_CH28	
GCA_001129385.1	Typhimurium	DT104_CH13	

GCA_001130025.1	Typhimurium	DT104_CH50	
GCA_001130925.1	Typhimurium	DT104_CH34	
GCA_001131525.1	Typhimurium	DT104_CH40	
GCA_001135785.1	Typhimurium	DT104_CH20	
GCA_001137885.1	Typhimurium	DT104_CH21	
GCA_001139325.1	Typhimurium	DT104_CH47	
GCA_001146465.1	Typhimurium	DT104_CH31	
GCA_001153005.1	Typhimurium	DT104_CH41	
GCA_001154585.1	Typhimurium	DT104_CH4	
GCA_001155485.1	Typhimurium	DT104_CH30	
GCA_001159405.1	Typhimurium	DT104_CH45	
GCA_001161925.1	Typhimurium	DT104_CH22	
GCA_001162405.1	Typhimurium	DT104_CH10	
GCA_001164125.1	Typhimurium	DT104_CH9	
GCA_001164765.1	Typhimurium	DT104_CH49	
GCA_001172145.1	Typhimurium	DT104_CH18	
GCA_001172545.1	Typhimurium	DT104_CH35	
GCA_001187375.1	Typhimurium	Sal368	
GCA_001214325.1	Typhimurium	DT104_V40	
GCA_001214365.1	Typhimurium	DT104_H106	
GCA_001214425.1	Typhimurium	DT104_SPAINg_H161	
GCA_001214485.1	Typhimurium	DT104_SPAINc_H165	
GCA_001214545.1	Typhimurium	DT104_H92	
GCA_001214705.1	Typhimurium	DT104_H131	
GCA_001214765.1	Typhimurium	DT104_V74	
GCA_001214865.1	Typhimurium	DT104_EWV9	
GCA_001215365.1	Typhimurium	DT104_H141	
GCA_001215405.1	Typhimurium	DT104_H21	
GCA_001215505.1	Typhimurium	DT104_V73	
GCA_001215525.1	Typhimurium	DT104_V20	
GCA_001215545.1	Typhimurium	DT104_H04	
GCA_001215585.1	Typhimurium	DT104_H77	
GCA_001215665.1	Typhimurium	DT104_V85	

GCA_001215685.1	Typhimurium	DT104_H02	
GCA_001215725.1	Typhimurium	DT104_V65	
GCA_001215785.1	Typhimurium	DT104_V19	
GCA_001215865.1	Typhimurium	DT104_H121	
GCA_001215885.1	Typhimurium	DT104_V15	
GCA_001215925.1	Typhimurium	DT104_SPAINa_H163	
GCA_001216085.1	Typhimurium	DT104_V107	
GCA_001216125.1	Typhimurium	DT104_THAILAND_H157	
GCA_001216345.1	Typhimurium	DT104_H125	
GCA_001216385.1	Typhimurium	DT104_H139	
GCA_001216425.1	Typhimurium	DT104_V39	
GCA_001216465.1	Typhimurium	DT104_SPAINh_H166	
GCA_001216545.1	Typhimurium	DT104_H44	
GCA_001216565.1	Typhimurium	DT104_EWH8	
GCA_001216585.1	Typhimurium	DT104_H36	
GCA_001216605.1	Typhimurium	DT104_H45	
GCA_001216645.1	Typhimurium	DT104_H01	
GCA_001216705.1	Typhimurium	DT104_V26	
GCA_001216765.1	Typhimurium	DT104_SOUTH_AFRICA_H180	
GCA_001216785.1	Typhimurium	DT104_V96	
GCA_001216845.1	Typhimurium	DT104_V48	
GCA_001216985.1	Typhimurium	DT104_H62	
GCA_001217045.1	Typhimurium	DT104_V61	
GCA_001217085.1	Typhimurium	DT104_V59	
GCA_001217205.1	Typhimurium	DT104_V81	
GCA_001217265.1	Typhimurium	DT104_V44	
GCA_001217425.1	Typhimurium	DT104_H43	
GCA_001217445.1	Typhimurium	DT104_V03	
GCA_001217545.1	Typhimurium	DT104_H151	
GCA_001217585.1	Typhimurium	DT104_H154	
GCA_001217605.1	Typhimurium	DT104_V25	
GCA_001217625.1	Typhimurium	DT104_EWV2	
GCA_001217645.1	Typhimurium	DT104_V67	

GCA_001217665.1	Typhimurium	DT104_EWV11	
GCA_001217705.1	Typhimurium	DT104_MOROCCO_H158	
GCA_001217765.1	Typhimurium	DT104_H72	
GCA_001217785.1	Typhimurium	DT104_H142	
GCA_001217825.1	Typhimurium	DT104_V31	
GCA_001217845.1	Typhimurium	DT104_V91	
GCA_001217865.1	Typhimurium	DT104_H112	
GCA_001217885.1	Typhimurium	DT104_EWV4	
GCA_001217905.1	Typhimurium	DT104_H16	
GCA_001217925.1	Typhimurium	DT104_EWH7	
GCA_001217945.1	Typhimurium	DT104_H145	
GCA_001217965.1	Typhimurium	DT104_EWH6	
GCA_001218065.1	Typhimurium	DT104_H97	
GCA_001218105.1	Typhimurium	DT104_H39	
GCA_001218125.1	Typhimurium	DT104_H73	
GCA_001218225.1	Typhimurium	DT104_H143	
GCA_001218245.1	Typhimurium	DT104_EWH12	
GCA_001218265.1	Typhimurium	DT104_MALTAd_H177	
GCA_001218285.1	Typhimurium	DT104_H48	
GCA_001218305.1	Typhimurium	DT104_H86	
GCA_001218325.1	Typhimurium	DT104_H30	
GCA_001218345.1	Typhimurium	DT104_H120	
GCA_001218365.1	Typhimurium	DT104_H129	
GCA_001218385.1	Typhimurium	DT104_V45	
GCA_001218405.1	Typhimurium	DT104_H95	
GCA_001218425.1	Typhimurium	DT104_H136	
GCA_001218445.1	Typhimurium	DT104_EWH9	
GCA_001218465.1	Typhimurium	DT104_H103	
GCA_001218505.1	Typhimurium	DT104_V54	
GCA_001218545.1	Typhimurium	DT104_H28	
GCA_001218565.1	Typhimurium	DT104_V86	
GCA_001218585.1	Typhimurium	DT104_H35	
GCA_001218605.1	Typhimurium	DT104_V22	

GCA_001218625.1	Typhimurium	DT104_H83	
GCA_001218685.1	Typhimurium	DT104_V47	
GCA_001218725.1	Typhimurium	DT104_V60	
GCA_001218745.1	Typhimurium	DT104_H11	
GCA_001218765.1	Typhimurium	DT104_V52	
GCA_001218785.1	Typhimurium	DT104_H140	
GCA_001218805.1	Typhimurium	DT104_H51	
GCA_001218845.1	Typhimurium	DT104_V23	
GCA_001218865.1	Typhimurium	DT104_H59	
GCA_001218925.1	Typhimurium	DT104_H18	
GCA_001218965.1	Typhimurium	DT104_V11	
GCA_001218985.1	Typhimurium	DT104_V125	
GCA_001219025.1	Typhimurium	DT104_H124	
GCA_001219045.1	Typhimurium	DT104_H08	
GCA_001219065.1	Typhimurium	DT104_H56	
GCA_001219105.1	Typhimurium	DT104_H57	
GCA_001219125.1	Typhimurium	DT104_H63	
GCA_001219145.1	Typhimurium	DT104_H74	
GCA_001219165.1	Typhimurium	DT104_H55	
GCA_001219225.1	Typhimurium	DT104_V53	
GCA_001219265.1	Typhimurium	DT104_V32	
GCA_001219285.1	Typhimurium	DT104_H27	
GCA_001219305.1	Typhimurium	DT104_H17	
GCA_001219365.1	Typhimurium	DT104_V76	
GCA_001219405.1	Typhimurium	DT104_V55	
GCA_001219425.1	Typhimurium	DT104_H07	
GCA_001219445.1	Typhimurium	DT104_UNITED_STATES_H172	
GCA_001219465.1	Typhimurium	DT104_V112	
GCA_001219485.1	Typhimurium	DT104_H06	
GCA_001219525.1	Typhimurium	DT104_EWV8	
GCA_001219545.1	Typhimurium	DT104_V63	
GCA_001219565.1	Typhimurium	DT104_V94	
GCA_001219645.1	Typhimurium	DT104_V56	

GCA_001219705.1	Typhimurium	DT104_V103	
GCA_001219725.1	Typhimurium	DT104_H130	
GCA_001219805.1	Typhimurium	DT104_SRI_LANKA_H173	
GCA_001219825.1	Typhimurium	DT104_FAR_EAS_H156	
GCA_001219845.1	Typhimurium	DT104_V51	
GCA_001219865.1	Typhimurium	DT104_H26	
GCA_001219885.1	Typhimurium	DT104_H133	
GCA_001219905.1	Typhimurium	DT104_V122	
GCA_001219925.1	Typhimurium	DT104_V01	
GCA_001219945.1	Typhimurium	DT104_H38	
GCA_001219965.1	Typhimurium	DT104_V46	
GCA_001220025.1	Typhimurium	DT104_H66	
GCA_001220065.1	Typhimurium	DT104_V50	
GCA_001220105.1	Typhimurium	DT104_H102	
GCA_001220125.1	Typhimurium	DT104_V111	
GCA_001220205.1	Typhimurium	DT104_V97	
GCA_001220225.1	Typhimurium	DT104_EWH1	
GCA_001220245.1	Typhimurium	DT104_H144	
GCA_001220265.1	Typhimurium	DT104_V38	
GCA_001220305.1	Typhimurium	DT104_H135	
GCA_001220345.1	Typhimurium	DT104_V70	
GCA_001220365.1	Typhimurium	DT104_EWH2	
GCA_001220385.1	Typhimurium	DT104_H24	
GCA_001220405.1	Typhimurium	DT104_EWV1	
GCA_001220445.1	Typhimurium	DT104_V118	
GCA_001220465.1	Typhimurium	DT104_TURKEYa_H178	
GCA_001220505.1	Typhimurium	DT104_SPAINf_H160	
GCA_001220525.1	Typhimurium	DT104_H71	
GCA_001220585.1	Typhimurium	DT104_V57	
GCA_001220625.1	Typhimurium	DT104_H79	
GCA_001220645.1	Typhimurium	DT104_SPAINb_H164	
GCA_001220665.1	Typhimurium	DT104_V43	
GCA_001220685.1	Typhimurium	DT104_H128	

GCA_001220705.1	Typhimurium	DT104_V09	
GCA_001220725.1	Typhimurium	DT104_V114	
GCA_001220785.1	Typhimurium	DT104_H150	
GCA_001220865.1	Typhimurium	DT104_H94	
GCA_001220885.1	Typhimurium	DT104_SPAINi_H184	
GCA_001220905.1	Typhimurium	DT104_H138	
GCA_001220925.1	Typhimurium	DT104_H110	
GCA_001220945.1	Typhimurium	DT104_V119	
GCA_001220965.1	Typhimurium	DT104_V66	
GCA_001220985.1	Typhimurium	DT104_V02	
GCA_001221005.1	Typhimurium	DT104_V68	
GCA_001221025.1	Typhimurium	DT104_H68	
GCA_001221045.1	Typhimurium	DT104_H40	
GCA_001221065.1	Typhimurium	DT104_H88	
GCA_001221105.1	Typhimurium	DT104_V121	
GCA_001221125.1	Typhimurium	DT104_H64	
GCA_001221225.1	Typhimurium	DT104_FRANCEa_H168	
GCA_001221245.1	Typhimurium	DT104_H52	
GCA_001221265.1	Typhimurium	DT104_H134	
GCA_001221305.1	Typhimurium	DT104_H61	
GCA_001221345.1	Typhimurium	DT104_H107	
GCA_001221365.1	Typhimurium	DT104_H153	
GCA_001221385.1	Typhimurium	DT104_EWH5	
GCA_001221405.1	Typhimurium	DT104_H149	
GCA_001221445.1	Typhimurium	DT104_H90	
GCA_001221485.1	Typhimurium	DT104_H03	
GCA_001221505.1	Typhimurium	DT104_V124	
GCA_001221545.1	Typhimurium	DT104_V100	
GCA_001221585.1	Typhimurium	DT104_H20	
GCA_001221605.1	Typhimurium	DT104_H93	
GCA_001221625.1	Typhimurium	DT104_H12	
GCA_001221645.1	Typhimurium	DT104_H117	
GCA_001221665.1	Typhimurium	DT104_EWV7	

GCA_001221685.1	Typhimurium	DT104_V84	
GCA_001221705.1	Typhimurium	DT104_V113	
GCA_001221725.1	Typhimurium	DT104_V17	
GCA_001221765.1	Typhimurium	DT104_H75	
GCA_001221785.1	Typhimurium	DT104_V80	
GCA_001221805.1	Typhimurium	DT104_H46	
GCA_001221885.1	Typhimurium	DT104_EWV6	
GCA_001221925.1	Typhimurium	DT104_H104	
GCA_001221985.1	Typhimurium	DT104_SPAINd_H167	
GCA_001222005.1	Typhimurium	DT104_SPAINe_H159	
GCA_001222025.1	Typhimurium	DT104_H137	
GCA_001222065.1	Typhimurium	DT104_H29	
GCA_001222085.1	Typhimurium	DT104_H98	
GCA_001222125.1	Typhimurium	DT104_H22	
GCA_001222145.1	Typhimurium	DT104_V49	
GCA_001222165.1	Typhimurium	DT104_MALTAc_H176	
GCA_001222185.1	Typhimurium	DT104_H49	
GCA_001222345.1	Typhimurium	DT104_H41	
GCA_001222405.1	Typhimurium	DT104_CYPRUS_H182	
GCA_001222465.1	Typhimurium	DT104_H05	
GCA_001222485.1	Typhimurium	DT104_H80	
GCA_001222505.1	Typhimurium	DT104_H32	
GCA_001222565.1	Typhimurium	DT104_H148	
GCA_001222605.1	Typhimurium	DT104_V83	
GCA_001222625.1	Typhimurium	DT104_H50	
GCA_001222645.1	Typhimurium	DT104_V27	
GCA_001222705.1	Typhimurium	DT104_H78	
GCA_001222725.1	Typhimurium	DT104_V99	
GCA_001222785.1	Typhimurium	DT104_V104	
GCA_001222825.1	Typhimurium	DT104_H147	
GCA_001222865.1	Typhimurium	DT104_H13	
GCA_001222885.1	Typhimurium	DT104_V29	
GCA_001222905.1	Typhimurium	DT104_H60	

GCA_001222965.1	Typhimurium	DT104_GREECE_H170	
GCA_001223025.1	Typhimurium	DT104_V62	
GCA_001223045.1	Typhimurium	DT104_H96	
GCA_001223085.1	Typhimurium	DT104_V78	
GCA_001223105.1	Typhimurium	DT104_H54	
GCA_001223125.1	Typhimurium	DT104_H123	
GCA_001223165.1	Typhimurium	DT104_H118	
GCA_001223185.1	Typhimurium	DT104_V36	
GCA_001223225.1	Typhimurium	DT104_V05	
GCA_001223245.1	Typhimurium	DT104_H47	
GCA_001223305.1	Typhimurium	DT104_H14	
GCA_001223325.1	Typhimurium	DT104_H82	
GCA_001223345.1	Typhimurium	DT104_ITALY_H171	
GCA_001223365.1	Typhimurium	DT104_V90	
GCA_001223445.1	Typhimurium	DT104_H10	
GCA_001223465.1	Typhimurium	DT104_H70	
GCA_001223505.1	Typhimurium	DT104_TURKEYb_H179	
GCA_001223545.1	Typhimurium	DT104_V117	
GCA_001240475.1	Typhimurium	var_5_CVM_N43469	
GCA_001240785.1	Typhimurium	var_5_CVM_N43822	
GCA_001240805.1	Typhimurium	var_5_CVM_N43824	
GCA_001240825.1	Typhimurium	var_5_CVM_N43823	
GCA_001240925.1	Typhimurium	CVM_N43829	
GCA_001240935.1	Typhimurium	var_5_CVM_N43830	
GCA_001240965.1	Typhimurium	var_5_CVM_N43831	
GCA_001241165.1	Typhimurium	var_5_CVM_N44697	
GCA_001241425.1	Typhimurium	var_5_CVM_N44709	
GCA_001241505.1	Typhimurium	var_5_CVM_N44712	
GCA_001241515.1	Typhimurium	var_5_CVM_N44713	
GCA_001241585.1	Typhimurium	var_5_CVM_N44715	
GCA_001241605.1	Typhimurium	var_5_CVM_N44716	
GCA_001241895.1	Typhimurium	var_5_CVM_N45403	
GCA_001241905.1	Typhimurium	var_5_CVM_N45404	

GCA_001241945.1	Typhimurium	var_5_CVM_N45405	
GCA_001241975.1	Typhimurium	var_5_CVM_N45408	
GCA_001242275.1	Typhimurium	var_5_CVM_N45935	
GCA_001242345.1	Typhimurium	var_5_CVM_N45936	
GCA_001242395.1	Typhimurium	var_5_CVM_N45938	
GCA_001242565.1	Typhimurium	var_5_CVM_N45950	
GCA_001243095.1	Typhimurium	var_5_CVM_N46824	
GCA_001243105.1	Typhimurium	var_5_CVM_N46825	
GCA_001243255.1	Typhimurium	var_5_CVM_N46834	
GCA_001243395.1	Typhimurium	var_5_CVM_N46844	
GCA_001243425.1	Typhimurium	var_5_CVM_N46845	
GCA_001243555.1	Typhimurium	var_5_CVM_N46852	
GCA_001243815.1	Typhimurium	var_5_CVM_N47715	
GCA_001244105.1	Typhimurium	var_5_CVM_N47728	
GCA_001244255.1	Typhimurium	var_5_CVM_N48676	
GCA_001244285.1	Typhimurium	var_5_CVM_N48677	
GCA_001244335.1	Typhimurium	var_5_CVM_N48679	
GCA_001244385.1	Typhimurium	var_5_CVM_N48680	
GCA_001244695.1	Typhimurium	var_5_CVM_N48690	
GCA_001244705.1	Typhimurium	var_5_CVM_N48689	
GCA_001245125.1	Typhimurium	var_5_CVM_N48708	
GCA_001245255.1	4,5,12:i	CVM_N50420	
GCA_001245415.1	Typhimurium	var_5_CVM_N50425	
GCA_001245495.1	Typhimurium	var_5_CVM_N50430	
GCA_001245535.1	Typhimurium	var_5_CVM_N50433	
GCA_001245645.1	Typhimurium	CVM_N50438	
GCA_001245735.1	4,5,12:i	CVM_N50442	
GCA_001245765.1	4,5,12:i	CVM_N50445	
GCA_001246225.1	Typhimurium	CVM_N51254	
GCA_001246265.1	Typhimurium	var_5_CVM_N51255	
GCA_001246565.1	Typhimurium	var_5_CVM_N51268	
GCA_001246625.1	Typhimurium	var_5_CVM_N51272	
GCA_001246695.1	Typhimurium	var_5_CVM_N51275	

GCA_001246705.1	Typhimurium	CVM_N51276	
GCA_001246875.1	Typhimurium	CVM_N51283	
GCA_001246945.1	4,5,12:i	CVM_N51287	
GCA_001247185.1	Typhimurium	CVM_N51299	
GCA_001247205.1	Typhimurium	var_5_CVM_N51300	
GCA_001247265.1	Typhimurium	var_5_CVM_N51301	
GCA_001247275.1	Typhimurium	var_5_CVM_N51303	
GCA_001271505.1	Typhimurium	var_5_CVM_N45390	
GCA_001271565.1	Typhimurium	var_5_CVM_N45391	
GCA_001271645.1	Typhimurium	CVM_N46810	
GCA_001271715.1	Typhimurium	var_5_CVM_N46828	
GCA_001271745.1	Typhimurium	var_5_CVM_N46841	
GCA_001271795.1	Typhimurium	var_5_CVM_N46843	
GCA_001271825.1	Typhimurium	var_5_CVM_N46861	
GCA_001271835.1	Typhimurium	var_5_CVM_N46848	
GCA_001271965.1	Typhimurium	CVM_N43825	
GCA_001272035.1	Typhimurium	var_5_CVM_N45410	
GCA_001272045.1	Typhimurium	var_5_CVM_N44717	
GCA_001272095.1	Typhimurium	var_5_CVM_N46826	
GCA_001272115.1	Typhimurium	var_5_CVM_N46840	
GCA_001272415.1	Typhimurium	CVM_N51295	
GCA_001272475.1	Typhimurium	var_5_CVM_N51296	
GCA_001272535.1	Typhimurium	CVM_N51314	
GCA_001293505.1	Typhimurium	33676	
GCA_001295155.1	Typhimurium	StVIM	
GCA_001295385.1	Typhimurium	ST4024	
GCA_001295405.1	Typhimurium	ST111849	
GCA_001295415.1	Typhimurium	ST2850	
GCA_001295465.1	Typhimurium	ST2286	
GCA_001295485.1	Typhimurium	ST486	
GCA_001295525.1	Typhimurium	ST3858	
GCA_001295545.1	Typhimurium	ST8493	
GCA_001295555.1	Typhimurium	ST6988	

GCA_001295585.1	Typhimurium	ST372	
GCA_001295625.1	Typhimurium	ST2533	
GCA_001295635.1	Typhimurium	ST4650	
GCA_001295665.1	Typhimurium	ST2143	
GCA_001295675.1	Typhimurium	ST4329	
GCA_001295705.1	Typhimurium	ST4038	
GCA_001295725.1	Typhimurium	ST3363	
GCA_001295745.1	Typhimurium	ST2287	
GCA_001326155.1	Typhimurium	DT104_EWV10	
GCA_001326175.1	Typhimurium	DT104_EWH10	
GCA_001326195.1	Typhimurium	DT104_EWH3	
GCA_001326215.1	Typhimurium	DT104_EWH4	
GCA_001326235.1	Typhimurium	DT104_EWH11	
GCA_001326255.1	Typhimurium	DT104_H34	
GCA_001326275.1	Typhimurium	DT104_V16	
GCA_001350035.1	Typhimurium	DT104_CH15	
GCA_001350055.1	Typhimurium	DT104_CH24	
GCA_001350075.1	Typhimurium	DT104_CH12	
GCA_001350095.1	Typhimurium	DT104_CH36	
GCA_001350115.1	Typhimurium	DT104_CH2	
GCA_001350135.1	Typhimurium	DT104_CH42	
GCA_001350155.1	Typhimurium	DT104_CH11	
GCA_001350175.1	Typhimurium	DT104_CH37	
GCA_001350195.1	Typhimurium	DT104_CH3	
GCA_001350215.1	Typhimurium	DT104_CH23	
GCA_001350235.1	Typhimurium	DT104_CH38	
GCA_001350255.1	Typhimurium	DT104_CH29	
GCA_001350275.1	Typhimurium	DT104_CH39	
GCA_001457365.1	Typhimurium	ST221_31B	
GCA_001466375.1	Typhimurium	2013LSAL04524	
GCA_001477735.1	Typhimurium	CVM_N29313	
GCA_001477745.1	Typhimurium	var_5_CVM_N29315	
GCA_001477805.1	Typhimurium	CVM_N29338	

GCA_001477855.1	Typhimurium	var_5_CVM_N29350	
GCA_001477895.1	Typhimurium	CVM_N29323	
GCA_001478045.1	Typhimurium	var_5_CVM_N30693	
GCA_001478065.1	Typhimurium	CVM_N30696	
GCA_001478125.1	Typhimurium	CVM_N31395	
GCA_001478325.1	Typhimurium	var_5_CVM_N38858	
GCA_001478425.1	Typhimurium	CVM_N38925	
GCA_001478445.1	Typhimurium	var_5_CVM_N38944	
GCA_001478625.1	Typhimurium	var_5_CVM_N29343	
GCA_001478885.1	Typhimurium	var_5_CVM_N30655	
GCA_001479065.1	Typhimurium	var_5_CVM_N31392	
GCA_001479125.1	Typhimurium	CVM_N31409	
GCA_001479155.1	Typhimurium	var_5_CVM_N31411	
GCA_001479335.1	Typhimurium	CVM_N32048	
GCA_001479365.1	Typhimurium	var_5_CVM_N32051	
GCA_001479395.1	Typhimurium	var_5_CVM_N32056	
GCA_001479695.1	Typhimurium	var_5_CVM_N38234	
GCA_001479815.1	4,5,12:i	CVM_N38855	
GCA_001479925.1	Typhimurium	var_5_CVM_N38873	
GCA_001480025.1	Typhimurium	var_5_CVM_N38903	
GCA_001480055.1	Typhimurium	CVM_N38917	
GCA_001480085.1	Typhimurium	CVM_N38921	
GCA_001480105.1	Typhimurium	var_5_CVM_N38943	
GCA_001480115.1	Typhimurium	CVM_N38929	
GCA_001480145.1	Typhimurium	var_5_CVM_N40368	
GCA_001480205.1	Typhimurium	var_5_CVM_N40379	
GCA_001480225.1	Typhimurium	var_5_CVM_N40384	
GCA_001480265.1	Typhimurium	var_5_CVM_N40416	
GCA_001480285.1	Typhimurium	var_5_CVM_N40935	
GCA_001480365.1	4,5,12:i	CVM_N41895	
GCA_001480385.1	Typhimurium	var_5_CVM_N41904	
GCA_001480485.1	Typhimurium	CVM_N42450	
GCA_001480775.1	Typhimurium	var_5_CVM_N37941	

GCA_001480795.1	Typhimurium	var_5_CVM_N38229	
GCA_001480855.1	Typhimurium	CVM_N38914	
GCA_001480975.1	4,5,12:i	CVM_N40939	
GCA_001480985.1	Typhimurium	var_5_CVM_N41906	
GCA_001481075.1	Typhimurium	CVM_N42467	
GCA_001481175.1	Typhimurium	var_5_CVM_N42492	
GCA_001481235.1	Typhimurium	var_5_CVM_N42518	
GCA_001540845.1	Typhimurium	SO4698_09	
GCA_001559855.1	Typhimurium	MHM112	
GCA_001565935.1	Typhimurium	DT104_EWV5	
GCA_001565955.1	Typhimurium	DT104_H89	
GCA_001565975.1	Typhimurium	DT104_H105	
GCA_001565995.1	Typhimurium	DT104_EWV3	
GCA_001566035.1	Typhimurium	DT104_H132	
GCA_001566055.1	Typhimurium	DT104_V13	
GCA_001566095.1	Typhimurium	DT104_V79	
GCA_001566155.1	Typhimurium	DT104_H127	
GCA_001566175.1	Typhimurium	DT104_H15	
GCA_001576255.1	Typhimurium	YU15	
GCA_001576275.1	Typhimurium	SO2	
GCA_001577505.1	Typhimurium	SO3	
GCA_001587055.1	Typhimurium	SL1344RX_Chromosome	
GCA_001587765.1	Typhimurium	CFSAN033935	
GCA_001587775.1	Typhimurium	CFSAN033934	
GCA_001587785.1	Typhimurium	CFSAN033937	
GCA_001587835.1	Typhimurium	CFSAN033892	
GCA_001587855.1	Typhimurium	CFSAN033932	
GCA_001587885.1	Typhimurium	CFSAN033930	
GCA_001587915.1	Typhimurium	CFSAN033929	
GCA_001587945.1	Typhimurium	CFSAN033926	
GCA_001587965.1	Typhimurium	CFSAN033925	
GCA_001587995.1	Typhimurium	CFSAN033922	
GCA_001588015.1	Typhimurium	CFSAN033921	

GCA_001588025.1	Typhimurium	CFSAN033919	
GCA_001588075.1	Typhimurium	CFSAN033917	
GCA_001588095.1	Typhimurium	CFSAN033915	
GCA_001588155.1	Typhimurium	CFSAN033911	
GCA_001588165.1	Typhimurium	CFSAN033910	
GCA_001588195.1	Typhimurium	CFSAN033909	
GCA_001588215.1	Typhimurium	CFSAN033908	
GCA_001588235.1	Typhimurium	CFSAN033906	
GCA_001588255.1	Typhimurium	CFSAN033905	
GCA_001588275.1	Typhimurium	CFSAN033904	
GCA_001588285.1	Typhimurium	CFSAN033903	
GCA_001588315.1	Typhimurium	CFSAN033902	
GCA_001588335.1	Typhimurium	CFSAN033900	
GCA_001588355.1	Typhimurium	CFSAN033899	
GCA_001588365.1	Typhimurium	CFSAN033898	
GCA_001588395.1	Typhimurium	CFSAN033895	
GCA_001588415.1	Typhimurium	CFSAN033894	
GCA_001588435.1	Typhimurium	CFSAN033889	
GCA_001588455.1	Typhimurium	CFSAN033887	
GCA_001588475.1	Typhimurium	CFSAN033886	
GCA_001588515.1	Typhimurium	CFSAN033878	
GCA_001588535.1	Typhimurium	CFSAN033875	
GCA_001588565.1	Typhimurium	CFSAN033859	
GCA_001594425.1	Typhimurium	CFSAN033939	
GCA_001594455.1	Typhimurium	CFSAN033928	
GCA_001594505.1	Typhimurium	CFSAN033923	
GCA_001594515.1	Typhimurium	CFSAN033920	
GCA_001594585.1	Typhimurium	CFSAN033907	
GCA_001594595.1	Typhimurium	CFSAN033901	
GCA_001594615.1	Typhimurium	CFSAN033897	
GCA_001594625.1	Typhimurium	CFSAN033896	
GCA_001594665.1	Typhimurium	CFSAN033893	
GCA_001594685.1	Typhimurium	CFSAN033891	

GCA_001594695.1	Typhimurium	CFSAN033890	
GCA_001594715.1	Typhimurium	CFSAN033888	
GCA_001594745.1	Typhimurium	CFSAN033884	
GCA_001594765.1	Typhimurium	CFSAN033883	
GCA_001594775.1	Typhimurium	CFSAN033882	
GCA_001594795.1	Typhimurium	CFSAN033881	
GCA_001594825.1	Typhimurium	CFSAN033880	
GCA_001594845.1	Typhimurium	CFSAN033879	
GCA_001594855.1	Typhimurium	CFSAN033877	
GCA_001594875.1	Typhimurium	CFSAN033876	
GCA_001595015.1	Typhimurium	CFSAN033867	
GCA_001595075.1	Typhimurium	CFSAN033864	
GCA_001595115.1	Typhimurium	CFSAN033862	
GCA_001595145.1	Typhimurium	CFSAN033861	
GCA_001595185.1	Typhimurium	CFSAN033858	
GCA_001595225.1	Typhimurium	CFSAN033856	
GCA_001614025.1	Typhimurium	2012CEB2427SAL	
GCA_001614175.1	Typhimurium	2012CEB432SAL	
GCA_001617585.1	Typhimurium	RM9437	
GCA_001623645.1	Typhimurium	CDC_2010K_1587	
GCA_001623685.1	Typhimurium	CDC_2011K_1702	
GCA_001623705.1	Typhimurium	USDA_ARS_USMARC_1880	
GCA_001623725.1	Typhimurium	USDA_ARS_USMARC_1808	
GCA_001623745.2	Typhimurium	USDA_ARS_USMARC_1810	
GCA_001623765.2	Typhimurium	USDA_ARS_USMARC_1898	
GCA_001623775.1	Typhimurium	CDC_2009K_2059	
GCA_001623805.1	Typhimurium	CDC_2009K_1640	
GCA_001623825.2	Typhimurium	CDC_H2662	
GCA_001623845.1	Typhimurium	USDA_ARS_USMARC_1896	
GCA_001691985.1	Typhimurium	STy02	
GCA_001692065.1	Typhimurium	STy01	
GCA_001692145.1	Typhimurium	STy03	
GCA_001705365.1	Typhimurium	NC983	

GCA_001714645.1	Typhimurium	CFSAN033855	
GCA_001729025.1	Typhimurium	ST397	
GCA_001729035.1	Typhimurium	ST452	
GCA_001729045.1	Typhimurium	ST478	
GCA_001729225.1	Typhimurium	ST931R	
GCA_001755865.1	Typhimurium	CFSAN008084	
GCA_001755875.1	Typhimurium	CFSAN008071	
GCA_001755885.1	Typhimurium	CFSAN008089	
GCA_001755895.1	Typhimurium	CFSAN008074	
GCA_001755945.1	Typhimurium	CFSAN008079	
GCA_001755955.1	Typhimurium	CFSAN008080	
GCA_001755985.1	Typhimurium	CFSAN008073	
GCA_001755995.1	Typhimurium	CFSAN008083	
GCA_001756025.1	Typhimurium	CFSAN008081	
GCA_001756035.1	Typhimurium	CFSAN008086	
GCA_001756065.1	Typhimurium	CFSAN008076	
GCA_001756075.1	Typhimurium	CFSAN008077	
GCA_001756105.1	Typhimurium	CFSAN008085	
GCA_001756125.1	Typhimurium	CFSAN008075	
GCA_001756135.1	Typhimurium	CFSAN008088	
GCA_001756185.1	Typhimurium	CFSAN008072	
GCA_001756205.1	Typhimurium	CFSAN008087	
GCA_001756215.1	Typhimurium	CFSAN008078	
GCA_001758445.1	Typhimurium	TT9097	
GCA_001758525.1	Typhimurium	TT6675	
GCA_001884505.1	Typhimurium	SL7207	
GCA_001886995.1	Typhimurium	22495	
GCA_001887015.1	Typhimurium	22792	
GCA_001922185.1	Typhimurium	RM10961	
GCA_001954895.1	Typhimurium	R9_3245_R1	
GCA_001955195.1	Typhimurium	R8_0865_R1	
GCA_001955215.1	Typhimurium	R8_7307_R1	
GCA_001955225.1	Typhimurium	R8_8387_R1	

GCA_001955265.1	Typhimurium	R8_9118_R1	
GCA_001955295.1	Typhimurium	R8_9801_R1	
GCA_001955305.1	Typhimurium	R8_9815_R1	
GCA_001955375.1	Typhimurium	R9_3244_R1	
GCA_001955385.1	Typhimurium	R9_3246_R1	
GCA_001955395.1	Typhimurium	R9_3247_R1	
GCA_001955415.1	Typhimurium	R9_3248_R1	
GCA_001955455.1	Typhimurium	R9_3249_R1	
GCA_001955465.1	Typhimurium	R9_3251_R1	
GCA_001955475.1	Typhimurium	R9_3252_R1	
GCA_001981435.1	Typhimurium	SAL58370	
GCA_001989635.1	Typhimurium	81741	
GCA_001997115.1	Typhimurium	ATCC_14028	
GCA_002009155.1	Typhimurium	RM10607	
GCA_002018325.1	Typhimurium	CCRJ_26	
GCA_002018355.1	Typhimurium	PPRJ_27	
GCA_002025495.1	Typhimurium	LT2_KO_STM2896_STM0928	
GCA_002028525.1	Typhimurium	SSH006	
GCA_002028635.1	Typhimurium	LT2_KO_STM3664	
GCA_002029675.1	Typhimurium	LT2_KO_STM2896_STM3664	
GCA_002029715.1	Typhimurium	LT2_KO_STM1182_STM3664	
GCA_002030225.1	Typhimurium	var_5_BCW_2243	
GCA_002030375.1	Typhimurium	BCW_2215	
GCA_002030385.1	Typhimurium	N32044	
GCA_002030605.1	Typhimurium	var_5_BCW_2192	
GCA_002030885.1	Typhimurium	BCW_2218	
GCA_002030995.1	Typhimurium	var_5_BCW_2187	
GCA_002031265.1	Typhimurium	var_5_BCW_2163	
GCA_002031405.1	Typhimurium	var_5_N29331	
GCA_002031525.1	Typhimurium	var_5_BCW_2131	
GCA_002031565.1	Typhimurium	var_5_BCW_2120	
GCA_002031925.1	Typhimurium	var_5_BCW_2073	
GCA_002031955.1	Typhimurium	var_5_BCW_2064	

GCA_002032065.1	Typhimurium	var_5_BCW_2054	
GCA_002032165.1	Typhimurium	var_5_BCW_2050	
GCA_002032225.1	4,5,12:i	BCW_2045	
GCA_002032235.1	4,5,12:i	BCW_2044	
GCA_002032435.1	Typhimurium	var_5_BCW_2025	
GCA_002032705.1	Typhimurium	var_5_BCW_2172	
GCA_002032775.1	Typhimurium	var_5_BCW_2122	
GCA_002032875.1	Typhimurium	var_5_BCW_2095	
GCA_002032965.1	Typhimurium	var_5_BCW_2069	
GCA_002032975.1	Typhimurium	BCW_2085	
GCA_002032985.1	Typhimurium	var_5_BCW_2084	
GCA_002033065.1	Typhimurium	var_5_BCW_2065	
GCA_002033165.1	Typhimurium	var_5_BCW_2035	
GCA_002034905.1	4,5,12:i	BCW_1583	
GCA_002034915.1	4,5,12:i	BCW_1582	
GCA_002034965.1	4,5,12:i	BCW_1581	
GCA_002034975.1	4,5,12:i	BCW_1580	
GCA_002034985.1	Typhimurium	DT104	
GCA_002035025.1	Typhimurium	BCW_1574	
GCA_002035035.1	Typhimurium	BCW_1572	
GCA_002035055.1	Typhimurium	BCW_1571	
GCA_002035105.1	Typhimurium	BCW_1570	
GCA_002035115.1	Typhimurium	BCW_1569	
GCA_002035135.1	Typhimurium	BCW_1567	
GCA_002035415.1	Typhimurium	BCW_1573	
GCA_002035445.1	Typhimurium	BCW_1564	
GCA_002036205.1	Typhimurium	BCW_1511	
GCA_002036315.1	Typhimurium	BCW_2427	
GCA_002036765.1	Copenhagen	SAL3328	
GCA_002056945.1	Typhimurium	BCW_4365	
GCA_002057985.1	Typhimurium	BCW_4372	
GCA_002058005.1	Typhimurium	var_5_BCW_4379	
GCA_002058075.1	Typhimurium	var_5_BCW_4364	

GCA_002058205.1	Typhimurium	BCW_4352	
GCA_002058595.1	Typhimurium	var_5_BCW_4346	
GCA_002059555.1	Typhimurium	BCW_3990	
GCA_002060305.1	Copenhagen	BCW_3401	
GCA_002062795.1	Typhimurium	BCW_2836	
GCA_002062935.1	Typhimurium	BCW_2825	
GCA_002063225.1	4,5,12:i	BCW_2861	
GCA_002063425.1	Typhimurium	BCW_2824	
GCA_002063625.1	Typhimurium	BCW_2804	
GCA_002063655.1	Typhimurium	BCW_2800	
GCA_002063665.1	Typhimurium	BCW_2798	
GCA_002063685.1	Typhimurium	BCW_2799	
GCA_002063715.1	Typhimurium	BCW_2797	
GCA_002063745.1	Typhimurium	BCW_2795	
GCA_002065035.1	Typhimurium	BCW_2801	
GCA_002065055.1	Typhimurium	BCW_2796	
GCA_002066505.1	Typhimurium	BCW_2626	
GCA_002066795.1	4,5,12:i	BCW_2618	
GCA_002074595.1	Typhimurium	LT2_KO_STM4582	
GCA_002090935.1	Typhimurium	R8_0763_R1	
GCA_002090945.1	Typhimurium	R8_5213_R1	
GCA_002090955.1	Typhimurium	R8_0764_R1	
GCA_002090995.1	Typhimurium	R8_6089_R1	
GCA_002091025.1	Typhimurium	R8_8073_R1	
GCA_002091045.1	Typhimurium	R9_3272_R1	
GCA_002091075.1	Typhimurium	R9_3270_R1	
GCA_002091095.1	Typhimurium	R9_3274_R1	
GCA_002091115.1	Typhimurium	R9_3275_R1	
GCA_002091125.1	Typhimurium	R9_3276_R1	
GCA_002091185.1	Typhimurium	R9_3277_R1	
GCA_002091215.1	Typhimurium	R8_0784_R1	
GCA_002091235.1	Typhimurium	R9_0437_R1	
GCA_002091255.1	Typhimurium	R8_8081_R1	

GCA_002091275.1	Typhimurium	R9_0042_R1	
GCA_002091285.1	Typhimurium	R9_3269_R1	
GCA_002091315.1	Typhimurium	R9_3278_R1	
GCA_002091335.1	Typhimurium	R9_3271_R1	
GCA_002091345.1	Typhimurium	R9_3273_R1	
GCA_002105815.1	Copenhagen	NY_FSL_C7_1193	
GCA_002156155.1	Typhimurium	NCIMB_1024	
GCA_002193525.1	Typhimurium	LT18	
GCA_002193535.1	Typhimurium	LT20	
GCA_002193545.1	Typhimurium	LT1	
GCA_002193595.1	Typhimurium	LT22	
GCA_002193605.1	Typhimurium	LT21	
GCA_002193615.1	Typhimurium	LT19	
GCA_002209225.1	Typhimurium	FDAARGOS_321	
GCA_002210025.1	Typhimurium	TW_Stm6	
GCA_002252345.2	Typhimurium	PP_BR027	
GCA_002252355.2	Typhimurium	PP_BR007	
GCA_002252365.2	Typhimurium	PP_BR026	
GCA_002252405.2	Typhimurium	PP_BR032	
GCA_002252465.2	Typhimurium	PP_BR045	
GCA_002252605.2	Typhimurium	PP_BR031	
GCA_002260565.1	Typhimurium	STy013	
GCA_002260985.1	Typhimurium	STy06	
GCA_002260995.1	Typhimurium	STy015	
GCA_002261025.1	Typhimurium	STy014	
GCA_002264585.1	Typhimurium	D15_052810	
GCA_002265755.1	Typhimurium	SE697	
GCA_002265805.1	Typhimurium	SE36N	
GCA_002270095.1	Typhimurium	STy13	
GCA_002283545.1	Typhimurium	GMR_S_1454	
GCA_002283795.1	Typhimurium	GMR_S_1257	
GCA_002289225.1	Typhimurium	LT2	
GCA_002300245.2	Typhimurium	PP_BR062	

GCA_002300255.2	Typhimurium	PP_BR057	
GCA_002300265.2	Typhimurium	PP_BR060	
GCA_002300275.2	Typhimurium	PP_BR063	
GCA_002300295.2	Typhimurium	PP_BR076	
GCA_002313125.1	Typhimurium	WW012	
GCA_002631105.1	Typhimurium	SE376	
GCA_002631125.1	Typhimurium	SE426	
GCA_002631135.1	Typhimurium	SE389	
GCA_002634225.1	Typhimurium	SE373	
GCA_002634305.1	Typhimurium	SE368	
GCA_002723705.1	Typhimurium	SE492	
GCA_002740635.1	Typhimurium	CFSAN070646	
GCA_002798515.1	Typhimurium	CFSAN068041	
GCA_002798525.1	Typhimurium	CFSAN068042	
GCA_002798565.1	Typhimurium	CFSAN068043	
GCA_002798595.1	Typhimurium	CFSAN068036	
GCA_002798615.1	Typhimurium	CFSAN068037	
GCA_002798625.1	Typhimurium	CFSAN068032	
GCA_002798635.1	Typhimurium	CFSAN068029	
GCA_002798695.1	Typhimurium	CFSAN068047	
GCA_002798715.1	Typhimurium	CFSAN068045	
GCA_002798725.1	Typhimurium	CFSAN068044	
GCA_002798755.1	Typhimurium	CFSAN068046	
GCA_002798775.1	Typhimurium	CFSAN068040	
GCA_002798835.1	Typhimurium	CFSAN068033	
GCA_002798855.1	Typhimurium	CFSAN068030	
GCA_002798875.1	Typhimurium	CFSAN068034	
GCA_002809915.1	Typhimurium	CFSAN068031	
GCA_002861645.1	Typhimurium	388	
GCA_900002655.1	Typhimurium	STM16	
GCA_900002695.1	Typhimurium	STM45	
GCA_900002715.1	Typhimurium	STM44	
GCA_900002735.1	Typhimurium	STM19	

GCA_900002765.1	Typhimurium	STM143	
GCA_900002785.1	Typhimurium	STM134	
GCA_900002905.1	Typhimurium	STM194	
GCA_900002955.1	Typhimurium	STM203	
GCA_900003015.1	Typhimurium	STM201	
GCA_900003025.1	Typhimurium	STM126	
GCA_900003055.1	Typhimurium	STM184	
GCA_900003075.1	Typhimurium	STM75	
GCA_900003095.1	Typhimurium	STM177	
GCA_900003125.1	Typhimurium	STM7	
GCA_900003135.1	Typhimurium	STM12	
GCA_900003195.1	Typhimurium	STM22	
GCA_900003295.1	Typhimurium	STM8	
GCA_900003305.1	Typhimurium	STM9	
GCA_900003325.1	Typhimurium	STM211	
GCA_900003755.1	Typhimurium	STM21	
GCA_900003975.1	Typhimurium	STM2	
GCA_900005365.1	Typhimurium	STM4	
GCA_900006185.1	Typhimurium	STM216	
GCA_900007195.1	Typhimurium	STM217	
GCA_900007275.1	Typhimurium	STM218	
GCA_900007425.1	Typhimurium	STM169	
GCA_900007445.1	Typhimurium	STM57	
GCA_900015205.1	Typhimurium	STM18	
GCA_900019235.1	Typhimurium	STM20	
GCA_900166885.1	Typhimurium	VNB151_sc_2315230	
GCA_900184385.1	Typhimurium	STMU2UK	
*These strains were inlcuded in Figure 3B, but not in Figure 3A because they were considered duplicates based on the serovar-level alignment.			

Table S3. BEAST model comparison.					
		log Marginal Likelihood Estimate		log Bayes Factor	
**Clock model**	**Tree model**	**Path sampling**	**Stepping-stone**	**Path sampling**	**Stepping-stone**
Strict clock	Constant	-6,397,396.39	-6,397,394.83	0.00	0.00
Strict clock	Skyline (10)	-6,397,394.80	-6,397,392.51	1.59	2.32
Strict clock	SkyGrid (20)	-6,397,397.11	-6,397,396.97	-0.72	-2.14
Relaxed, UCLN	Constant	-6,397,217.10	-6,397,214.57	179.29	180.26
Relaxed, UCLN	Skyline (10)	-6,397,217.86	-6,397,216.50	178.53	178.32
**Relaxed, UCLN**	**SkyGrid (20)**	**-6,397,212.32**	**-6,397,210.87**	**184.06**	**183.96**
Relaxed, UCLN = uncorrelated relaxed clock with lognormally distributed rate categories					
Constant = constant size coalescent model					
Skyline (10) = Bayesian Skyline coalescent model with 10 groups					
SkyGrid (20) = Bayesian SkyGrid coalescent model with 20 parameters					

Table S4. Genes putatively unique to the S. Typhimurium variant isolated from olive ridleys. Contig names and locus tags refer to the de novo assebled contigs and PGAP annotations for LOL16001 (GenBank:QLZW00000000).							
				**Closest Blastn hit**			
**Contig Name (LOL16001)**	**Locus Tag (LOL16001)**	**Product (PGAP)**	**% nt unique**	**Accession**	**Name**	**% Aligned**	**% Identity**
NODE_13_length_147960_cov_17.518	DP141_12505	hypothetical protein	0.4949	KJ457259.1	Macaca mulatta isolate Rh28626 clone 3596 major histocompatibility complex-B genomic sequence	69.39	94.85
NODE_1_length_435680_cov_19.0388	DP141_00005	PapC protein	0.8214	CP029981.1	Escherichia coli strain 99-3165 chromosome	100	97.62
NODE_1_length_435680_cov_19.0388	DP141_01595	hypothetical protein	1	CP024165.1	Salmonella enterica subsp. enterica serovar Gaminara strain CFSAN070644 chromosome	100	99.75
NODE_1_length_435680_cov_19.0388	DP141_01600	integrase	1	CP024165.1	Salmonella enterica subsp. enterica serovar Gaminara strain CFSAN070644 chromosome	100	99.57
NODE_1_length_435680_cov_19.0388	DP141_01605	XRE family transcriptional regulator	1	CP024165.1	Salmonella enterica subsp. enterica serovar Gaminara strain CFSAN070644 chromosome	100	100
NODE_1_length_435680_cov_19.0388	DP141_01610	DNA-binding protein	1	CP024165.1	Salmonella enterica subsp. enterica serovar Gaminara strain CFSAN070644 chromosome	100	100
NODE_1_length_435680_cov_19.0388	DP141_01615	hypothetical protein	1	CP024165.1	Salmonella enterica subsp. enterica serovar Gaminara strain CFSAN070644 chromosome	100	99.69
NODE_1_length_435680_cov_19.0388	DP141_01620	hypothetical protein	1	CP024165.1	Salmonella enterica subsp. enterica serovar Gaminara strain CFSAN070644 chromosome	100	99.59
NODE_1_length_435680_cov_19.0388	DP141_01625	LapA family protein	1	CP006608.1	Salmonella bongori N268-08	100	100

NODE_1_length_435680_cov_19.0388	DP141_01630	hypothetical protein	1	CP022117.1	Salmonella enterica subsp. enterica serovar Macclesfield str. S-1643	100	98.41
NODE_1_length_435680_cov_19.0388	DP141_01635	hypothetical protein	0.9341	CP024165.1	Salmonella enterica subsp. enterica serovar Gaminara strain CFSAN070644 chromosome	100	91.9
NODE_1_length_435680_cov_19.0388	DP141_01640	hypothetical protein	0.8961	CP023345.1	Salmonella enterica subsp. diarizonae strain HZS154 chromosome	100	98.21
NODE_1_length_435680_cov_19.0388	DP141_01645	DNA adenine methylase	0.9167	CP022117.1	Salmonella enterica subsp. enterica serovar Macclesfield str. S-1643	95.76	87.94
NODE_1_length_435680_cov_19.0388	DP141_01650	AsnC family protein	1	CP024165.1	Salmonella enterica subsp. enterica serovar Gaminara strain CFSAN070644 chromosome	100	95.44
NODE_1_length_435680_cov_19.0388	DP141_01655	DNA cytosine methyltransferase	1	CP024165.1	Salmonella enterica subsp. enterica serovar Gaminara strain CFSAN070644 chromosome	100	95.56
NODE_1_length_435680_cov_19.0388	DP141_01660	replication endonuclease	0.9725	CP024165.1	Salmonella enterica subsp. enterica serovar Gaminara strain CFSAN070644 chromosome	99.96	97.81
NODE_1_length_435680_cov_19.0388	DP141_01665	hypothetical protein	1	CP022117.1	Salmonella enterica subsp. enterica serovar Macclesfield str. S-1643	94.13	89.53
NODE_1_length_435680_cov_19.0388	DP141_01670	hypothetical protein	1	CP012344.1	Salmonella enterica subsp. enterica serovar Choleraesuis str. ATCC 10708	98.46	87.5
NODE_1_length_435680_cov_19.0388	DP141_01675	hypothetical protein	1	CP012344.1	Salmonella enterica subsp. enterica serovar Choleraesuis str. ATCC 10708	100	91.78
NODE_1_length_435680_cov_19.0388	DP141_01680	hypothetical protein	1	CP024165.1	Salmonella enterica subsp. enterica serovar Gaminara strain CFSAN070644 chromosome	100	96.57

NODE_1_length_435680_cov_19.0388	DP141_01685	phage portal protein	0.9105	CP018655.1	Salmonella enterica subsp. enterica serovar Enteritidis strain 81-1706 chromosome	100	98.67
NODE_1_length_435680_cov_19.0388	DP141_01690	oxidoreductase	1	CP022117.1	Salmonella enterica subsp. enterica serovar Macclesfield str. S-1643	100	98.96
NODE_1_length_435680_cov_19.0388	DP141_01695	phage capsid protein	1	CP006608.1	Salmonella bongori N268-08	100	99.76
NODE_1_length_435680_cov_19.0388	DP141_01700	phage major capsid protein, P2 family	1	CP019192.1	Salmonella enterica subsp. enterica serovar Rubislaw str. ATCC 10717	100	98.8
NODE_1_length_435680_cov_19.0388	DP141_01705	terminase	1	CP018655.1	Salmonella enterica subsp. enterica serovar Enteritidis strain 81-1706 chromosome	82.16	98.5
NODE_1_length_435680_cov_19.0388	DP141_01710	capsid assembly protein	1	CP024165.1	Salmonella enterica subsp. enterica serovar Gaminara strain CFSAN070644 chromosome	100	98.99
NODE_1_length_435680_cov_19.0388	DP141_01715	phage tail protein	1	CP009561.1	Salmonella enterica subsp. enterica serovar Newport str. CVM N18486	100	99
NODE_1_length_435680_cov_19.0388	DP141_01720	phage holin, lambda family	1	CP024165.1	Salmonella enterica subsp. enterica serovar Gaminara strain CFSAN070644 chromosome	100	95.5
NODE_1_length_435680_cov_19.0388	DP141_01725	lysozyme	1	CP019417.1	Salmonella enterica subsp. enterica serovar Wandsworth str. SA20092095	100	98.87
NODE_1_length_435680_cov_19.0388	DP141_01730	lysis protein	1	CP009561.1	Salmonella enterica subsp. enterica serovar Newport str. CVM N18486	100	99.19
NODE_1_length_435680_cov_19.0388	DP141_01735	phage tail protein	1	CP022117.1	Salmonella enterica subsp. enterica serovar Macclesfield str. S-1643	100	98.53

NODE_1_length_435680_cov_19.0388	DP141_01740	phage virion morphogenesis protein	1	CP009561.1	Salmonella enterica subsp. enterica serovar Newport str. CVM N18486	100	98.9
NODE_1_length_435680_cov_19.0388	DP141_01745	phage baseplate assembly protein V	1	CP019192.1	Salmonella enterica subsp. enterica serovar Rubislaw str. ATCC 10717	100	99.15
NODE_1_length_435680_cov_19.0388	DP141_01750	baseplate assembly protein	1	CP024165.1	Salmonella enterica subsp. enterica serovar Gaminara strain CFSAN070644 chromosome	100	99.46
NODE_1_length_435680_cov_19.0388	DP141_01755	baseplate assembly protein	1	CP024165.1	Salmonella enterica subsp. enterica serovar Gaminara strain CFSAN070644 chromosome	100	99.55
NODE_1_length_435680_cov_19.0388	DP141_01760	phage tail protein I	1	CP018655.1	Salmonella enterica subsp. enterica serovar Enteritidis strain 81-1706 chromosome	100	98.86
NODE_1_length_435680_cov_19.0388	DP141_01765	hypothetical protein	0.9585	CP019417.1	Salmonella enterica subsp. enterica serovar Wandsworth str. SA20092095	55.88	96.44
NODE_1_length_435680_cov_19.0388	DP141_01780	UDP-glucose--(glucosyl)LPSalpha-1,2-glucosyltransferase	1	CP019413.1	Salmonella enterica subsp. enterica serovar Krefeld str. SA20030536	100	94.58
NODE_1_length_435680_cov_19.0388	DP141_01785	oxidoreductase	1	CP019192.1	Salmonella enterica subsp. enterica serovar Rubislaw str. ATCC 10717	100	96.52
NODE_1_length_435680_cov_19.0388	DP141_01790	phage tail tape measure protein	1	CP024165.1	Salmonella enterica subsp. enterica serovar Gaminara strain CFSAN070644 chromosome	99.93	97.94
NODE_1_length_435680_cov_19.0388	DP141_01795	GpE family phage tail protein	1	CP024165.1	Salmonella enterica subsp. enterica serovar Gaminara strain CFSAN070644 chromosome	100	100
NODE_1_length_435680_cov_19.0388	DP141_01800	phage tail protein	1	CP022117.1	Salmonella enterica subsp. enterica serovar Macclesfield str. S-1643	100	99.44

NODE_1_length_435680_cov_19.0388	DP141_01805	phage major tail tube protein	1	CP006608.1	Salmonella bongori N268-08	100	99.22
NODE_1_length_435680_cov_19.0388	DP141_01810	phage tail protein	1	CP006608.1	Salmonella bongori N268-08	100	97.22
NODE_1_length_435680_cov_19.0388	DP141_01815	phage late control D family protein	1	CP024165.1	Salmonella enterica subsp. enterica serovar Gaminara strain CFSAN070644 chromosome	100	99.13
NODE_1_length_435680_cov_19.0388	DP141_01820	hypothetical protein	1	CP024165.1	Salmonella enterica subsp. enterica serovar Gaminara strain CFSAN070644 chromosome	100	99.29
NODE_1_length_435680_cov_19.0388	DP141_01825	Hok/Gef family protein	1	CP019417.1	Salmonella enterica subsp. enterica serovar Wandsworth str. SA20092095	100	99.29
NODE_20_length_99538_cov_23.3276	DP141_17110	monooxygenase	1	CP022660.1	Salmonella enterica subsp. enterica strain RM11060 plasmid pRM11060-2	97.9	99.32
NODE_20_length_99538_cov_23.3276	DP141_17115	N-acetyltransferase	1	CP022660.1	Salmonella enterica subsp. enterica strain RM11060 plasmid pRM11060-2	100	99.78
NODE_20_length_99538_cov_23.3276	DP141_17120	hypothetical protein	1	CP024167.1	Salmonella enterica subsp. enterica serovar Gaminara strain CFSAN070644 plasmid pCFSAN024441_02	100	99.45
NODE_20_length_99538_cov_23.3276	DP141_17125	hypothetical protein	1	CP024167.1	Salmonella enterica subsp. enterica serovar Gaminara strain CFSAN070644 plasmid pCFSAN024441_02	100	96.48
NODE_20_length_99538_cov_23.3276	DP141_17130	hypothetical protein	1	CP024167.1	Salmonella enterica subsp. enterica serovar Gaminara strain CFSAN070644 plasmid pCFSAN024441_02	100	98.32
NODE_20_length_99538_cov_23.3276	DP141_17135	replication initiator protein RepA	1	CP026394.1	Klebsiella pneumoniae strain KPNIH48 plasmid pKPC-e937	100	96.6

NODE_20_length_99538_cov_23.3276	DP141_17140	RepA leader peptide Tap	1	CP017992.1	Enterobacter cloacae complex sp. ECNIH7 plasmid pENT-2c5	100	98.72
NODE_20_length_99538_cov_23.3276	DP141_17145	transcriptional regulator	0.9072	CP026394.1	Klebsiella pneumoniae strain KPNIH48 plasmid pKPC-e937	100	99.16
NODE_20_length_99538_cov_23.3276	DP141_17150	phospholipase D family protein	0.9007	CP019175.1	Salmonella enterica subsp. enterica serovar Give strain CFSAN024229 plasmid pCFSAN024229	99.82	97.34
NODE_20_length_99538_cov_23.3276	DP141_17155	DsbA family protein	1	KY680213.1	Klebsiella aerogenes strain N15-1247 plasmid pN151247-1	91.02	79.18
NODE_20_length_99538_cov_23.3276	DP141_17160	conjugal transfer protein	1	KX808482.1	Escherichia coli O55:H7 strain 122262 plasmid	92.11	68.67
NODE_20_length_99538_cov_23.3276	DP141_17165	type-F conjugative transfer system pilinacetylase TraX	1	CP018634.1	Salmonella enterica subsp. enterica serovar Enteritidis strain 49-2444 plasmid pSE49-2444	61.53	75.38
NODE_20_length_99538_cov_23.3276	DP141_17170	conjugative transfer relaxase/helicase TraI	1	CP022660.1	Salmonella enterica subsp. enterica strain RM11060 plasmid pRM11060-2	99.23	76.43
NODE_20_length_99538_cov_23.3276	DP141_17175	type IV conjugative transfer system couplingprotein TraD	0.7701	CP022660.1	Salmonella enterica subsp. enterica strain RM11060 plasmid pRM11060-2	99.95	86.72
NODE_20_length_99538_cov_23.3276	DP141_17180	complement resistance protein TraT	1	CP022660.1	Salmonella enterica subsp. enterica strain RM11060 plasmid pRM11060-2	100	91.53
NODE_20_length_99538_cov_23.3276	DP141_17185	hypothetical protein	1	CP018634.1	Salmonella enterica subsp. enterica serovar Enteritidis strain 49-2444 plasmid pSE49-2444	100	83.65
NODE_20_length_99538_cov_23.3276	DP141_17190	conjugal transfer protein TraG	1	CP018634.1	Salmonella enterica subsp. enterica serovar Enteritidis strain 49-2444 plasmid pSE49-2444	99.93	84.69

NODE_20_length_99538_cov_23.3276	DP141_17195	conjugal transfer protein TraH	0.5655	HF969016.1	Salmonella enterica subsp. enterica serovar Bovismorbificans str. 3114 plasmid pVIRBov complete sequence	94.76	89.63
NODE_20_length_99538_cov_23.3276	DP141_17200	hypothetical protein	1				
NODE_20_length_99538_cov_23.3276	DP141_17205	type-F conjugative transfer system pilinassembly thiol-disulfide isomerase TrbB	1	CP018634.1	Salmonella enterica subsp. enterica serovar Enteritidis strain 49-2444 plasmid pSE49-2444	83.77	78.96
NODE_20_length_99538_cov_23.3276	DP141_17210	type-F conjugative transfer system pilinchaperone TraQ	1	CP024157.1	Escherichia coli strain 14EC047 plasmid p14EC047b	93.2	89.42
NODE_20_length_99538_cov_23.3276	DP141_17215	type-F conjugative transfer system pilinassembly protein TraF	0.7333	CP021464.1	Salmonella enterica subsp. enterica serovar Typhimurium strain UGA14 plasmid pUGA14_2	99.73	86.76
NODE_20_length_99538_cov_23.3276	DP141_17220	conjugal transfer protein TrbE	1	CP018634.1	Salmonella enterica subsp. enterica serovar Enteritidis strain 49-2444 plasmid pSE49-2444	100	82.87
NODE_20_length_99538_cov_23.3276	DP141_17225	hypothetical protein	1	CP018634.1	Salmonella enterica subsp. enterica serovar Enteritidis strain 49-2444 plasmid pSE49-2444	99.58	69.81
NODE_20_length_99538_cov_23.3276	DP141_17230	type-F conjugative transfer system mating-pairstabilization protein TraN	0.9187	CP022660.1	Salmonella enterica subsp. enterica strain RM11060 plasmid pRM11060-2	99.52	81.36
NODE_20_length_99538_cov_23.3276	DP141_17235	type-F conjugative transfer system pilinassembly protein TrbC	0.8302	CP022660.1	Salmonella enterica subsp. enterica strain RM11060 plasmid pRM11060-2	100	93.17
NODE_20_length_99538_cov_23.3276	DP141_17240	conjugal transfer protein TraU	0.6697	CP022660.1	Salmonella enterica subsp. enterica strain RM11060 plasmid pRM11060-2	100	89.12
NODE_20_length_99538_cov_23.3276	DP141_17245	type-F conjugative transfer system protein TraW	0.7222	CP022660.1	Salmonella enterica subsp. enterica strain RM11060 plasmid pRM11060-2	95.71	91.04

NODE_20_length_99538_cov_23.3276	DP141_17250	type-F conjugative transfer system protein TrbI	1	CP010222.1	Escherichia coli strain M19 plasmid A	100	85.27
NODE_20_length_99538_cov_23.3276	DP141_17255	type IV secretion system protein TraC	0.847	LM996828.1	Escherichia coli genome assembly FHI71	99.89	88.27
NODE_20_length_99538_cov_23.3276	DP141_17260	hypothetical protein	1	CP022660.1	Salmonella enterica subsp. enterica strain RM11060 plasmid pRM11060-2	85.01	71.68
NODE_20_length_99538_cov_23.3276	DP141_17265	hypothetical protein	1				
NODE_20_length_99538_cov_23.3276	DP141_17270	type IV conjugative transfer system proteinTraV	1	CP022660.1	Salmonella enterica subsp. enterica strain RM11060 plasmid pRM11060-2	95.26	79.08
NODE_20_length_99538_cov_23.3276	DP141_17275	conjugal transfer protein TraP	1	CP022137.1	Salmonella enterica subsp. diarizonae serovar 65:c:z str. SA20044251 plasmid unnamed2	44.22	75.92
NODE_20_length_99538_cov_23.3276	DP141_17280	conjugal transfer protein TraB	1	LK931337.1	Citrobacter koseri genome assembly PRJEB6512_assembly_1	98.47	84.22
NODE_20_length_99538_cov_23.3276	DP141_17285	type-F conjugative transfer system secretinTraK	1	CP019456.1	Escherichia coli strain FHI_NMBU_03 plasmid pFHI_NMBU_03_1	98.67	83.93
NODE_20_length_99538_cov_23.3276	DP141_17290	type IV conjugative transfer system proteinTraE	1	CP012348.1	Salmonella enterica subsp. enterica serovar Pullorum str. ATCC 9120 plasmid pCFSAN000725_01	100	85.71
NODE_20_length_99538_cov_23.3276	DP141_17295	type IV conjugative transfer system proteinTraL	0.4167	LK931337.1	Citrobacter koseri genome assembly PRJEB6512_assembly_1	99.36	90.65
NODE_20_length_99538_cov_23.3276	DP141_17300	type IV conjugative transfer system pilin TraA	1	CP028152.1	Salmonella enterica subsp. enterica serovar Enteritidis str. RM2968 plasmid pRM2968-1	100	82.37

NODE_20_length_99538_cov_23.3276	DP141_17305	conjugal transfer protein TraY	1	CP029974.1	Escherichia coli strain 51008369SK1 plasmid p51008369SK1_A	44.76	75.53
NODE_20_length_99538_cov_23.3276	DP141_17310	hypothetical protein	1				
NODE_20_length_99538_cov_23.3276	DP141_17315	conjugal transfer protein TraM	1	CP012348.1	Salmonella enterica subsp. enterica serovar Pullorum str. ATCC 9120 plasmid pCFSAN000725_01	98.97	76.68
NODE_20_length_99538_cov_23.3276	DP141_17320	lytic transglycosylase	0.9294	CP010222.1	Escherichia coli strain M19 plasmid A	88.43	81.37
NODE_20_length_99538_cov_23.3276	DP141_17325	SAM-dependent DNA methyltransferase	1	AP018582.1	Klebsiella pneumoniae MH13-055M plasmid pMH13-055M_1 DNA	97.71	90.27
NODE_20_length_99538_cov_23.3276	DP141_17330	hypothetical protein	1	CP023525.1	Cedecea neteri strain FDAARGOS_392 chromosome	100	98.5
NODE_20_length_99538_cov_23.3276	DP141_17335	hypothetical protein	1	CP017185.1	Enterobacter cloacae complex 'Hoffmann cluster IV' strain DSM 16690 plasmid pDSMZ16690	100	93.33
NODE_20_length_99538_cov_23.3276	DP141_17340	ammonia monooxygenase	1	JQ418541.1	Salmonella enterica subsp. salamae strain SGSC3045 plasmid pSGSC3045-121	100	89.63
NODE_20_length_99538_cov_23.3276	DP141_17345	type I toxin-antitoxin system hok family toxin	1	CP019175.1	Salmonella enterica subsp. enterica serovar Give strain CFSAN024229 plasmid pCFSAN024229	100	100
NODE_20_length_99538_cov_23.3276	DP141_17350	theronine dehydrogenase	1	CP017992.1	Enterobacter cloacae complex sp. ECNIH7 plasmid pENT-2c5	96.33	91.43
NODE_20_length_99538_cov_23.3276	DP141_17355	plasmid SOS inhibition protein A	1	CP006054.1	Salmonella enterica subsp. enterica serovar Bareilly str. CFSAN000189 plasmid unnamed	100	90.81

NODE_20_length_99538_cov_23.3276	DP141_17360	conjugation system SOS inhibitor PsiB	0.9241	CP019175.1	Salmonella enterica subsp. enterica serovar Give strain CFSAN024229 plasmid pCFSAN024229	97.48	98.82
NODE_20_length_99538_cov_23.3276	DP141_17365	chromosome partitioning protein ParB	0.7752	CP024166.1	Salmonella enterica subsp. enterica serovar Gaminara strain CFSAN070644 plasmid pCFSAN024441_01	99.18	94.42
NODE_20_length_99538_cov_23.3276	DP141_17370	DUF905 domain-containing protein	1	CP011654.1	Citrobacter freundii strain CAV1741 plasmid pCAV1741-101	97.65	89.96
NODE_20_length_99538_cov_23.3276	DP141_17375	single-stranded DNA-binding protein	1	CP011654.1	Citrobacter freundii strain CAV1741 plasmid pCAV1741-101	100	99.46
NODE_20_length_99538_cov_23.3276	DP141_17380	single-stranded DNA-binding protein	1	LT991960.1	Enterobacter cloacae complex bacterium isolate C45 genome assembly	100	98.89
NODE_20_length_99538_cov_23.3276	DP141_17390	hypothetical protein	1	CP019175.1	Salmonella enterica subsp. enterica serovar Give strain CFSAN024229 plasmid pCFSAN024229	100	96.3
NODE_20_length_99538_cov_23.3276	DP141_17395	DNA polymerase III subunit theta	1	CP006609.1	Salmonella bongori N268-08 plasmid RM1	100	97.32
NODE_20_length_99538_cov_23.3276	DP141_17400	hypothetical protein	1	CP026698.1	Citrobacter koseri strain AR_0025 plasmid unitig_2_pilon	100	99.48
NODE_20_length_99538_cov_23.3276	DP141_17405	antirestriction protein ArdA	1	CP026698.1	Citrobacter koseri strain AR_0025 plasmid unitig_2_pilon	100	99.8
NODE_20_length_99538_cov_23.3276	DP141_17410	hypothetical protein	1	CP004083.1	Enterobacteriaceae bacterium bta3-1	100	91.16
NODE_20_length_99538_cov_23.3276	DP141_17415	hypothetical protein	1	CP028555.1	Klebsiella variicola strain WCHKP19 chromosome	99.16	95.74

NODE_20_length_99538_cov_23.3276	DP141_17420	hypothetical protein	1	CP012266.1	Cronobacter dublinensis subsp. dublinensis LMG 23823	98.08	94.53
NODE_20_length_99538_cov_23.3276	DP141_17425	hypothetical protein	1	CP006054.1	Salmonella enterica subsp. enterica serovar Bareilly str. CFSAN000189 plasmid unnamed	100	99.23
NODE_20_length_99538_cov_23.3276	DP141_17430	DUF1380 domain-containing protein	1	CP017992.1	Enterobacter cloacae complex sp. ECNIH7 plasmid pENT-2c5	100	99.29
NODE_20_length_99538_cov_23.3276	DP141_17435	hypothetical protein	1	CP022035.1	Salmonella enterica subsp. enterica serovar Onderstepoort str. SA20060086 plasmid punamed2	100	99.55
NODE_20_length_99538_cov_23.3276	DP141_17440	DNA methylase	0.5015	CP006054.1	Salmonella enterica subsp. enterica serovar Bareilly str. CFSAN000189 plasmid unnamed	100	99.41
NODE_20_length_99538_cov_23.3276	DP141_17445	DNA breaking-rejoining protein	1	CP022035.1	Salmonella enterica subsp. enterica serovar Onderstepoort str. SA20060086 plasmid punamed2	100	97.77
NODE_20_length_99538_cov_23.3276	DP141_17450	peptidase	0.5154	CP026698.1	Citrobacter koseri strain AR_0025 plasmid unitig_2_pilon	100	99.53
NODE_20_length_99538_cov_23.3276	DP141_17460	hypothetical protein	1	CP024274.1	Escherichia coli strain F9792 plasmid unnamed	100	99.54
NODE_20_length_99538_cov_23.3276	DP141_17465	hypothetical protein	1	CP022035.1	Salmonella enterica subsp. enterica serovar Onderstepoort str. SA20060086 plasmid punamed2	100	99.32
NODE_20_length_99538_cov_23.3276	DP141_17470	chromosome partitioning protein ParB	1	CP017992.1	Enterobacter cloacae complex sp. ECNIH7 plasmid pENT-2c5	100	97.97
NODE_20_length_99538_cov_23.3276	DP141_17475	ParA family protein	1	CP024274.1	Escherichia coli strain F9792 plasmid unnamed	100	97.35

NODE_20_length_99538_cov_23.3276	DP141_17480	serine recombinase	1	CP00402 8.1	Salmonella enterica subsp. enterica serovar Javiana str. CFSAN001992 plasmid pCFSAN001992_2	92.09	96.2
NODE_20_length_99538_cov_23.3276	DP141_17485	hypothetical protein	1	CP02266 1.1	Salmonella enterica subsp. enterica strain RM11065 plasmid pRM11065-1	100	96.86
NODE_20_length_99538_cov_23.3276	DP141_17490	ISNCY family transposase	1	CP02266 1.1	Salmonella enterica subsp. enterica strain RM11065 plasmid pRM11065-1	99.46	90.51
NODE_20_length_99538_cov_23.3276	DP141_17495	hypothetical protein	0.6026	CP02639 4.1	Klebsiella pneumoniae strain KPNIH48 plasmid pKPC-e937	99.18	93.05
NODE_20_length_99538_cov_23.3276	DP141_17520	hypothetical protein	1	CP02911 2.1	Escherichia coli strain AR436 plasmid unnamed3	93.99	93.61
NODE_20_length_99538_cov_23.3276	DP141_17525	lipase family protein	1	CP01919 3.1	Salmonella enterica subsp. enterica serovar Rubislaw str. ATCC 10717 plasmid pATCC10717	100	99.24
NODE_20_length_99538_cov_23.3276	DP141_17530	hypothetical protein	1	CP01919 3.1	Salmonella enterica subsp. enterica serovar Rubislaw str. ATCC 10717 plasmid pATCC10717	100	98.57
NODE_20_length_99538_cov_23.3276	DP141_17535	hypothetical protein	1	CP01919 3.1	Salmonella enterica subsp. enterica serovar Rubislaw str. ATCC 10717 plasmid pATCC10717	100	99.55
NODE_20_length_99538_cov_23.3276	DP141_17540	lysis protein	1	CP01919 3.1	Salmonella enterica subsp. enterica serovar Rubislaw str. ATCC 10717 plasmid pATCC10717	100	98.67
NODE_20_length_99538_cov_23.3276	DP141_17545	hypothetical protein	1	CP02812 3.1	Escherichia coli O43 str. RM10042 plasmid pRM10042-3	100	76.19
NODE_20_length_99538_cov_23.3276	DP141_17550	bacteriocin immunity protein	1	CP02812 3.1	Escherichia coli O43 str. RM10042 plasmid pRM10042-3	100	94.44

NODE_20_length_99538_cov_23.3276	DP141_17555	bacteriocin immunity protein	1	CP021198.1	Escherichia coli strain H17 plasmid pH17-5	100	77.13
NODE_20_length_99538_cov_23.3276	DP141_17560	bacteriocin immunity protein	1	CP019193.1	Salmonella enterica subsp. enterica serovar Rubislaw str. ATCC 10717 plasmid pATCC10717	100	100
NODE_20_length_99538_cov_23.3276	DP141_17565	bacteriocin immunity protein	1	CP021198.1	Escherichia coli strain H17 plasmid pH17-5	96.51	78.31
NODE_20_length_99538_cov_23.3276	DP141_17570	hypothetical protein	1	CP019193.1	Salmonella enterica subsp. enterica serovar Rubislaw str. ATCC 10717 plasmid pATCC10717	71.2	93.68
NODE_20_length_99538_cov_23.3276	DP141_17575	hypothetical protein	1	CP000654.1	Enterobacter sp. 638 plasmid pENTE01	100	96.47
NODE_20_length_99538_cov_23.3276	DP141_17580	hypothetical protein	1	CP030347.1	Enterobacter cloacae strain AR_038 chromosome	69.43	74.5
NODE_20_length_99538_cov_23.3276	DP141_17585	IS66 family transposase	1	CP017279.1	Enterobacter ludwigii strain EN-119	98.81	90.06
NODE_20_length_99538_cov_23.3276	DP141_17590	IS3 family transposase	1	LS483475.1	Salmonella enterica subsp. salamae serovar Greenside strain NCTC9936 genome assembly	100	98.26
NODE_20_length_99538_cov_23.3276	DP141_17595	RepB family plasmid replication initiatorprotein	1	CP017721.1	Salmonella enterica subsp. enterica serovar Minnesota strain CFSAN017963 plasmid pCFSAN017963_01	100	99.65
NODE_20_length_99538_cov_23.3276	DP141_17600	phage integrase family protein	0.8802	CP017721.1	Salmonella enterica subsp. enterica serovar Minnesota strain CFSAN017963 plasmid pCFSAN017963_01	100	98.18
NODE_20_length_99538_cov_23.3276	DP141_17605	MchC protein	1	CP017721.1	Salmonella enterica subsp. enterica serovar Minnesota strain CFSAN017963 plasmid pCFSAN017963_01	100	99.94

NODE_20_length_99538_cov_23.3276	DP141_17610	toxin-activating lysine-acyltransferase	1	CP017721.1	Salmonella enterica subsp. enterica serovar Minnesota strain CFSAN017963 plasmid pCFSAN017963_01	100	99.79
NODE_20_length_99538_cov_23.3276	DP141_17615	HlyD family efflux transporter periplasmicadaptor subunit	1	CP017721.1	Salmonella enterica subsp. enterica serovar Minnesota strain CFSAN017963 plasmid pCFSAN017963_01	100	99.92
NODE_20_length_99538_cov_23.3276	DP141_17620	colicin V synthesis protein	1	CP017721.1	Salmonella enterica subsp. enterica serovar Minnesota strain CFSAN017963 plasmid pCFSAN017963_01	100	99.95
NODE_20_length_99538_cov_23.3276	DP141_17625	hypothetical protein	1	CP017721.1	Salmonella enterica subsp. enterica serovar Minnesota strain CFSAN017963 plasmid pCFSAN017963_01	100	99.66
NODE_20_length_99538_cov_23.3276	DP141_17630	CPBP family intramembrane metalloprotease	1	JQ418521.1	Salmonella sp. 96A-29192 plasmid p96A29192-65	100	100
NODE_20_length_99538_cov_23.3276	DP141_17660	AbrB/MazE/SpoVT family DNA-bindingdomain-containing protein	0.5325	CP006609.1	Salmonella bongori N268-08 plasmid RM1	100	83.55
NODE_20_length_99538_cov_23.3276	DP141_17665	PIN domain-containing protein	1	CP026170.1	Leclercia sp. LSNIH1 plasmid pLEC-000f	98.8	79.61
NODE_20_length_99538_cov_23.3276	DP141_17670	hypothetical protein	1				
NODE_20_length_99538_cov_23.3276	DP141_17675	IS3 family transposase	1	CP029729.1	Citrobacter sp. CRE-46 strain AR_0157 plasmid unnamed1	100	96.91
NODE_20_length_99538_cov_23.3276	DP141_17680	Tn3 family transposase	1	CP009995.1	Yersinia pestis strain Java9 plasmid1	100	93.99
NODE_20_length_99538_cov_23.3276	DP141_17685	hypothetical protein	1	LS483428.1	Salmonella enterica strain NCTC10436 genome assembly	95.37	82.52

NODE_20_length_99538_cov_23.3276	DP141_17690	hypothetical protein	1	CP022660.1	Salmonella enterica subsp. enterica strain RM11060 plasmid pRM11060-2	75.13	89.86
NODE_20_length_99538_cov_23.3276	DP141_17695	dipeptidase	1	LS483478.1	Salmonella enterica subsp. houtenae serovar Houten strain NCTC10401 genome assembly	100	96.86
NODE_20_length_99538_cov_23.3276	DP141_17700	leucine efflux protein LeuE	1	CP019889.1	Enterobacter cloacae strain FRM	60.8	84.11
NODE_20_length_99538_cov_23.3276	DP141_17705	C-lysozyme inhibitor	1	CP022660.1	Salmonella enterica subsp. enterica strain RM11060 plasmid pRM11060-2	100	95.36
NODE_20_length_99538_cov_23.3276	DP141_17710	DUF1090 domain-containing protein	0.9948	CP022660.1	Salmonella enterica subsp. enterica strain RM11060 plasmid pRM11060-2	100	99.48
NODE_20_length_99538_cov_23.3276	DP141_17715	hypothetical protein	1	FN298495.1	Salmonella enterica subsp. VII integrative and conjugative element ICESe3 region	94.03	90.87
NODE_30_length_79362_cov_25.6159	DP141_21750	filamentous hemagglutinin	0.9757	CP017976.1	Salmonella enterica subsp. enterica serovar Montevideo str. CDC 2011K-1674 chromosome	100	91.15
NODE_30_length_79362_cov_25.6159	DP141_21755	ShlB/FhaC/HecB family hemolysinsecretion/activation protein	1	CP017974.1	Salmonella enterica subsp. enterica serovar Montevideo str. CDC 07-0954 chromosome	100	91.76
NODE_30_length_79362_cov_25.6159	DP141_21760	hypothetical protein	1	CP017975.1	Salmonella enterica subsp. enterica serovar Montevideo str. CDC 08-1942 chromosome	100	95.17
NODE_30_length_79362_cov_25.6159	DP141_21765	hypothetical protein	1	CP017975.1	Salmonella enterica subsp. enterica serovar Montevideo str. CDC 08-1942 chromosome	100	93.26
NODE_30_length_79362_cov_25.6159	DP141_21770	hypothetical protein	1	FN298495.1	Salmonella enterica subsp. VII integrative and conjugative element ICESe3 region	99.19	97.81

NODE_30_length_79362_cov_25.6159	DP141_21775	hypothetical protein	1	LN890520.1	Salmonella enterica subsp. enterica serovar Weltevreden genome assembly C2346	100	95.56
NODE_30_length_79362_cov_25.6159	DP141_21780	hypothetical protein	1	CP017974.1	Salmonella enterica subsp. enterica serovar Montevideo str. CDC 07-0954 chromosome	100	96.59
NODE_30_length_79362_cov_25.6159	DP141_21785	hypothetical protein	1	CP017974.1	Salmonella enterica subsp. enterica serovar Montevideo str. CDC 07-0954 chromosome	100	97.21
NODE_30_length_79362_cov_25.6159	DP141_21790	hypothetical protein	1	CP016014.1	Salmonella enterica subsp. enterica serovar Newport strain CFSAN003387	100	97.6
NODE_30_length_79362_cov_25.6159	DP141_21795	hypothetical protein	1	CP017975.1	Salmonella enterica subsp. enterica serovar Montevideo str. CDC 08-1942 chromosome	100	98.64
NODE_30_length_79362_cov_25.6159	DP141_21800	hypothetical protein	1	CP016014.1	Salmonella enterica subsp. enterica serovar Newport strain CFSAN003387	100	97.78
NODE_30_length_79362_cov_25.6159	DP141_21805	hypothetical protein	1	CP017974.1	Salmonella enterica subsp. enterica serovar Montevideo str. CDC 07-0954 chromosome	100	99.05
NODE_30_length_79362_cov_25.6159	DP141_21810	hypothetical protein	1	CP017974.1	Salmonella enterica subsp. enterica serovar Montevideo str. CDC 07-0954 chromosome	100	97.29
NODE_30_length_79362_cov_25.6159	DP141_21815	hypothetical protein	1	CP017975.1	Salmonella enterica subsp. enterica serovar Montevideo str. CDC 08-1942 chromosome	100	98.44
NODE_30_length_79362_cov_25.6159	DP141_21820	hypothetical protein	1	CP024165.1	Salmonella enterica subsp. enterica serovar Gaminara strain CFSAN070644 chromosome	97.62	78.59
NODE_30_length_79362_cov_25.6159	DP141_21825	DNA-binding protein	1	LS483456.1	Salmonella enterica subsp. salamae strain NCTC9930 genome assembly	99.02	82.02

NODE_30_length_79362_cov_25.6159	DP141_21830	hypothetical protein	1	LS483456.1	Salmonella enterica subsp. salamae strain NCTC9930 genome assembly	98.41	80.65
NODE_30_length_79362_cov_25.6159	DP141_21835	hypothetical protein	1	CP017975.1	Salmonella enterica subsp. enterica serovar Montevideo str. CDC 08-1942 chromosome	100	99.84
NODE_30_length_79362_cov_25.6159	DP141_21840	DUF1187 family protein	1	FN298495.1	Salmonella enterica subsp. VII integrative and conjugative element ICESe3 region	100	98.97
NODE_30_length_79362_cov_25.6159	DP141_21845	hypothetical protein	1	CP017975.1	Salmonella enterica subsp. enterica serovar Montevideo str. CDC 08-1942 chromosome	100	97.37
NODE_30_length_79362_cov_25.6159	DP141_21850	hypothetical protein	1	CP017975.1	Salmonella enterica subsp. enterica serovar Montevideo str. CDC 08-1942 chromosome	96.72	96.05
NODE_30_length_79362_cov_25.6159	DP141_21855	conjugal transfer protein TraG	1	CP017974.1	Salmonella enterica subsp. enterica serovar Montevideo str. CDC 07-0954 chromosome	91.11	95.7
NODE_30_length_79362_cov_25.6159	DP141_21860	hypothetical protein	1	CP017975.1	Salmonella enterica subsp. enterica serovar Montevideo str. CDC 08-1942 chromosome	100	92.97
NODE_30_length_79362_cov_25.6159	DP141_21865	integrating conjugative element protein	1	CP017975.1	Salmonella enterica subsp. enterica serovar Montevideo str. CDC 08-1942 chromosome	100	96.66
NODE_30_length_79362_cov_25.6159	DP141_21870	TIGR03756 family integrating conjugative elementprotein	1	CP022658.1	Salmonella enterica subsp. enterica strain RM11060 chromosome	100	93.17
NODE_30_length_79362_cov_25.6159	DP141_21875	TIGR03757 family integrating conjugative elementprotein	1	CP022658.1	Salmonella enterica subsp. enterica strain RM11060 chromosome	100	97.46
NODE_30_length_79362_cov_25.6159	DP141_21880	hypothetical protein	1	CP016014.1	Salmonella enterica subsp. enterica serovar Newport strain CFSAN003387	50.76	70.9

NODE_30_length_79362_cov_25.6159	DP141_21885	hypothetical protein	1	CP016014.1	Salmonella enterica subsp. enterica serovar Newport strain CFSAN003387	100	95.48
NODE_30_length_79362_cov_25.6159	DP141_21890	conjugative transfer ATPase	1	CP022658.1	Salmonella enterica subsp. enterica strain RM11060 chromosome	99.96	94.08
NODE_30_length_79362_cov_25.6159	DP141_21895	TIGR03751 family conjugal transfer lipoprotein	1	CP024165.1	Salmonella enterica subsp. enterica serovar Gaminara strain CFSAN070644 chromosome	100	100
NODE_30_length_79362_cov_25.6159	DP141_21900	TIGR03752 family integrating conjugative elementprotein	1	CP024165.1	Salmonella enterica subsp. enterica serovar Gaminara strain CFSAN070644 chromosome	99.6	99.6
NODE_30_length_79362_cov_25.6159	DP141_21905	TIGR03749 family integrating conjugative elementprotein	1	CP024165.1	Salmonella enterica subsp. enterica serovar Gaminara strain CFSAN070644 chromosome	100	99.77
NODE_30_length_79362_cov_25.6159	DP141_21910	TIGR03746 family integrating conjugative elementprotein	1	CP024165.1	Salmonella enterica subsp. enterica serovar Gaminara strain CFSAN070644 chromosome	100	95.78
NODE_30_length_79362_cov_25.6159	DP141_21915	TIGR03750 family conjugal transfer protein	1	CP022658.1	Salmonella enterica subsp. enterica strain RM11060 chromosome	100	98.72
NODE_30_length_79362_cov_25.6159	DP141_21920	TIGR03745 family integrating conjugative elementmembrane protein	1	CP017976.1	Salmonella enterica subsp. enterica serovar Montevideo str. CDC 2011K-1674 chromosome	100	100
NODE_30_length_79362_cov_25.6159	DP141_21925	TIGR03758 family integrating conjugative elementprotein	1	CP024165.1	Salmonella enterica subsp. enterica serovar Gaminara strain CFSAN070644 chromosome	100	98.73
NODE_30_length_79362_cov_25.6159	DP141_21930	integrative conjugative element protein, RAQPRDfamily	1	CP017975.1	Salmonella enterica subsp. enterica serovar Montevideo str. CDC 08-1942 chromosome	100	97.22
NODE_30_length_79362_cov_25.6159	DP141_21935	TIGR03747 family integrating conjugative elementmembrane protein	1	CP024165.1	Salmonella enterica subsp. enterica serovar Gaminara strain CFSAN070644 chromosome	100	98.3

NODE_30_length_79362_cov_25.6159	DP141_21940	conjugative coupling factor TraD, PFGI-1 class	1	CP017976.1	Salmonella enterica subsp. enterica serovar Montevideo str. CDC 2011K-1674 chromosome	99.86	92.92
NODE_30_length_79362_cov_25.6159	DP141_21945	hypothetical protein	1	LN890520.1	Salmonella enterica subsp. enterica serovar Weltevreden genome assembly C2346	98.59	94.15
NODE_30_length_79362_cov_25.6159	DP141_21950	restriction endonuclease	1	CP016014.1	Salmonella enterica subsp. enterica serovar Newport strain CFSAN003387	79.37	92.81
NODE_30_length_79362_cov_25.6159	DP141_21955	integrating conjugative element protein	1	CP017976.1	Salmonella enterica subsp. enterica serovar Montevideo str. CDC 2011K-1674 chromosome	100	94.73
NODE_30_length_79362_cov_25.6159	DP141_21960	lytic transglycosylase domain-containingprotein	1	CP022658.1	Salmonella enterica subsp. enterica strain RM11060 chromosome	100	98.26
NODE_30_length_79362_cov_25.6159	DP141_21965	TIGR03759 family integrating conjugative elementprotein	1	CP017976.1	Salmonella enterica subsp. enterica serovar Montevideo str. CDC 2011K-1674 chromosome	100	98.25
NODE_30_length_79362_cov_25.6159	DP141_21970	hypothetical protein	1	CP017975.1	Salmonella enterica subsp. enterica serovar Montevideo str. CDC 08-1942 chromosome	100	96.96
NODE_30_length_79362_cov_25.6159	DP141_21975	hypothetical protein	1	LS483474.1	Salmonella enterica subsp. diarizonae strain NCTC10381 genome assembly	28.42	85.19
NODE_30_length_79362_cov_25.6159	DP141_21980	hypothetical protein	1	CP024165.1	Salmonella enterica subsp. enterica serovar Gaminara strain CFSAN070644 chromosome	100	94.19
NODE_30_length_79362_cov_25.6159	DP141_21985	hypothetical protein	1	CP024165.1	Salmonella enterica subsp. enterica serovar Gaminara strain CFSAN070644 chromosome	93.33	81.43
NODE_30_length_79362_cov_25.6159	DP141_21990	ead/Ea22-like family protein	1	CP019174.1	Salmonella enterica subsp. enterica serovar Give strain CFSAN024229 chromosome	82.38	90.7

NODE_30_length_79362_cov_25.6159	DP141_21995	hypothetical protein	1	CP024165.1	Salmonella enterica subsp. enterica serovar Gaminara strain CFSAN070644 chromosome	97.79	98.71
NODE_30_length_79362_cov_25.6159	DP141_22000	conjugal transfer protein TraF	1	CP016014.1	Salmonella enterica subsp. enterica serovar Newport strain CFSAN003387	100	98.44
NODE_30_length_79362_cov_25.6159	DP141_22005	hypothetical protein	1	CP022658.1	Salmonella enterica subsp. enterica strain RM11060 chromosome	100	98.98
NODE_30_length_79362_cov_25.6159	DP141_22010	hypothetical protein	1	CP022658.1	Salmonella enterica subsp. enterica strain RM11060 chromosome	100	98.29
NODE_30_length_79362_cov_25.6159	DP141_22015	integrase	1	CP022658.1	Salmonella enterica subsp. enterica strain RM11060 chromosome	100	98.44
NODE_30_length_79362_cov_25.6159	DP141_22020	phage tail protein	1	CP022658.1	Salmonella enterica subsp. enterica strain RM11060 chromosome	99.46	99.46
NODE_30_length_79362_cov_25.6159	DP141_22025	shufflon protein D'	1	CP022658.1	Salmonella enterica subsp. enterica strain RM11060 chromosome	100	98.65
NODE_30_length_79362_cov_25.6159	DP141_22030	hypothetical protein	1	CP022658.1	Salmonella enterica subsp. enterica strain RM11060 chromosome	100	99.65
NODE_30_length_79362_cov_25.6159	DP141_22035	Shufflon protein B	1	CP017975.1	Salmonella enterica subsp. enterica serovar Montevideo str. CDC 08-1942 chromosome	100	98.02
NODE_30_length_79362_cov_25.6159	DP141_22040	prepilin peptidase	1	CP017976.1	Salmonella enterica subsp. enterica serovar Montevideo str. CDC 2011K-1674 chromosome	100	97.73
NODE_30_length_79362_cov_25.6159	DP141_22045	pilus assembly protein	1	CP016014.1	Salmonella enterica subsp. enterica serovar Newport strain CFSAN003387	99.78	98.22

NODE_30_length_79362_cov_25.6159	DP141_22050	pilus assembly protein PilX	1	CP016014.1	Salmonella enterica subsp. enterica serovar Newport strain CFSAN003387	99.63	99.63
NODE_30_length_79362_cov_25.6159	DP141_22055	pilus assembly protein PilR	1	CP016014.1	Salmonella enterica subsp. enterica serovar Newport strain CFSAN003387	100	98.52
NODE_30_length_79362_cov_25.6159	DP141_22060	pilus assembly protein	1	CP016014.1	Salmonella enterica subsp. enterica serovar Newport strain CFSAN003387	100	97.32
NODE_30_length_79362_cov_25.6159	DP141_22065	type IV pilus biogenesis protein PilP	1	FN298495.1	Salmonella enterica subsp. VII integrative and conjugative element ICESe3 region	100	96.8
NODE_30_length_79362_cov_25.6159	DP141_22070	pilus assembly protein	1	CP022658.1	Salmonella enterica subsp. enterica strain RM11060 chromosome	100	97.29
NODE_30_length_79362_cov_25.6159	DP141_22075	PilN family type IVB pilus formation outermembrane protein	1	CP022658.1	Salmonella enterica subsp. enterica strain RM11060 chromosome	100	97.66
NODE_30_length_79362_cov_25.6159	DP141_22080	pilus assembly protein PilP	1	CP017975.1	Salmonella enterica subsp. enterica serovar Montevideo str. CDC 08-1942 chromosome	100	98.17
NODE_30_length_79362_cov_25.6159	DP141_22085	hypothetical protein	1	CP016014.1	Salmonella enterica subsp. enterica serovar Newport strain CFSAN003387	100	95.93
NODE_30_length_79362_cov_25.6159	DP141_22090	O-acetyl-ADP-ribose deacetylase	1	CP024165.1	Salmonella enterica subsp. enterica serovar Gaminara strain CFSAN070644 chromosome	100	98.72
NODE_30_length_79362_cov_25.6159	DP141_22095	ISNCY family transposase	1	CP024165.1	Salmonella enterica subsp. enterica serovar Gaminara strain CFSAN070644 chromosome	100	98.8
NODE_30_length_79362_cov_25.6159	DP141_22100	single-stranded DNA-binding protein	1	CP024165.1	Salmonella enterica subsp. enterica serovar Gaminara strain CFSAN070644 chromosome	100	99.63

NODE_30_length_79362_cov_25.6159	DP141_22105	hypothetical protein	1	CP024165.1	Salmonella enterica subsp. enterica serovar Gaminara strain CFSAN070644 chromosome	100	96.8
NODE_30_length_79362_cov_25.6159	DP141_22110	DUF3577 domain-containing protein	1	CP022658.1	Salmonella enterica subsp. enterica strain RM11060 chromosome	100	99.33
NODE_30_length_79362_cov_25.6159	DP141_22115	DNA topoisomerase III	1	CP022658.1	Salmonella enterica subsp. enterica strain RM11060 chromosome	100	97.77
NODE_30_length_79362_cov_25.6159	DP141_22120	TIGR03761 family integrating conjugative elementprotein	1	CP022658.1	Salmonella enterica subsp. enterica strain RM11060 chromosome	99.86	97.67
NODE_30_length_79362_cov_25.6159	DP141_22125	hypothetical protein	1	CP022658.1	Salmonella enterica subsp. enterica strain RM11060 chromosome	100	96.3
NODE_30_length_79362_cov_25.6159	DP141_22130	helix-turn-helix domain-containing protein	1	CP022658.1	Salmonella enterica subsp. enterica strain RM11060 chromosome	100	98.99
NODE_30_length_79362_cov_25.6159	DP141_22135	hypothetical protein	1	CP022658.1	Salmonella enterica subsp. enterica strain RM11060 chromosome	100	100
NODE_30_length_79362_cov_25.6159	DP141_22140	DUF2857 domain-containing protein	1	CP022658.1	Salmonella enterica subsp. enterica strain RM11060 chromosome	100	100
NODE_30_length_79362_cov_25.6159	DP141_22145	DUF2786 domain-containing protein	1	CP022658.1	Salmonella enterica subsp. enterica strain RM11060 chromosome	100	99.03
NODE_30_length_79362_cov_25.6159	DP141_22150	hypothetical protein	1	CP017976.1	Salmonella enterica subsp. enterica serovar Montevideo str. CDC 2011K-1674 chromosome	100	95.83
NODE_30_length_79362_cov_25.6159	DP141_22155	hypothetical protein	1	CP017976.1	Salmonella enterica subsp. enterica serovar Montevideo str. CDC 2011K-1674 chromosome	100	97.33

NODE_30_length_79362_cov_25.6159	DP141_22160	hypothetical protein	1	CP022658.1	Salmonella enterica subsp. enterica strain RM11060 chromosome	100	94.89
NODE_30_length_79362_cov_25.6159	DP141_22165	replicative DNA helicase	1	CP022658.1	Salmonella enterica subsp. enterica strain RM11060 chromosome	100	98.39
NODE_30_length_79362_cov_25.6159	DP141_22170	hypothetical protein	1	CP022658.1	Salmonella enterica subsp. enterica strain RM11060 chromosome	100	100
NODE_30_length_79362_cov_25.6159	DP141_22175	ParA family protein	1	CP022658.1	Salmonella enterica subsp. enterica strain RM11060 chromosome	100	100
NODE_39_length_43652_cov_188.42	DP141_24510	protelomerase	1	CP012557.1	Raoultella ornithinolytica strain 18 plasmid 2	84.59	83.3
NODE_39_length_43652_cov_188.42	DP141_24515	transposase	1	FO704551.1	Xenorhabdus poinarii str. G6 chromosome	98.43	68.82
NODE_39_length_43652_cov_188.42	DP141_24520	hypothetical protein	1				
NODE_39_length_43652_cov_188.42	DP141_24525	host cell division inhibitor Icd-like protein	1	CP015504.1	Klebsiella pneumoniae strain SKGH01 plasmid unnamed 4	99.39	82.32
NODE_39_length_43652_cov_188.42	DP141_24530	hypothetical protein	1	CP025712.1	Escherichia phage YDC107_1 chromosome	90.1	91.33
NODE_39_length_43652_cov_188.42	DP141_24535	hypothetical protein	1	CP012557.1	Raoultella ornithinolytica strain 18 plasmid 2	99.72	80.32
NODE_39_length_43652_cov_188.42	DP141_24540	XRE family transcriptional regulator	1	CP012557.1	Raoultella ornithinolytica strain 18 plasmid 2	100	82.48

NODE_39_length_43652_cov_188.42	DP141_24545	Cro/Cl family transcriptional regulator	1	CP012557.1	Raoultella ornithinolytica strain 18 plasmid 2	95.65	81.31
NODE_39_length_43652_cov_188.42	DP141_24550	hypothetical protein	1	CP012557.1	Raoultella ornithinolytica strain 18 plasmid 2	99.67	79.5
NODE_39_length_43652_cov_188.42	DP141_24555	hypothetical protein	1	CP012557.1	Raoultella ornithinolytica strain 18 plasmid 2	97.13	74.63
NODE_39_length_43652_cov_188.42	DP141_24560	hypothetical protein	1	CP012557.1	Raoultella ornithinolytica strain 18 plasmid 2	99.67	79.08
NODE_39_length_43652_cov_188.42	DP141_24565	hypothetical protein	1	CP012557.1	Raoultella ornithinolytica strain 18 plasmid 2	100	71.99
NODE_39_length_43652_cov_188.42	DP141_24570	hypothetical protein	1	CP012557.1	Raoultella ornithinolytica strain 18 plasmid 2	67.16	77.78
NODE_39_length_43652_cov_188.42	DP141_24575	3'-5' exonuclease	1	CP012557.1	Raoultella ornithinolytica strain 18 plasmid 2	90.29	67.72
NODE_39_length_43652_cov_188.42	DP141_24580	hypothetical protein	1	CP026178.1	Klebsiella pneumoniae strain KPNIH49 chromosome	93.39	69.58
NODE_39_length_43652_cov_188.42	DP141_24585	hypothetical protein	1	LS483466.1	Salmonella enterica subsp. arizonae strain NCTC7307 genome assembly	100	95.67
NODE_39_length_43652_cov_188.42	DP141_24590	hypothetical protein	1	LS483466.1	Salmonella enterica subsp. arizonae strain NCTC7307 genome assembly	100	98.41
NODE_39_length_43652_cov_188.42	DP141_24595	ead/Ea22-like family protein	1	LT904868.1	Salmonella enterica subsp. enterica serovar Typhi strain H12ESR04734-001A genome assembly	98.8	98.38

NODE_39_length_43652_cov_188.42	DP141_24600	hypothetical protein	1	CP019179.1	Salmonella enterica subsp. enterica serovar Dublin str. ATCC 39184	100	98.88
NODE_39_length_43652_cov_188.42	DP141_24605	DUF550 domain-containing protein	0.8086	CP019417.1	Salmonella enterica subsp. enterica serovar Wandsworth str. SA20092095	97.84	90.22
NODE_39_length_43652_cov_188.42	DP141_24610	hypothetical protein	1	CP012557.1	Raoultella ornithinolytica strain 18 plasmid 2	87.28	69.85
NODE_39_length_43652_cov_188.42	DP141_24615	hypothetical protein	1	CP012557.1	Raoultella ornithinolytica strain 18 plasmid 2	99.21	75.04
NODE_39_length_43652_cov_188.42	DP141_24620	DinI family protein	1				
NODE_39_length_43652_cov_188.42	DP141_24625	hypothetical protein	1	CP012557.1	Raoultella ornithinolytica strain 18 plasmid 2	78.31	77.95
NODE_39_length_43652_cov_188.42	DP141_24630	XRE family transcriptional regulator	1	AF064539.1	AF064539 Bacteriophage N15	83.33	70.21
NODE_39_length_43652_cov_188.42	DP141_24635	type II toxin-antitoxin system RelE/ParE familytoxin	1	CP028992.1	Klebsiella pneumoniae strain AR_0142 plasmid unnamed2	99.06	75.87
NODE_39_length_43652_cov_188.42	DP141_24640	hypothetical protein	1	CP012557.1	Raoultella ornithinolytica strain 18 plasmid 2	100	77.48
NODE_39_length_43652_cov_188.42	DP141_24645	hypothetical protein	1	CP012557.1	Raoultella ornithinolytica strain 18 plasmid 2	85.83	71.36
NODE_39_length_43652_cov_188.42	DP141_24650	site-specific DNA-methyltransferase	1	CP026270.1	Klebsiella oxytoca strain KONIH4 plasmid unnamed	99.63	77.49

NODE_39_length_43652_cov_188.42	DP141_24655	hypothetical protein	1	AY374448.1	Bacteriophage phiKO2	97.73	79.14
NODE_39_length_43652_cov_188.42	DP141_24660	hypothetical protein	1	CP012557.1	Raoultella ornithinolytica strain 18 plasmid 2	97.97	79.88
NODE_39_length_43652_cov_188.42	DP141_24665	hypothetical protein	1	AY374448.1	Bacteriophage phiKO2	100	78.49
NODE_39_length_43652_cov_188.42	DP141_24670	hypothetical protein	1				
NODE_39_length_43652_cov_188.42	DP141_24675	nucleoside 2-deoxyribosyltransferase	1	CP026270.1	Klebsiella oxytoca strain KONIH4 plasmid unnamed	94.5	80.48
NODE_39_length_43652_cov_188.42	DP141_24680	DNA cytosine methyltransferase	1	AF064539.1	AF064539 Bacteriophage N15	68.19	79.67
NODE_39_length_43652_cov_188.42	DP141_24685	HNH endonuclease	1	CP022034.1	Salmonella enterica subsp. enterica serovar Onderstepoort str. SA20060086	96.69	83.19
NODE_39_length_43652_cov_188.42	DP141_24690	terminase	1	CP021942.1	Klebsiella pneumoniae strain AR_0145 plasmid tig00000218j2847_linear	92.64	81.64
NODE_39_length_43652_cov_188.42	DP141_24695	terminase large subunit	1	CP026760.1	Klebsiella aerogenes strain AR_0062 plasmid unnamed4	99.71	80.87
NODE_39_length_43652_cov_188.42	DP141_24700	hypothetical protein	1	CP026760.1	Klebsiella aerogenes strain AR_0062 plasmid unnamed4	95.03	75.58
NODE_39_length_43652_cov_188.42	DP141_24705	phage portal protein	0.9857	CP006608.1	Salmonella bongori N268-08	99.13	88.23

NODE_39_length_43652_cov_188.42	DP141_24710	hypothetical protein	0.8006				
NODE_39_length_43652_cov_188.42	DP141_24715	S49 family peptidase	1	CP006608.1	Salmonella bongori N268-08	82.14	98.72
NODE_39_length_43652_cov_188.42	DP141_24720	phage major capsid protein	1	CP006608.1	Salmonella bongori N268-08	100	96.66
NODE_39_length_43652_cov_188.42	DP141_24725	hypothetical protein	1	CP022034.1	Salmonella enterica subsp. enterica serovar Onderstepoort str. SA20060086	100	95.7
NODE_39_length_43652_cov_188.42	DP141_24735	head-tail adaptor protein	1	CP022034.1	Salmonella enterica subsp. enterica serovar Onderstepoort str. SA20060086	100	98.84
NODE_39_length_43652_cov_188.42	DP141_24740	hypothetical protein	1	CP022034.1	Salmonella enterica subsp. enterica serovar Onderstepoort str. SA20060086	100	97.69
NODE_39_length_43652_cov_188.42	DP141_24745	hypothetical protein	1	CP022034.1	Salmonella enterica subsp. enterica serovar Onderstepoort str. SA20060086	100	99.5
NODE_39_length_43652_cov_188.42	DP141_24750	phage tail protein	1	CP022034.1	Salmonella enterica subsp. enterica serovar Onderstepoort str. SA20060086	100	96.41
NODE_39_length_43652_cov_188.42	DP141_24755	phage tail protein	1	CP022034.1	Salmonella enterica subsp. enterica serovar Onderstepoort str. SA20060086	100	92.29
NODE_39_length_43652_cov_188.42	DP141_24760	hypothetical protein	1	CP022034.1	Salmonella enterica subsp. enterica serovar Onderstepoort str. SA20060086	100	95.67
NODE_39_length_43652_cov_188.42	DP141_24765	phage tail tape measure protein	1	CP006608.1	Salmonella bongori N268-08	100	95.49

NODE_39_length_43652_cov_188.42	DP141_24770	phage tail protein	1	CP006608.1	Salmonella bongori N268-08	100	95.8
NODE_39_length_43652_cov_188.42	DP141_24775	phage minor tail protein L	0.7256	CP006608.1	Salmonella bongori N268-08	99.14	96.09
NODE_39_length_43652_cov_188.42	DP141_24780	hypothetical protein	1				
NODE_46_length_12144_cov_19.2682	DP141_25930	DUF1090 domain-containing protein	0.975	CP022660.1	Salmonella enterica subsp. enterica strain RM11060 plasmid pRM11060-2	100	98.75
NODE_46_length_12144_cov_19.2682	DP141_25935	nucleoside transporter	1	CP016014.1	Salmonella enterica subsp. enterica serovar Newport strain CFSAN003387	100	97.55
NODE_46_length_12144_cov_19.2682	DP141_25940	hypothetical protein	1	CP022660.1	Salmonella enterica subsp. enterica strain RM11060 plasmid pRM11060-2	100	97.77
NODE_46_length_12144_cov_19.2682	DP141_25945	phosphoadenosine phosphosulfate reductase	1	CP022660.1	Salmonella enterica subsp. enterica strain RM11060 plasmid pRM11060-2	97.81	96.43
NODE_46_length_12144_cov_19.2682	DP141_25950	hypothetical protein	1	CP022660.1	Salmonella enterica subsp. enterica strain RM11060 plasmid pRM11060-2	91.45	79.5
NODE_46_length_12144_cov_19.2682	DP141_25955	CaiF/GrlA family transcriptional regulator	1	CP016014.1	Salmonella enterica subsp. enterica serovar Newport strain CFSAN003387	100	93.41
NODE_46_length_12144_cov_19.2682	DP141_25960	hypothetical protein	1	CP022660.1	Salmonella enterica subsp. enterica strain RM11060 plasmid pRM11060-2	99.61	92.52
NODE_46_length_12144_cov_19.2682	DP141_25965	DUF2919 domain-containing protein	1	CP022660.1	Salmonella enterica subsp. enterica strain RM11060 plasmid pRM11060-2	100	96.82

NODE_46_length_12144_cov_19.2682	DP141_25970	cobalt ABC transporter permease	1	CP022660.1	Salmonella enterica subsp. enterica strain RM11060 plasmid pRM11060-2	100	95.22
NODE_47_length_11462_cov_22.6341	DP141_25975	hypothetical protein	1	CP017975.1	Salmonella enterica subsp. enterica serovar Montevideo str. CDC 08-1942 chromosome	35.09	82.77
NODE_47_length_11462_cov_22.6341	DP141_25980	hypothetical protein	0.9966	CP019193.1	Salmonella enterica subsp. enterica serovar Rubislaw str. ATCC 10717 plasmid pATCC10717	63.24	88.71
NODE_47_length_11462_cov_22.6341	DP141_25985	cobalt ABC transporter permease	1	CP022660.1	Salmonella enterica subsp. enterica strain RM11060 plasmid pRM11060-2	97.76	89.71
NODE_47_length_11462_cov_22.6341	DP141_25990	DUF2919 domain-containing protein	1	CP022016.1	Salmonella enterica subsp. enterica serovar India str. SA20085604 plasmid unnamed1	100	92.39
NODE_47_length_11462_cov_22.6341	DP141_25995	hypothetical protein	1	CP019193.1	Salmonella enterica subsp. enterica serovar Rubislaw str. ATCC 10717 plasmid pATCC10717	100	90.98
NODE_47_length_11462_cov_22.6341	DP141_26000	CaiF/GrlA family transcriptional regulator	1	CP019193.1	Salmonella enterica subsp. enterica serovar Rubislaw str. ATCC 10717 plasmid pATCC10717	100	85.08
NODE_47_length_11462_cov_22.6341	DP141_26005	hypothetical protein	1	CP019193.1	Salmonella enterica subsp. enterica serovar Rubislaw str. ATCC 10717 plasmid pATCC10717	100	91.8
NODE_47_length_11462_cov_22.6341	DP141_26010	phosphoadenosine phosphosulfate reductase	1	CP019193.1	Salmonella enterica subsp. enterica serovar Rubislaw str. ATCC 10717 plasmid pATCC10717	100	94.48
NODE_47_length_11462_cov_22.6341	DP141_26015	hypothetical protein	1	CP019193.1	Salmonella enterica subsp. enterica serovar Rubislaw str. ATCC 10717 plasmid pATCC10717	100	89.73
NODE_47_length_11462_cov_22.6341	DP141_26020	nucleoside transporter	1	CP019193.1	Salmonella enterica subsp. enterica serovar Rubislaw str. ATCC 10717 plasmid pATCC10717	100	90.52

NODE_47_length_11462_cov_22.6341	DP141_26025	DUF1090 domain-containing protein	1	CP019193.1	Salmonella enterica subsp. enterica serovar Rubislaw str. ATCC 10717 plasmid pATCC10717	100	85.19
NODE_47_length_11462_cov_22.6341	DP141_26030	IS256 family transposase	1	LS483475.1	Salmonella enterica subsp. salamae serovar Greenside strain NCTC9936 genome assembly	97.38	92.62
NODE_48_length_11243_cov_21.9392	DP141_26040	holin	0.9286	CP029980.1	Escherichia coli strain 99-3165 plasmid unnamed2	100	97.62
NODE_49_length_6776_cov_231.761	DP141_26110	Tn3 family transposase	1	CP005992.1	Enterobacter sp. R4-368 plasmid pENT01	100	94.74
NODE_49_length_6776_cov_231.761	DP141_26115	recombinase family protein	1	CP028488.1	Yersinia massiliensis strain GTA plasmid unnamed1	100	93.64
NODE_49_length_6776_cov_231.761	DP141_26120	arsenate reductase (glutaredoxin)	1	CP009995.1	Yersinia pestis strain Java9 plasmid1	100	98.12
NODE_49_length_6776_cov_231.761	DP141_26125	arsenical efflux pump membrane protein ArsB	0.8426	CP009995.1	Yersinia pestis strain Java9 plasmid1	100	98.06
NODE_49_length_6776_cov_231.761	DP141_26130	transcriptional regulator	1	CP000826.1	Serratia proteamaculans 568	100	83.18
NODE_4_length_205299_cov_22.6698	DP141_05950	site-specific integrase	1	CP022658.1	Salmonella enterica subsp. enterica strain RM11060 chromosome	98.64	87.45
NODE_4_length_205299_cov_22.6698	DP141_05955	hypothetical protein	1	FN298495.1	Salmonella enterica subsp. VII integrative and conjugative element ICESe3 region	99.57	96.61
NODE_4_length_205299_cov_22.6698	DP141_05960	DNA helicase	1	FN298495.1	Salmonella enterica subsp. VII integrative and conjugative element ICESe3 region	100	97.26

NODE_4_length_205299_cov_22.6698	DP141_05965	ShlB/FhaC/HecB family hemolysinsecretion/activation protein	1	CP014137.1	Brenneria goodwinii strain FRB141	59.36	66.37
NODE_4_length_205299_cov_22.6698	DP141_05970	ATP-binding protein	1	CP022503.1	Salmonella enterica subsp. enterica serovar Hvittingfoss str. SA20014981 chromosome	100	90.36
NODE_4_length_205299_cov_22.6698	DP141_05975	IS21 family transposase	0.9786	CP025981.1	Escherichia marmotae strain HT073016 plasmid pEM76	91.38	90.92
NODE_4_length_205299_cov_22.6698	DP141_05980	DNA polymerase V subunit UmuC	1	FN298495.1	Salmonella enterica subsp. VII integrative and conjugative element ICESe3 region	98.67	99.32
NODE_4_length_205299_cov_22.6698	DP141_05985	DNA-binding protein	1	CP017976.1	Salmonella enterica subsp. enterica serovar Montevideo str. CDC 2011K-1674 chromosome	99.85	97.86
NODE_4_length_205299_cov_22.6698	DP141_05990	sugar phosphate isomerase/epimerase	1	CP017976.1	Salmonella enterica subsp. enterica serovar Montevideo str. CDC 2011K-1674 chromosome	99.67	98.69
NODE_4_length_205299_cov_22.6698	DP141_05995	molecular chaperone	1	CP022015.1	Salmonella enterica subsp. enterica serovar India str. SA20085604	86.35	99.26
NODE_4_length_205299_cov_22.6698	DP141_06000	hypothetical protein	1	CP022015.1	Salmonella enterica subsp. enterica serovar India str. SA20085604	98.94	98.39
NODE_4_length_205299_cov_22.6698	DP141_06005	hypothetical protein	1	CP022015.1	Salmonella enterica subsp. enterica serovar India str. SA20085604	100	98.97
NODE_4_length_205299_cov_22.6698	DP141_06010	hypothetical protein	0.8152	CP022015.1	Salmonella enterica subsp. enterica serovar India str. SA20085604	100	99.47
NODE_4_length_205299_cov_22.6698	DP141_06015	TonB-dependent siderophore receptor	0.6547	CP022015.1	Salmonella enterica subsp. enterica serovar India str. SA20085604	100	99.82

NODE_4_length_205299_cov_22.6698	DP141_06020	IS66 family transposase	1	LS483466.1	Salmonella enterica subsp. arizonae strain NCTC7307 genome assembly	99.15	81.35
NODE_4_length_205299_cov_22.6698	DP141_06025	hypothetical protein	1	LS483456.1	Salmonella enterica subsp. salamae strain NCTC9930 genome assembly	97.93	82.57
NODE_4_length_205299_cov_22.6698	DP141_06030	hypothetical protein	1	LS483456.1	Salmonella enterica subsp. salamae strain NCTC9930 genome assembly	68.57	82.14
NODE_4_length_205299_cov_22.6698	DP141_06035	arsenical resistance protein ArsH	1	KX810825.1	Salmonella enterica subsp. enterica serovar Typhimurium strain MU1 plasmid pIMP4-SEM1	100	91.9
NODE_4_length_205299_cov_22.6698	DP141_06040	transcriptional regulator	1	LT994835.1	Klebsiella pneumoniae isolate CNR48 genome assembly	100	88.79
NODE_4_length_205299_cov_22.6698	DP141_06045	arsenical efflux pump membrane protein ArsB	1	KY863418.1	Enterobacter asburiae strain AMA 497 plasmid pOXA436	100	95.74
NODE_4_length_205299_cov_22.6698	DP141_06050	arsenate reductase (glutaredoxin)	1	LT994835.1	Klebsiella pneumoniae isolate CNR48 genome assembly	100	96.48
NODE_4_length_205299_cov_22.6698	DP141_06055	universal stress protein	1	Y13308.1	Yersinia enterocolitica plasmid DNA fragment	100	92.16
NODE_4_length_205299_cov_22.6698	DP141_06060	sodium-independent anion transporter	1	CP007734.1	Klebsiella pneumoniae subsp. pneumoniae KPNIH27 plasmid pKPN-262	100	93.58
NODE_4_length_205299_cov_22.6698	DP141_06065	recombinase family protein	1	CP009995.1	Yersinia pestis strain Java9 plasmid1	99.83	89.27
NODE_4_length_205299_cov_22.6698	DP141_06070	Tn3 family transposase	1	CP022151.1	Citrobacter freundii strain 705SK3	100	94.15

NODE_4_length_205299_cov_22.6698	DP141_06075	IS110 family transposase	1	CP022015.1	Salmonella enterica subsp. enterica serovar India str. SA20085604	100	90.14
NODE_4_length_205299_cov_22.6698	DP141_06080	IS5/IS1182 family transposase	1	CP021852.1	Proteus mirabilis strain AR_0156	98.04	90
NODE_51_length_5681_cov_27.8054	DP141_26175	cytochrome c-type biogenesis protein CcmH	0.5558	CP020922.1	Salmonella enterica subsp. enterica strain 16A242	100	100
NODE_51_length_5681_cov_27.8054	DP141_26180	IS4 family transposase	1	LS483474.1	Salmonella enterica subsp. diarizonae strain NCTC10381 genome assembly	100	100
NODE_51_length_5681_cov_27.8054	DP141_26185	IS3 family transposase	1	CP022117.1	Salmonella enterica subsp. enterica serovar Macclesfield str. S-1643	100	93.51
NODE_51_length_5681_cov_27.8054	DP141_26190	hypothetical protein	1				
NODE_51_length_5681_cov_27.8054	DP141_26195	hypothetical protein	0.9991	CP029725.1	Proteus mirabilis strain AR_0029 chromosome	84.03	71.11
NODE_51_length_5681_cov_27.8054	DP141_26200	hypothetical protein	1	CP026203.1	Escherichia coli strain ECONIH5 plasmid pECO-a7e8	50	77.78
NODE_55_length_4547_cov_184.656	DP141_26320	UDP-N-acetylmuramate dehydrogenase	1	CP021462.1	Salmonella enterica subsp. enterica serovar Typhimurium strain UGA14 chromosome	100	100
NODE_55_length_4547_cov_184.656	DP141_26335	host cell division inhibitor Icd-like protein	1	CP015504.1	Klebsiella pneumoniae strain SKGH01 plasmid unnamed 4	99.39	81.1
NODE_55_length_4547_cov_184.656	DP141_26340	hypothetical protein	1	CP024433.1	Klebsiella pneumoniae strain DA48896 plasmid p48896_4	76.52	94.32

NODE_55_length_4547_cov_184.656	DP141_26345	hypothetical protein	1	CP024433.1	Klebsiella pneumoniae strain DA48896 plasmid p48896_4	36.87	87.67
NODE_55_length_4547_cov_184.656	DP141_26350	plasmid-partitioning protein SopA	1	AJ564013.1	Bacteriophage PY54 complete genome	96.76	72.54
NODE_55_length_4547_cov_184.656	DP141_26355	ParB/RepB/Spo0J family plasmid partitionprotein	1	CP007639.1	Salmonella enterica subsp. enterica serovar Choleraesuis strain C500	90.61	95.7
NODE_55_length_4547_cov_184.656	DP141_26360	hypothetical protein	1	CP009565.1	Salmonella enterica subsp. enterica serovar Newport str. CVM 21554	100	100
NODE_55_length_4547_cov_184.656	DP141_26365	DNA polymerase V	1	CP009565.1	Salmonella enterica subsp. enterica serovar Newport str. CVM 21554	99.59	99.18
NODE_56_length_4450_cov_15.1251	DP141_26370	DNA-invertase	1	CP009565.1	Salmonella enterica subsp. enterica serovar Newport str. CVM 21554	99.83	97.65
NODE_56_length_4450_cov_15.1251	DP141_26375	filamentous hemagglutinin	0.9583	CP017976.1	Salmonella enterica subsp. enterica serovar Montevideo str. CDC 2011K-1674 chromosome	98.81	87.54
NODE_59_length_3479_cov_223.619	DP141_26440	DNA breaking-rejoining protein	0.6895	CP028729.1	Salmonella enterica subsp. enterica serovar Thompson strain HFCDC-SM-846 chromosome	100	100
NODE_59_length_3479_cov_223.619	DP141_26455	tail fiber assembly protein	0.6754	CP007584.2	Salmonella enterica subsp. enterica serovar Anatum str. USDA-ARS-USMARC-1735	100	98.46
NODE_59_length_3479_cov_223.619	DP141_26460	phage tail protein	1	CP014707.1	Salmonella enterica subsp. enterica serovar Anatum strain USMARC-1735 plasmid pSAN1-1735	99.86	95.27
NODE_68_length_1444_cov_129.298	DP141_26615	lysozyme	0.7111	LS483493.1	Salmonella enterica subsp. enterica serovar Thompson strain NCTC8496 genome assembly	100	100

NODE_68_length_1444_cov_129.298	DP141_26620	hypothetical protein	1				
NODE_71_length_1315_cov_129.168	DP141_26650	hypothetical protein	1	CP015924.1	Salmonella enterica subsp. enterica serovar Newport str. Levine 15	100	100
NODE_72_length_1313_cov_57.6939	DP141_26655	IS256 family transposase	1	CP028813.1	Edwardsiella ictaluri strain MS-17-156 chromosome	100	94.62
NODE_74_length_1154_cov_108.318	DP141_26665	elongation factor Tu	0.9871	CP021462.1	Salmonella enterica subsp. enterica serovar Typhimurium strain UGA14 chromosome	100	100
NODE_79_length_740_cov_42.3687	DP141_26695	DNA breaking-rejoining protein	0.8496	CP017974.1	Salmonella enterica subsp. enterica serovar Montevideo str. CDC 07-0954 chromosome	100	100

**Table S5. GenBank locus tags and accession numbers for the genes included in the functional groups tested in Table 1. All locus tags and accessions refer to S. Typhimurium strain CDC 2011K-0870, GenBank:GCA_000973645.1.**			
**Gene Name**	**Functional Group**	**Locus Tag**	**Accession**
sitA	SPI-1	AX05_35770	AKD09505.1
sitB	SPI-1	AX05_35780	AKD09506.1
sitC	SPI-1	AX05_35790	AKD09507.1
sitD	SPI-1	AX05_35800	AKD09508.1
avrA/yopJ	SPI-1	AX05_35810	AKD09509.1
sprB	SPI-1	AX05_35820	AKD09510.1
hilC	SPI-1	AX05_35830	AKD09511.1
orgC	SPI-1	AX05_35840	AKD09512.1
orgA	SPI-1	AX05_35850	AKD09513.1
	SPI-1	AX05_35860	AKD09514.1
prgK	SPI-1	AX05_35870	AKD09515.1
prgJ	SPI-1	AX05_35880	AKD09516.1
prgI	SPI-1	AX05_35890	AKD09517.1
prgH	SPI-1	AX05_35900	AKD09518.1
hilD	SPI-1	AX05_35910	AKD09519.1
hilA	SPI-1	AX05_35920	AKD09520.1
iagB	SPI-1	AX05_35930	AKD09521.1
sptP/stpP	SPI-1	AX05_35940	AKD09522.1
sicP	SPI-1	AX05_35950	AKD09523.1
	SPI-1	AX05_35960	AKD09524.1
iacP	SPI-1	AX05_35970	AKD09525.1
sipA	SPI-1	AX05_35980	AKD09526.1
sipD	SPI-1	AX05_35990	AKD09527.1
sipC	SPI-1	AX05_36000	AKD09528.1
sipB	SPI-1	AX05_36010	AKD09529.1

sicA	SPI-1	AX05_36020	AKD09530.1
spaS	SPI-1	AX05_36030	AKD09531.1
spaR	SPI-1	AX05_36040	AKD09532.1
spaQ	SPI-1	AX05_36050	AKD09533.1
spaP	SPI-1	AX05_36060	AKD09534.1
spaO	SPI-1	AX05_36070	AKD09535.1
invJ/spaN	SPI-1	AX05_36080	AKD09536.1
invI/spaM	SPI-1	AX05_36090	AKD09537.1
invC	SPI-1	AX05_36100	AKD09538.1
invB/spaK	SPI-1	AX05_36110	AKD09539.1
invA	SPI-1	AX05_36120	AKD09540.1
invE	SPI-1	AX05_36130	AKD09541.1
invG	SPI-1	AX05_36140	AKD09542.1
invF	SPI-1	AX05_36150	AKD09543.1
invH	SPI-1	AX05_36160	AKD09544.1
slrP	SPI-1 effector	AX05_15500	AKD07481.1
sopB	SPI-1 effector	AX05_18110	AKD07742.1
sopE2	SPI-1 effector	AX05_26040	AKD08534.1
sopD	SPI-1 effector	AX05_36630	AKD09591.1
sopA	SPI-1 effector	AX05_28150	AKD08745.1
pipB2	SPI-1 effector	AX05_34900	AKD09418.1
orf48	SPI-2	AX05_21100	AKD08040.1
orf32	SPI-2	AX05_21110	AKD08041.1
orf245	SPI-2	AX05_21120	AKD08042.1
orf408	SPI-2	AX05_21130	AKD08043.1
ttrA	SPI-2	AX05_21140	AKD08044.1
ttrC	SPI-2	AX05_21150	AKD08045.1
ttrB	SPI-2	AX05_21160	AKD08046.1
ttrS	SPI-2	AX05_21170	AKD08047.1
ttrR	SPI-2	AX05_21180	AKD08048.1
orf70	SPI-2	AX05_21190	AKD08049.1
orf319	SPI-2	AX05_21200	AKD08050.1

orf242	SPI-2	AX05_21220	AKD08052.1
ssrB	SPI-2	AX05_21230	AKD08053.1
ssrA	SPI-2	AX05_21240	AKD08054.1
ssaC	SPI-2	AX05_21250	AKD08055.1
ssaD	SPI-2	AX05_21260	AKD08056.1
ssaE	SPI-2	AX05_21270	AKD08057.1
sseB	SPI-2	AX05_21280	AKD08058.1
sscA	SPI-2	AX05_21290	AKD08059.1
sseC	SPI-2	AX05_21300	AKD08060.1
sseD	SPI-2	AX05_21310	AKD08061.1
sseE	SPI-2	AX05_21320	AKD08062.1
sscB	SPI-2	AX05_21330	AKD08063.1
sseF	SPI-2	AX05_21340	AKD08064.1
sseG	SPI-2	AX05_21350	AKD08065.1
ssaG	SPI-2	AX05_21360	AKD08066.1
ssaH	SPI-2	AX05_21370	AKD08067.1
ssaI	SPI-2	AX05_21380	AKD08068.1
ssaJ	SPI-2	AX05_21390	AKD08069.1
	SPI-2	AX05_21400	AKD08070.1
ssaK	SPI-2	AX05_21410	AKD08071.1
ssaL	SPI-2	AX05_21420	AKD08072.1
ssaM	SPI-2	AX05_21430	AKD08073.1
ssaV	SPI-2	AX05_21440	AKD08074.1
ssaN	SPI-2	AX05_21450	AKD08075.1
ssaO	SPI-2	AX05_21460	AKD08076.1
ssaP	SPI-2	AX05_21470	AKD08077.1
ssaQ	SPI-2	AX05_21480	AKD08078.1
ssaR	SPI-2	AX05_21490	AKD08079.1
ssaS	SPI-2	AX05_21500	AKD08080.1
ssaT	SPI-2	AX05_21510	AKD08081.1
ssaU	SPI-2	AX05_21520	AKD08082.1
fimA	Type 1 fimbrial cluster	AX05_12990	AKD07230.1
fimI	Type 1 fimbrial cluster	AX05_13000	AKD07231.1

fimC	Type 1 fimbrial cluster	AX05_13010	AKD07232.1
fimD	Type 1 fimbrial cluster	AX05_13020	AKD07233.1
fimH	Type 1 fimbrial cluster	AX05_13030	AKD07234.1
fimF	Type 1 fimbrial cluster	AX05_13040	AKD07235.1
fimZ	Type 1 fimbrial cluster	AX05_13050	AKD07236.1
fimY	Type 1 fimbrial cluster	AX05_13060	AKD07237.1
fimW	Type 1 fimbrial cluster	AX05_13080	AKD07239.1

**Table S6. Non-synonymous mutations along the phylogenetic branch leading to the L. olivacea S. Typhimurium isolates.**								
**Locus Tag^**	**Protein Accession^**	**Coding DNA-level**			**Protein-level**			**NCBI 'Product' Annotation^**
		**Position**	**Ancestral**	**Derived**	**Position**	**Ancestral**	**Derived**	
AX05_50	AKD05946.1	1487	T	A	496	Gln	Leu	ATPase ravA
AX05_500	AKD05989.1	1097	C	G	366	Gly	Ala	uroporphyrinogen-III C-methyltransferase
AX05_2050	AKD06142.1	265	G	A	89	Ile	Val	Glycerol kinase
AX05_2820	AKD06216.1	637	C	T	213	Ser	Pro	Endonuclease V
AX05_3480	AKD06281.1	325	A	C	109	Pro	Thr	Maltose-binding periplasmic protein
AX05_4120	AKD06345.1	680	A	G	227	Gly	Glu	Sensor protein BasS
AX05_4120	AKD06345.1	493	C	A	165	Ser	Arg	Sensor protein BasS
AX05_4760	AKD06409.1	949	G	A	317	Thr	Ala	transport protein
AX05_5250	AKD06458.1	1099	C	A	367	Ile	Leu	2',3'-cyclic-nucleotide 2'-phosphodiesterase/3'-nucleotidase
AX05_5340	AKD06467.1	3746	A	C	1249	Pro	Gln	protein ytfN
AX05_5620	AKD06495.1	947	G	A	316	Gln	Arg	Protein pmbA
AX05_5880	AKD06521.1	349	C	A	117	Met	Leu	Arginine repressor 1
AX05_6820	AKD06615.1	329	G	T	110	Val	Gly	Diguanylate cyclase
AX05_8390	AKD06772.1	1112	T	A	371	Gln	Leu	Thiamine transport system permease protein thiP

AX05_8820	AKD06815.1	310	T	A	104	Arg	Trp	Nicotinate-nucleotide pyrophosphorylase
AX05_8950	AKD06828.1	977	C	T	326	Val	Ala	hypothetical protein
AX05_10640	AKD06995.1	394	G	A	132	Lys	Glu	Outer membrane pore protein E
AX05_11370	AKD07068.1	166	C	T	56	Cys	Arg	protein yaiA
AX05_11500	AKD07081.1	1236	A	C	412	Asn	Lys	Proline-specific permease ProY
AX05_12330	AKD07164.1	686	G	A	229	His	Arg	ISNCY transposase
AX05_12560	AKD07187.1	253	A	C	85	Pro	Thr	Inner membrane protein ybbJ
AX05_12810	AKD07212.1	196	G	A	66	Ile	Val	Glycerate kinase 1
AX05_12830	AKD07214.1	524	C	T	175	Val	Ala	Allantoate amidohydrolase
AX05_12990	AKD07230.1	304	G	C	102	Pro	Ala	Fimbrial subunit type 1
AX05_13030	AKD07234.1	578	T	C	193	Ala	Val	Protein fimH
AX05_13030	AKD07234.1	881	C	A	294	Gln	Pro	Protein fimH
AX05_13700	AKD07301.1	646	G	T	216	Phe	Val	zinc-type alcohol dehydrogenase-like protein YbdR
AX05_13840	AKD07315.1	400	C	G	134	Val	Leu	Lipid A palmitoyltransferase PagP
AX05_14320	AKD07363.1	1107	A	T	369	Asn	Lys	N-acetylglucosamine repressor
AX05_14410	AKD07372.1	1093	G	A	365	Thr	Ala	Citrate utilization protein B
AX05_14450	AKD07376.1	537	A	C	179	Asp	Glu	Flavodoxin

AX05_14700	AKD07401.1	5	T	C	2	Ser	Leu	inner membrane protein
AX05_14760	AKD07407.1	302	C	T	101	Leu	Pro	ABC transporter domain protein
AX05_15110	AKD07442.1	539	G	C	180	Ala	Gly	Phospho-2-dehydro-3-deoxyheptonate aldolase
AX05_16160	AKD07547.1	680	G	A	227	His	Arg	Soluble aldose sugar dehydrogenase yliI
AX05_16180	AKD07549.1	322	T	G	108	Ala	Ser	D-alanyl-D-alanine carboxypeptidase DacC
AX05_16510	AKD07582.1	964	G	T	322	Cys	Gly	protein ybjT
AX05_16530	AKD07584.1	1150	A	G	384	Ala	Thr	Pyruvate dehydrogenase [ubiquinone]
AX05_16620	AKD07593.1	108	G	A	36	Ile	Met	Cold shock-like protein CspD
AX05_17160	AKD07647.1	38	T	C	13	Ala	Val	hypothetical protein
AX05_17330	AKD07664.1	587	C	T	196	Val	Ala	Gifsy-1 prophage PrpO
AX05_17510	AKD07682.1	667	G	T	223	Ser	Ala	Phage portal protein, lambda
AX05_17600	AKD07691.1	823	G	A	275	Arg	Gly	prophage tail component protein
AX05_17700	AKD07701.1	112	G	A	38	Met	Val	Side tail fiber protein
AX05_17700	AKD07701.1	377	C	T	126	Val	Ala	Side tail fiber protein
AX05_17700	AKD07701.1	839	C	A	280	Glu	Ala	Side tail fiber protein
AX05_17830	AKD07714.1	1249	C	T	417	Ser	Pro	ABC transporter ATP-binding protein uup
AX05_18080	AKD07739.1	194	T	C	65	Pro	Leu	GogA

AX05_18 520	AKD07783.1	229	G	A	77	Ile	Val	Oligogalacturonate-specific porin
AX05_18 540	AKD07785.1	102	T	A	34	Glu	Asp	reductase
AX05_18 630	AKD07794.1	551	C	A	184	Glu	Ala	CsgBAC operon transcriptional regulatory protein
AX05_18 720	AKD07803.1	1063	T	A	355	Ser	Cys	Glucans biosynthesis glucosyltransferase H
AX05_18 900	AKD07821.1	100	G	A	34	Met	Val	hypothetical protein
AX05_19 110	AKD07842.1	88	G	T	30	Tyr	Asp	Maf-like protein yceF 1
AX05_19 180	AKD07849.1	96	C	A	32	Glu	Asp	hypothetical protein
AX05_20 190	AKD07949.1	247	T	A	83	Arg	Trp	glucose-6-phosphate 1-epimerase
AX05_20 630	AKD07993.1	92	T	C	31	Ala	Val	O-antigen polymerase
AX05_20 890	AKD08019.1	203	C	T	68	Leu	Pro	protein ydiF
AX05_20 960	AKD08026.1	808	T	A	270	Ile	Phe	FAD linked oxidase, C-terminal domain protein
AX05_21 350	AKD08065.1	218	T	A	73	Lys	Ile	Secreted effector protein
AX05_22 280	AKD08158.1	1160	C	A	387	Glu	Ala	reductase
AX05_22 880	AKD08218.1	139	A	G	47	Gly	Ser	coiled-coil protein
AX05_22 930	AKD08223.1	2374	C	T	792	Tyr	His	TreY
AX05_23 000	AKD08230.1	1150	G	A	384	Asn	Asp	NAD-dependent malic enzyme
AX05_23 840	AKD08314.1	911	A	T	304	Leu	Gln	protein ydbH

AX05_24000	AKD08330.1	426	G	T	142	Phe	Leu	protein ydaL
AX05_24020	AKD08332.1	712	G	A	238	Thr	Ala	Anaerobic regulatory protein
AX05_24440	AKD08374.1	304	G	A	102	Ile	Val	Cyclic diguanylate phosphodiesterase (EAL) domain protein
AX05_24460	AKD08376.1	259	G	A	87	Arg	Gly	HTH-type transcriptional regulator
AX05_24970	AKD08427.1	122	T	G	41	Gly	Val	Protein hnr
AX05_25040	AKD08434.1	127	C	A	43	Met	Leu	reductase
AX05_25280	AKD08458.1	76	G	A	26	Ser	Gly	GTP-dependent nucleic acid-binding protein engD
AX05_25750	AKD08505.1	202	G	A	68	Ile	Val	hypothetical protein
AX05_25910	AKD08521.1	163	T	A	55	Thr	Ser	Carboxy-terminal proteinase
AX05_26160	AKD08546.1	31	G	A	11	Ile	Val	inner membrane protein
AX05_27140	AKD08644.1	1032	A	C	344	Asp	Glu	Cytoplasmic alpha-amylase
AX05_27690	AKD08699.1	407	G	A	136	Tyr	Cys	Cobalt import ATP-binding protein CbiO
AX05_27970	AKD08727.1	143	G	T	48	Leu	Arg	Propanediol utilization protein
AX05_28290	AKD08759.1	217	A	T	73	Tyr	Asn	Chain length determinant protein
AX05_28540	AKD08784.1	1094	T	C	365	Pro	Leu	Phosphomannomutase
AX05_28550	AKD08785.1	668	G	T	223	Leu	Arg	Mannose-1-phosphate guanylyltransferase
AX05_28760	AKD08806.1	475	G	T	159	Ser	Ala	DNA-3-methyladenine glycosylase 2

AX05_28960	AKD08826.1	874	C	A	292	Ile	Leu	nucleoside transporter yegT
AX05_29030	AKD08833.1	602	C	T	201	Val	Ala	protein yehA
AX05_29280	AKD08858.1	13	G	T	5	Ser	Ala	hypothetical protein
AX05_29510	AKD08881.1	629	G	C	210	Ala	Gly	L-serine ammonia-lyase
AX05_30270	AKD08957.1	2329	A	T	777	Cys	Ser	Sensor kinase protein RcsC
AX05_30570	AKD08987.1	1373	C	T	458	Val	Ala	Undecaprenyl phosphate-alpha-4-amino-4-deoxy-L-arabinose arabinosyl transferase
AX05_31120	AKD09042.1	463	A	G	155	Glu	Lys	Histidine-binding periplasmic protein
AX05_31140	AKD09044.1	26	A	G	9	Gly	Asp	hypothetical protein
AX05_31250	AKD09055.1	158	C	T	53	Ile	Thr	Bifunctional folylpolyglutamate synthase/ dihydrofolate synthase
AX05_31440	AKD09074.1	919	A	T	307	Tyr	Asn	Chorismate synthase
AX05_31570	AKD09087.1	923	T	G	308	Arg	Leu	Phosphoglycerate transport regulatory protein pgtC
AX05_31740	AKD09104.1	1832	C	T	611	Leu	Ser	protein YfeA
AX05_31820	AKD09112.1	340	G	A	114	Thr	Ala	protein yfeN
AX05_31820	AKD09112.1	677	G	A	226	Glu	Gly	protein yfeN
AX05_31840	AKD09114.1	677	G	C	226	Ser	Trp	protein yfeH
AX05_32000	AKD09130.1	599	C	T	200	Leu	Pro	Cysteine synthase B
AX05_32400	AKD09170.1	1313	T	G	438	Gly	Val	oxidoreductase

AX05_33 330	AKD09263.1	707	A	G	236	Gly	Asp	Membrane-bound lytic murein transglycosylase F
AX05_33 640	AKD09294.1	553	G	A	185	Ile	Val	DTW domain-containing protein yfiP
AX05_33 840	AKD09312.1	287	T	C	96	Ser	Phe	diguanylate cyclase YfiN
AX05_34 010	AKD09329.1	6140	A	G	2047	Ser	Asn	Large repetitive protein
AX05_34 900	AKD09418.1	701	T	A	234	Gln	Leu	Secreted effector protein pipB2
AX05_35 050	AKD09433.1	170	C	A	57	Asp	Ala	Succinic semialdehyde dehydrogenase
AX05_35 080	AKD09436.1	223	G	A	75	Lys	Glu	HTH-type transcriptional repressor CsiR
AX05_35 240	AKD09452.1	1429	G	A	477	Ser	Gly	reductase
AX05_35 610	AKD09489.1	755	C	T	252	Val	Ala	Formate hydrogenlyase subunit 7
AX05_35 810	AKD09509.1	167	C	G	56	Gly	Ala	Pathogenicity island membrane protein
AX05_35 830	AKD09511.1	5	T	C	2	Ala	Val	Transcriptional regulator sirC
AX05_35 910	AKD09519.1	398	T	G	133	Ser	Ile	Transcriptional regulator hilD
AX05_36 010	AKD09529.1	1556	C	T	519	Val	Ala	Cell invasion protein sipB
AX05_36 510	AKD09579.1	749	T	C	250	Ala	Val	Sulfate adenylyltransferase subunit 2 1
AX05_36 740	AKD09602.1	507	G	C	169	Asp	Glu	7-carboxy-7-deazaguanine synthase
AX05_36 860	AKD09614.1	822	G	C	274	Cys	Trp	Glucarate transporter
AX05_37 010	AKD09629.1	929	C	A	310	Glu	Ala	L-fucose-proton symporter

AX05_37510	AKD09679.1	20	T	A	7	His	Leu	Nickel/cobalt efflux system rcnA
AX05_37820	AKD09710.1	1237	C	T	413	Cys	Arg	Glycine dehydrogenase [decarboxylating] 1
AX05_38290	AKD09757.1	493	C	A	165	Ser	Arg	Regulatory protein, IclR
AX05_38450	AKD09773.1	665	C	T	222	Val	Ala	Nucleoside permease nupG
AX05_39110	AKD09839.1	765	C	A	255	Arg	Ser	Transcriptional regulator, AraC
AX05_40480	AKD09976.1	707	C	A	236	Lys	Thr	Polyprenyl synthetase
AX05_41270	AKD10055.1	799	G	A	267	Lys	Glu	Ribosomal protein L11 methyltransferase
AX05_41500	AKD10076.1	673	G	A	225	Lys	Glu	Ribosomal RNA small subunit methyltransferase B
AX05_42560	AKD10182.1	874	G	A	292	Arg	Gly	Maltodextrin phosphorylase
AX05_42860	AKD10212.1	373	C	G	125	Gly	Arg	reductase
AX05_43300	AKD10256.1	1208	C	T	403	Met	Thr	ABC transporter ATP-binding protein YhiH
AX05_43400	AKD10266.1	44	C	A	15	Glu	Ala	Phosphoesterase PA-phosphatase protein
AX05_43510	AKD10277.1	221	G	A	74	Tyr	Cys	Phage-like lysozyme
AX05_44010	AKD10327.1	325	A	G	109	Val	Met	HTH-type transcriptional regulator yiaG
AX05_44230	AKD10349.1	860	T	C	287	Ala	Val	2,3-diketo-L-gulonate TRAP transporter large permease protein yiaN
AX05_44410	AKD10367.1	658	G	T	220	Tyr	Asp	lipoprotein
AX05_44710	AKD10397.1	58	G	A	20	Thr	Ala	Lipopolysaccharide 1,3-galactosyltransferase

AX05_45040	AKD10430.1	1391	C	T	464	Val	Ala	protein yicH
AX05_45510	AKD10477.1	224	G	A	75	His	Arg	DeoX
^Locus tags, accessions and GenBank annotations are from *S. Typhimurium* CDC-2011K-0870 (GenBank: GCA_000973645.1)								
*Gene Ontology (GO) annotations assigned based on amino acid-level homology to S. Typhimurium str. LT2. S. Typhimurium str. LT2 annotations downloaded from AmiGO 2 (http://amigo.geneontology.org/amigo/landing) on 12/23/2018.								

Table S6 (Continued). Non-synonymous mutations along the phylogenetic branch leading to the L. olivacea S. Typhimurium isolates.

Locus Tag^	Protein Accession^	AmiGO Annotations*	Biological Process*		Molecular Function*		Cellular Component*	
		Gene/Product Name	Name	Accession	Name	Accession	Name	Accession
AX05_50	AKD05946.1							
AX05_500	AKD05989.1							
AX05_2050	AKD06142.1	Glycerol kinase	glycerol kinase activity;glycerol metabolic process	GO:0004370;GO:0006071	glycerol kinase activity	GO:0004370		
AX05_2820	AKD06216.1	Endonuclease V	endoribonuclease activity, producing 5'-phosphomonoesters;deoxyribonuclease V activity	GO:0016891;GO:0043737	endoribonuclease activity, producing 5'-phosphomonoesters;single-stranded RNA binding;deox	GO:0016891;GO:0003727;GO:0043737		

					yribonuclease V activity			
AX05_3480	AKD06281.1							
AX05_4120	AKD06345.1	Sensor protein BasS	phosphorelay signal transduction system	GO:0000160			phosphorelay signal transduction system;plasma membrane	GO:0000160; GO:0005886
AX05_4120	AKD06345.1	Sensor protein BasS	phosphorelay signal transduction system	GO:0000160			phosphorelay signal transduction system;plasma membrane	GO:0000160; GO:0005886
AX05_4760	AKD06409.1	Epoxyqueuosine reductase	queuosine biosynthetic process;epoxyqueuosine reductase activity	GO:0008616;GO:0052693	epoxyqueuosine reductase activity	GO:0052693		
AX05_5250	AKD06458.1							

AX05_5340	AKD06467.1	Putative periplasmic protein	protein secretion	GO:0009306			integral component of plasma membrane;TAM protein secretion complex	GO:0005887; GO:0097347
AX05_5620	AKD06495.1	Putative peptide maturation protein					cytosol	GO:0005829
AX05_5880	AKD06521.1							
AX05_6820	AKD06615.1	Putative diguanylate cyclase/phosphodiesterase domain 1 containing protein	cell adhesion involved in single-species biofilm formation;negative regulation of bacterial-type flagellum-dependent cell motility	GO:0043709;GO:1902201	diguanylate cyclase activity	GO:0052621	plasma membrane	GO:0005886
AX05_8390	AKD06772.1	Putative binding-protein-dependent transport system inner membrane component					plasma membrane	GO:0005886
AX05_8820	AKD06815.1	Nicotinate-	quinolinate catabolic	GO:0034213;GO:0009435	nicotinate-nucleotide	GO:0004514	cytoplasm	GO:0005737

		nucleotide pyrophosphorylase [carboxylating]	process;NAD biosynthetic process		diphosphorylase (carboxylating) activity			
AX05_8950	AKD06828.1							
AX05_10640	AKD06995.1							
AX05_11370	AKD07068.1							
AX05_11500	AKD07081.1							
AX05_12330	AKD07164.1	Putative transposase	DNA recombination;double-stranded DNA endodeoxyribonuclease activity	GO:0006310;GO:1990238	double-stranded DNA endodeoxyribonuclease activity	GO:1990238		
AX05_12560	AKD07187.1	Putative membrane protein implicated in regulation of membrane protease activity					plasma membrane	GO:0005886
AX05_12810	AKD07212.1							
AX05_12830	AKD07214.1							
AX05_12990	AKD07230.1	Type-1 fimbrial protein, A chain	pilus organization;cell adhesion;cell adhesion involved in single-species	GO:0043711;GO:0007155;GO:0043709			pilus	GO:0009289

			biofilm formation					
AX05_13030	AKD07234.1							
AX05_13030	AKD07234.1							
AX05_13700	AKD07301.1							
AX05_13840	AKD07315.1	Lipid A palmitoyltransferase PagP	lipid A biosynthetic process;lipid A biosynthetic process	GO:0009245;GO:0009245	palmitoyltransferase activity;palmitoyltransferase activity	GO:0016409;GO:0016409	cell outer membrane;cell outer membrane	GO:0009279; GO:0009279
AX05_14320	AKD07363.1							
AX05_14410	AKD07372.1							
AX05_14450	AKD07376.1							
AX05_14700	AKD07401.1							
AX05_14760	AKD07407.1							
AX05_15110	AKD07442.1	Phospho-2-dehydro-3-deoxyheptonate aldolase	aromatic amino acid family biosynthetic process	GO:0009073	3-deoxy-7-phosphoheptulonate synthase activity	GO:0003849	cytoplasm	GO:0005737

AX05_16160	AKD07547.1							
AX05_16180	AKD07549.1	D-alanyl-D-alanine carboxypeptidase penicillin-binding protein 6a	endopeptidase activity	GO:0004175	endopeptidase activity	GO:0004175		
AX05_16510	AKD07582.1							
AX05_16530	AKD07584.1							
AX05_16620	AKD07593.1							
AX05_17160	AKD07647.1							
AX05_17330	AKD07664.1							
AX05_17510	AKD07682.1							
AX05_17600	AKD07691.1							
AX05_17700	AKD07701.1							
AX05_17700	AKD07701.1							
AX05_17700	AKD07701.1							
AX05_17830	AKD07714.1							
AX05_18080	AKD07739.1							

AX05_18520	AKD07783.1	Putative outer membrane protein	oligosaccharide transport;porin activity	GO:0015772;GO:0015288	porin activity	GO:0015288	cell outer membrane;integral component of cell outer membrane	GO:0009279; GO:0045203
AX05_18540	AKD07785.1							
AX05_18630	AKD07794.1							
AX05_18720	AKD07803.1							
AX05_18900	AKD07821.1							
AX05_19110	AKD07842.1	Maf-like protein YceF			nucleoside-triphosphate diphosphatase activity	GO:0047429		
AX05_19180	AKD07849.1	Acyl carrier protein	acyl carrier activity;lipid A biosynthetic process	GO:0000036;GO:0009245	acyl carrier activity;acyl binding	GO:0000036;GO:0000035	cytosol	GO:0005829
AX05_20190	AKD07949.1							
AX05_20630	AKD07993.1							
AX05_20890	AKD08019.1							

AX05_20960	AKD08026.1	Putative oxidase	D-lactate dehydrogenase activity;lactate catabolic process;D-lactate dehydrogenase (cytochrome) activity	GO:0008720;GO:1903457;GO:0004458	flavin adenine dinucleotide binding;D-lactate dehydrogenase activity;D-lactate dehydrogenase (cytochrome) activity	GO:0050660;GO:0008720;GO:0004458		
AX05_21350	AKD08065.1							
AX05_22280	AKD08158.1	Putative dimethyl sulphoxide reductase, chain A1	anaerobic respiration;electron transfer activity	GO:0009061;GO:0009055	molybdenum ion binding;electron transfer activity	GO:0030151;GO:0009055	outer membrane-bounded periplasmic space	GO:0030288
AX05_22880	AKD08218.1							
AX05_22930	AKD08223.1	Putative glycosyl hydrolase	alpha-glucan catabolic process;trehalose biosynthetic process	GO:0030980;GO:0005992	(1,4)-alpha-D-glucan 1-alpha-D-glucosylmutase activity	GO:0047470		
AX05_23000	AKD08230.1	NAD-dependent malic enzyme	pyruvate metabolic process;malic enzyme	GO:0006090;GO:0004470;GO:0006108	malic enzyme activity	GO:0004470	cytosol	GO:0005829

			activity;malate metabolic process					
AX05_23840	AKD08314.1							
AX05_24000	AKD08330.1	Putative Smr domain protein	endodeoxyribonuclease activity	GO:0004520	endodeoxyribonuclease activity	GO:0004520		
AX05_24020	AKD08332.1	Fumarate and nitrate reduction regulatory protein	DNA-binding transcription factor activity;bacterial-type RNA polymerase transcriptional activator activity, sequence-specific DNA binding;positive regulation of transcription, DNA-templated	GO:0003700;GO:0001216;GO:0045893	DNA-binding transcription factor activity;transcription regulatory region sequence-specific DNA binding;bacterial-type RNA polymerase transcriptional activator activity, sequence-specific DNA binding	GO:0003700;GO:0000976;GO:0001216	cytosol;protein-DNA complex	GO:0005829;GO:0032993
AX05_24440	AKD08374.1							
AX05_24460	AKD08376.1							
AX05_24970	AKD08427.1	Regulator of RpoS	DNA-binding transcription factor	GO:0003700;GO:0000156;GO:0001216	DNA-binding transcription factor	GO:0003700;GO:0000156;GO:0000976;GO:0001216	phosphorelay	GO:0000156;GO:0032993;GO:0005829

			activity;phosphorelay response regulator activity;bacterial-type RNA polymerase transcriptional activator activity, sequence-specific DNA binding		activity;phosphorelay response regulator activity;transcription regulatory region sequence-specific DNA binding;bacterial-type RNA polymerase transcriptional activator activity, sequence-specific DNA binding		response regulator activity;protein-DNA complex;cytosol	
AX05_25040	AKD08434.1	Nitrate reductase 1, delta subunit	nitrate assimilation;chaperone-mediated protein complex assembly	GO:0042128;GO:0051131	metallochaperone activity	GO:0016530		
AX05_25280	AKD08458.1	Ribosome-binding ATPase YchF			ATPase activity	GO:0016887	cytoplasm	GO:0005737
AX05_25750	AKD08505.1	Putative inner membrane protein					plasma membrane	GO:0005886
AX05_25910	AKD08521.1	Tail-specific protease	signal transduction;e	GO:0007165;GO:0004175	endopeptidase activity	GO:0004175	outer membrane-	GO:0030288

			ndopeptidase activity				bounded periplasmic space	
AX05_26160	AKD08546.1							
AX05_27140	AKD08644.1							
AX05_27690	AKD08699.1	Cobalt import ATP-binding protein CbiO	cobalt ion transport;ATPase activity, coupled to transmembrane movement of substances	GO:0006824;GO:0042626	ATPase activity, coupled to transmembrane movement of substances;ATP binding	GO:0042626;GO:0005524	ATP-binding cassette (ABC) transporter complex;ATP-binding cassette (ABC) transporter complex;integral component of plasma	GO:0043190; GO:0043190; GO:0005887

							membrane	
AX05_27970	AKD08727.1							
AX05_28290	AKD08759.1	Chain length determinant protein	protein tyrosine kinase activity	GO:0004713	identical protein binding;protein tyrosine kinase activity	GO:0042802;GO:0004713	plasma membrane	GO:0005886
AX05_28540	AKD08784.1							
AX05_28550	AKD08785.1	Mannose-1-phosphate guanylyltransferase ManC	GDP-mannose biosynthetic process	GO:0009298	mannose-1-phosphate guanylyltransferase activity	GO:0004475		
AX05_28760	AKD08806.1	3-methyl-adenine DNA glycosylase II	DNA dealkylation involved in DNA repair;base-excision repair, AP site formation	GO:0006307;GO:0006285	DNA-7-methylguanine glycosylase activity;alkylated DNA binding;DNA-3-methyladenine glycosylase activity	GO:0043916;GO:0032131;GO:0008725	cytoplasm;protein-DNA complex	GO:0005737; GO:0032993
AX05_28960	AKD08826.1	Putative MFS family transport protein					integral component of plasma	GO:0005887

							membrane	
AX05_29030	AKD08833.1							
AX05_29280	AKD08858.1							
AX05_29510	AKD08881.1	Putative D-serine dehydratase			L-serine ammonia-lyase activity	GO:0003941		
AX05_30270	AKD08957.1							
AX05_30570	AKD08987.1							
AX05_31120	AKD09042.1							
AX05_31140	AKD09044.1							
AX05_31250	AKD09055.1	Dihydrofolate synthase/folylpolyglutamate synthase	dihydrofolate synthase activity;tetrahydrofolylpolyglutamate synthase activity	GO:0008841;GO:0004326	dihydrofolate synthase activity;tetrahydrofolylpolyglutamate synthase activity	GO:0008841;GO:0004326	cytoplasm	GO:0005737
AX05_31440	AKD09074.1	Chorismate synthase	aromatic amino acid family biosynthetic process;chorismate biosynthetic process	GO:0009073;GO:0009423	chorismate synthase activity;FMN binding	GO:0004107;GO:0010181	cytosol	GO:0005829
AX05_31570	AKD09087.1							

AX05_3 1740	AKD091 04.1							
AX05_3 1820	AKD091 12.1							
AX05_3 1820	AKD091 12.1							
AX05_3 1840	AKD091 14.1							
AX05_3 2000	AKD091 30.1	Cysteine synthase B	cysteine biosynthetic process from serine;cysteine synthase activity	GO:0006535;GO:000412 4	L-cysteine desulfhydrase activity;cystei ne synthase activity;pyrid oxal phosphate binding	GO:0080146;GO:00 04124;GO:0030170	cytopl asm	GO:0005737
AX05_3 2400	AKD091 70.1							
AX05_3 3330	AKD092 63.1	Membran e-bound lytic murein transglyco sylase F	peptidoglycan catabolic process	GO:0009253	lytic transglycosyl ase activity	GO:0008933	cell outer memb rane	GO:0009279
AX05_3 3640	AKD092 94.1							
AX05_3 3840	AKD093 12.1	Putative diguanylat e cyclase/ph osphodies terase	cell adhesion involved in single-species biofilm formation;neg ative regulation of bacterial-type	GO:0043709;GO:190220 1	diguanylate cyclase activity	GO:0052621	plasma memb rane	GO:0005886

			flagellum-dependent cell motility					
AX05_34010	AKD09329.1							
AX05_34900	AKD09418.1	Secreted effector protein PipB2			protein binding	GO:0005515		
AX05_35050	AKD09433.1	NADP-dependent succinate-semialdehyde dehydrogenase I	succinate-semialdehyde dehydrogenase [NAD(P)+] activity;succinate-semialdehyde dehydrogenase (NAD+) activity	GO:0009013;GO:0004777	succinate-semialdehyde dehydrogenase [NAD(P)+] activity;succinate-semialdehyde dehydrogenase (NAD+) activity	GO:0009013;GO:0004777		
AX05_35080	AKD09436.1							
AX05_35240	AKD09452.1	Ribonucleoside-diphosphate reductase 2 subunit alpha	ribonucleoside-diphosphate reductase activity, thioredoxin disulfide as acceptor;deoxyribonucleotide biosynthetic process	GO:0004748;GO:0009263	ribonucleoside-diphosphate reductase activity, thioredoxin disulfide as acceptor	GO:0004748	ribonucleoside-diphosphate reductase complex	GO:0005971
AX05_35610	AKD09489.1							
AX05_35810	AKD09509.1							

AX05_35830	AKD09511.1							
AX05_35910	AKD09519.1							
AX05_36010	AKD09529.1	Cell invasion protein SipB	protein localization to Golgi apparatus	GO:0034067				
AX05_36510	AKD09579.1							
AX05_36740	AKD09602.1							
AX05_36860	AKD09614.1							
AX05_37010	AKD09629.1							
AX05_37510	AKD09679.1	Nickel/cobalt efflux system RcnA	nickel cation transmembrane transporter activity;nickel cation homeostasis;response to nickel cation;cation efflux transmembrane transporter activity;response to cobalt ion	GO:0015099;GO:0035784;GO:0010045;GO:0046583;GO:0032025	nickel cation transmembrane transporter activity;cation efflux transmembrane transporter activity	GO:0015099;GO:0046583	plasma membrane	GO:0005886
AX05_37820	AKD09710.1	Glycine dehydrogenase	glycine decarboxylation via glycine	GO:0019464;GO:0004375	glycine binding;pyridoxal	GO:0016594;GO:0030170;GO:0004375	cytosol;glycine	GO:0005829;GO:0005960

		(decarboxylating)	cleavage system;glycine dehydrogenase (decarboxylating) activity		phosphate binding;glycine dehydrogenase (decarboxylating) activity		cleavage complex	
AX05_38290	AKD09757.1	Putative transcriptional regulator	negative regulation of transcription, DNA-templated;DNA-binding transcription factor activity	GO:0045892;GO:0003700	DNA-binding transcription factor activity;DNA binding	GO:0003700;GO:0003677		
AX05_38450	AKD09773.1	Nucleoside permease NupG	uridine transmembrane transporter activity;cytidine transmembrane transporter activity	GO:0015213;GO:0015212	uridine transmembrane transporter activity;cytidine transmembrane transporter activity	GO:0015213;GO:0015212	integral component of plasma membrane	GO:0005887
AX05_39110	AKD09839.1							
AX05_40480	AKD09976.1							
AX05_41270	AKD10055.1							
AX05_41500	AKD10076.1	Ribosomal RNA small subunit	rRNA base methylation;rRNA (cytosine-	GO:0070475;GO:0009383	rRNA (cytosine-C5-)-	GO:0009383	cytosol	GO:0005829

		methyltransferase B	C5-)-methyltransferase activity		methyltransferase activity			
AX05_42560	AKD10182.1	Alpha-1,4 glucan phosphorylase	glycogen catabolic process	GO:0005980	glycogen phosphorylase activity;pyridoxal phosphate binding	GO:0008184;GO:0030170	cytoplasm	GO:0005737
AX05_42860	AKD10212.1							
AX05_43300	AKD10256.1							
AX05_43400	AKD10266.1							
AX05_43510	AKD10277.1	Putative phage endolysin			lysozyme activity	GO:0003796		
AX05_44010	AKD10327.1							
AX05_44230	AKD10349.1	Putative DedA family, membrane protein	transmembrane transporter activity	GO:0022857	transmembrane transporter activity	GO:0022857	plasma membrane	GO:0005886
AX05_44410	AKD10367.1							
AX05_44710	AKD10397.1							
AX05_45040	AKD10430.1							
AX05_45510	AKD10477.1							

^Locus tags, accessions and GenBank annotations are from *S. Typhimurium* CDC-2011K-0870 (GenBank: GCA_000973645.1)							
*Gene Ontology (GO) annotations assigned based on amino acid-level homology to S. Typhimurium str. LT2. S. Typhimurium str. LT2 annotations downloaded from AmiGO 2 (http://amigo.geneontology.org/amigo/landing) on 12/23/2018.							
